# Voice and speech as digital biomarkers of mental health: a scoping review of clinical interpretability, data collection and translational readiness

**DOI:** 10.3389/fdgth.2026.1964389

**Published:** 2026-10-05

**Authors:** Ralph Justin Uy, Ibrahim Shaaban, Angel Arnaout

**Affiliations:** 1 Faculty of Medicine, University of British Columbia, Vancouver, BC, Canada; 2 Faculty of Science, McGill University, Montreal, QC, Canada; 3 Medical Informatics Division, Medical and Academic Affairs, Provincial Health Services Authority, Vancouver, BC, Canada

**Keywords:** ambient sensing, clinical interpretability, depression, digital phenotyping, machine learning, mental health, speech analysis, voice biomarkers

## Abstract

**Background:**

Voice and speech encode acoustic, linguistic, and interactional information associated with mental health. Translating these signals into clinically meaningful digital biomarkers depends not only on predictive performance but on how speech is elicited, how the target state is defined, whether findings generalize beyond the original dataset, and whether features can be measured reliably in real-world settings.

**Objective:**

To map voice, speech, language, and communication biomarkers studied across mental health outcomes and to examine three dimensions of translational readiness: clinical interpretability, evidentiary validity, and deployability.

**Methods:**

This scoping review followed Joanna Briggs Institute guidance and PRISMA-ScR. PubMed, PsycINFO, and Scopus were searched through June 18, 2026. Two reviewers independently screened records and charted data. Biomarkers were classified as directly clinically interpretable, software-derived with a perceptual correlate, or technical-only.

**Results:**

Of 1,524 records identified, 68 studies were included. Depression was examined in 56 studies (82%), followed by anxiety (*n* = 9), bipolar disorder or mania (*n* = 8), fatigue (*n* = 4), post-traumatic stress disorder (*n* = 3), stress (*n* = 2), and other outcomes including suicidal risk and loneliness (*n* = 10); no study examined burnout or emotional exhaustion. Controlled elicitation predominated (54/68, 79%) over naturalistic (16/68, 24%) and passive or ambient capture (3/68, 4%). One study focused on healthcare workers and one analyzed clinician–patient dyadic speech. Across depression studies, clinically interpretable patterns commonly included slower speech, more or longer pauses, lower or less variable pitch, reduced vocal intensity, flatter prosody, and reduced verbal output, although findings were not uniform. Validation on an independent or cross-dataset sample was reported by only 2 of 68 studies (3%).

**Conclusions:**

Voice-based mental health research identifies numerous candidate biomarkers, but translational readiness remains limited. Most evidence derives from depression and controlled speech tasks, and interpretability does not imply validity or deployability. Existing studies do not establish that candidate biomarkers remain valid in spontaneous, multispeaker, passive, or ambient clinical settings. Future work should prioritize validated outcome labels, longitudinal naturalistic capture, external validation, diverse populations, and explicit evaluation of interpretable and technical features across recording contexts.

## 1 Introduction

Speech is a complex behavioural output shaped by psychological processes, motor control, language, and social interaction. Mental health conditions may alter one or more of these processes and produce measurable changes in how people speak and in the language they use. Voice and speech have therefore attracted interest as digital biomarkers that could complement clinical interviews and symptom questionnaires. Prior reviews have established that acoustic and linguistic features can discriminate or correlate with several mental health states, that this literature is most developed for depression and suicide risk, and that heterogeneity is substantially greater for other psychiatric and psychological outcomes (1–4).

Advances in artificial intelligence, natural language processing, acoustic signal analysis, and machine learning have enabled extraction of less directly interpretable features, including mel-frequency cepstral coefficients (MFCCs), spectral and cepstral representations, i-vectors or related supervector representations, and learned audio embeddings (5). These features may contribute substantial predictive signal, but their numerical values generally lack a direct perceptual or plain-language clinical equivalent. An important translational question therefore remains insufficiently addressed: under what methodological conditions should an observed voice feature be considered clinically meaningful? A feature may be easily interpretable but weakly validated; technically predictive but difficult to explain; or reproducible in a standardized laboratory task but unreliable in spontaneous conversation (6). Data-collection design, the clinical validity of the outcome label, and the validation strategy are therefore integral to interpreting the relevance of a proposed voice biomarker rather than merely technical details of model development.

Accordingly, this scoping review approaches the literature through three related but non-equivalent dimensions of translational readiness. Clinical interpretability refers to whether a measured feature can be understood in clinically meaningful or perceptual terms. Evidentiary validity refers to the strength of the link between the feature and the intended mental health construct, including how that construct was defined and whether findings were validated beyond the development sample. Deployability refers to whether the feature can be measured reliably in the setting in which it is ultimately intended to be used. We map voice, speech, language, and communication biomarkers across mental health outcomes while examining clinical interpretability, speech-elicitation context, recording modality, outcome measurement, and validation strategy. Naturalistic, passive or ambient, and occupational applications are considered potential future settings for translation rather than settings in which validity has already been established.

## 2 Methods

### 2.1 Review design and reporting

This scoping review was conducted in accordance with Joanna Briggs Institute methodological guidance for scoping reviews and is reported according to the Preferred Reporting Items for Systematic Reviews and Meta-Analyses extension for Scoping Reviews (PRISMA-ScR) (7, 8). The protocol was registered on the Open Science Framework during data extraction on July 15, 2026 (https://osf.io/k789q/). Eligibility criteria were developed using the Population–Concept–Context framework.

### 2.2 Search strategy and information sources

A comprehensive literature search was conducted in PubMed, PsycINFO through EBSCO, and Scopus. Initial searches were conducted on June 10, 2026, and the final search update was completed on June 18, 2026. No restriction was placed on publication year. Searches were restricted to English-language publications.

The search combined three concepts: (1) voice and speech characteristics; (2) mental health and psychological outcomes; and (3) biomarker or computational analysis. Voice- and speech-related terms included combinations of voice biomarker, speech biomarker, vocal biomarker, voice feature, speech feature, acoustic feature, voice analysis, speech analysis, prosody, and paralinguistic. Mental health terms included depression, anxiety, psychological stress, psychological distress, post-traumatic stress disorder, burnout, emotional exhaustion, fatigue, and mental health. These were combined with terms related to biomarkers, prediction, detection, classification, screening, monitoring, machine learning, and artificial intelligence. Controlled vocabulary, including Medical Subject Headings, was used where applicable and syntax was adapted for each database. Complete database-specific strategies are provided in Supplementary Table S1. Reference lists of included studies and relevant reviews were also screened, and one additional eligible study was identified through citation searching during peer review.

### 2.3 Eligibility criteria

Studies involving human participants of any age or background were eligible, including clinical and nonclinical populations. Studies focused primarily on neurological, developmental, cognitive, or speech disorders were excluded when the purpose was to detect those conditions rather than to examine an eligible mental health or psychological outcome. Mixed populations remained eligible when at least one relevant mental health, distress, fatigue, burnout, or well-being outcome was assessed.

The core concept was an association between voice, speech, language, communication, or related digital-phenotyping features and a relevant mental health or psychological state. Both clinically understandable markers and technical computational features were eligible. Studies reporting only technical acoustic or machine-learning-derived measures were retained when those measures were evaluated against an eligible outcome.

Studies from any country and setting were eligible, including controlled research environments, naturalistic contexts, and passive or ambient recording. Original peer-reviewed empirical research was eligible. Reviews, protocols, opinion articles, dissertations, and conference abstracts without sufficient study data were excluded. Studies concerned only with speech recognition, transcription accuracy, speaker identification, speech synthesis, or neurological speech diagnosis were excluded unless they also evaluated an eligible mental health or well-being outcome.

For the purposes of this review, controlled elicitation referred to speech produced in response to a standardized or predetermined vocal task, such as sustained phonation, scripted reading, repetition, picture description, or structured questioning. Naturalistic elicitation referred to unscripted or minimally scripted conversational speech, such as free narrative, semi-structured interviewing, psychotherapy, or other real-world conversational speech, even when the recording was actively initiated for research. Passive or ambient capture referred to speech collected during routine activity without requiring the participant to complete a dedicated speech task. These categories describe the conditions under which speech was obtained and do not themselves imply ecological validity or clinical deployability.

### 2.4 Study selection

All identified records were imported into Covidence and duplicates were removed. Two reviewers (RJU and IS) independently screened titles and abstracts against the eligibility criteria. Records considered potentially relevant by either reviewer were retrieved for full-text assessment. RJU and IS then independently assessed all full-text reports. Disagreements were resolved through discussion, with AA serving as the third reviewer when consensus could not be reached.

### 2.5 Data charting

A standardized data extraction form was developed from the review questions and eligibility criteria. RJU and IS independently extracted data, compared entries, and resolved discrepancies through discussion. Extracted items included publication year, country, study design, sample size, participant characteristics, speaker role, elicitation context, recording modality, mental health outcome, outcome measure, biomarker domain, specific marker, analytic method, main findings, direction of association, model performance when applicable, and author-reported limitations. The full data extraction form is provided in Supplementary Table S2.

### 2.6 Clinical interpretability framework

A three-tier framework developed for this review was used to classify clinical interpretability rather than diagnostic accuracy or methodological quality. Features were classified as directly interpretable (Tier 1) when the underlying speech characteristic could be perceived or described in ordinary clinical terms without requiring a computational representation; as partially interpretable (Tier 2) when the exact numerical value required computational measurement but the measure mapped to a recognizable perceptual vocal quality; and as technical-only (Tier 3) when the feature represented a mathematical transformation or learned representation of the audio signal without a straightforward perceptual or plain-language equivalent. The full contents of each tier are shown in Figure 4. Because most studies examined features from more than one tier, a single study-level rating was assigned according to the feature class on which the study's principal reported finding rested — the features the authors identified as primary, best-performing, or on which the main conclusion depended — rather than the highest or lowest tier present in the feature set. Where the principal features were proprietary, or were reported only as an aggregate feature set without a designated primary feature, the study was rated technical-only. Domain-level counts in Table 1 and Figure 3 are independent of this study-level rating and include every domain a study examined. This framework classifies interpretability only. A higher level of interpretability was not assumed to indicate stronger validity, superior predictive performance, or greater deployability.

**Table 1:** Voice and speech biomarker domains, clinical interpretability, and direction of association with depression.

Biomarker domain	Interpretability	Studies (n)	Direction in depression, where reported
Speech rate	Yes	19	Generally slower
Pause duration or frequency	Yes	27	Generally longer or more frequent
Response latency	Yes	4	Generally longer
Pitch (F0)	Yes	47	Generally lower mean or reduced variability
Vocal intensity or loudness	Yes	38	Generally quieter
Prosody or rhythm	Yes	21	Generally flatter or less dynamic
Verbal output	Yes	23	Generally reduced
Voice quality	Yes	27	Variable; often poorer clarity or greater instability
Word or pronoun use	Yes	9	More self-focused and less positive language
Sentiment or emotional tone	Yes	8	Generally more negative
Communication behaviour	Yes	2	Shorter turns, more pausing, reduced floor control
Jitter	Partial	24	Variable; often increased
Shimmer	Partial	23	Variable; often increased
Harmonics-to-noise ratio	Partial	23	Variable; often decreased
Formant frequencies	Partial	28	Variable; altered vowel or articulation characteristics
MFCCs	No	37	No consistent direction across coefficients
Other technical features (incl. deep-learning embeddings and i-vectors)	No	56	Latent patterns without a directly interpretable direction

Studies examining more than one domain contribute to multiple rows. Study-level interpretability ratings were Yes in 29 studies (43%), Partial in 26 (38%) and No in 13 (19%).

### 2.7 Translational-methodological characterization

To examine translational readiness beyond feature interpretability, we additionally characterized the methodological context in which each biomarker association was reported. This was a descriptive extension of the scoping review rather than a formal risk-of-bias assessment or quality score, consistent with the purpose of a scoping review, which is to map the range, characteristics, and gaps of the evidence rather than to exclude studies according to methodological quality or estimate a pooled effect. These characteristics were used to qualify interpretation of the evidence but not to exclude studies or assign an overall quality rank.

Five dimensions were considered. First, speech task and elicitation context were characterized according to whether speech arose from sustained phonation, scripted reading or repetition, prompted or structured speech, semi-structured or free speech, naturalistic conversation, or passive or ambient capture. Second, outcome or reference-standard type was characterized according to whether the target construct was established using a structured or clinical diagnosis, a clinician-rated validated scale, a self-report validated scale, or an experimental or operational state definition; studies could contribute to more than one category when multiple reference standards were used. Third, validation strategy was characterized descriptively as association-only analysis, internal resampling or cross-validation, internal held-out testing, or independent and cross-dataset validation. Fourth, sample size and speaker role were considered as indicators of the breadth of the evidence base. Fifth, recording modality and elicitation context were examined as indicators of similarity to intended real-world use.

These dimensions were kept conceptually separate. Clinical interpretability addressed whether the feature could be understood; outcome and reference-standard characteristics and validation addressed the credibility and generalizability of the observed association; and recording context addressed the extent to which a feature had been evaluated under conditions resembling potential real-world deployment.

### 2.8 Data synthesis and presentation

Study characteristics and findings were summarized using descriptive statistics and narrative synthesis. Frequencies and percentages were calculated for publication period, study design, population type, mental health outcome, elicitation context, recording modality, biomarker domain, and clinical interpretability. Because studies could examine multiple outcomes or biomarker domains, these categories were not mutually exclusive.

The direction and consistency of associations were summarized narratively, with particular attention to clinically interpretable patterns. Predictive performance metrics were charted where reported but were not pooled. No meta-analysis was performed because of substantial heterogeneity in populations, outcomes, recording tasks, feature definitions, reference standards, and analytic approaches.

## 3 Results

### 3.1 Study selection

The database searches identified 1,524 records. After 132 duplicates were removed, 1,392 unique records underwent title and abstract screening. Of these, 1,303 were excluded, leaving 89 reports from database searching. One further report was identified through citation searching during peer review, giving 90 reports for full-text assessment. Twenty-two full-text reports were excluded because of an ineligible outcome (*n* = 12), intervention or concept (*n* = 1), study design (*n* = 3), non-English publication (*n* = 1), unavailable full text (*n* = 1), absence of an identifiable mental health outcome (*n* = 3), or absence of an identifiable voice or speech biomarker (*n* = 1). Sixty-eight studies met the inclusion criteria and were included in the review (Figure 1). The included studies are listed in references 9–76.

**Figure 1:**
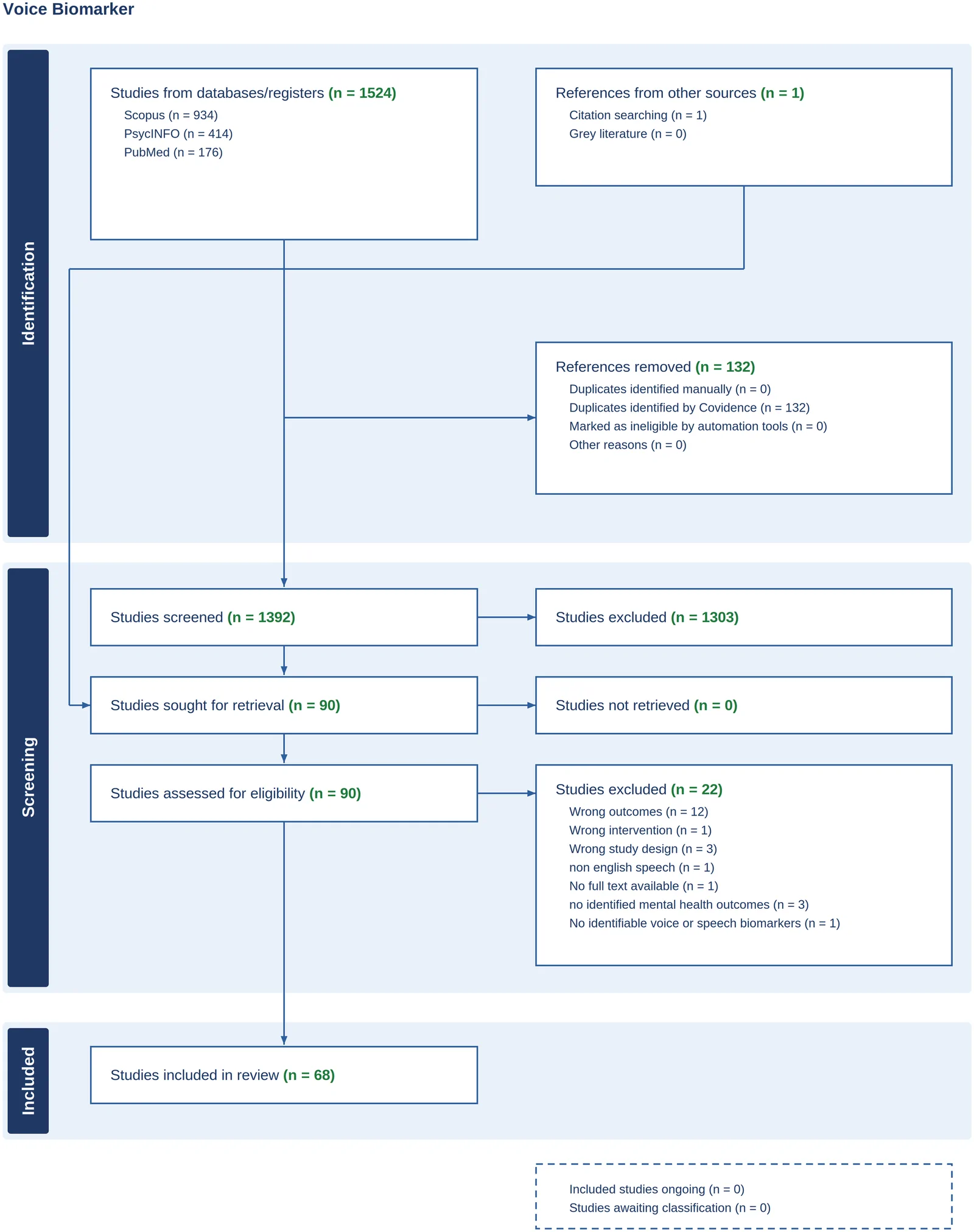
PRISMA-ScR flow diagram of study identification, screening, eligibility assessment and inclusion. Of 1,524 records identified through database searching, 89 reports were sought for retrieval; one further report was identified through citation searching, giving 90 reports assessed for eligibility and 68 included studies.

### 3.2 Characteristics of included studies

A total of 68 studies published between 2004 and 2026 were included (Table 2) (9–76) . Seven studies (10%) were published before 2015, 11 (16%) between 2015 and 2019, 15 (22%) between 2020 and 2022, and 35 (51%) between 2023 and 2026.

**Table 2:** Characteristics of the 68 included studies.

Characteristic	Category	n (of 68)	%
Publication year	2023–2026	35	51
	2020–2022	15	22
	2015–2019	11	16
	Before 2015	7	10
Country or region	China	13	19
	United States	11	16
	Germany	8	12
	Australia	5	7
	Multinational	4	6
	Canada	3	4
	Italy	3	4
	Japan	3	4
	Republic of Korea	3	4
	Brazil	2	3
	France	2	3
	Israel	2	3
	Other single-country settings^a^	9	13
Study design	Cross-sectional (incl. case-control)	47	69
	Longitudinal or prospective	15	22
	Experimental (induced fatigue)	3	4
	Secondary analysis of existing data	2	3
	Randomized controlled trial	1	1
Sample size	*N* < 50	15	22
	N 50–199	37	54
	N 200–999	10	15
	*N* ≥ 1,000	6	9
Speaker role	Patient and healthy control	35	51
	Patient only	18	26
	Healthy control only	14	21
	Dyadic (clinician and patient)	1	1
Elicitation context^b^	Controlled	54	79
	Naturalistic	16	24
	Passive or ambient	3	4
Recording modality^b^	Microphone	32	47
	Smartphone	23	34
	Other or unspecified	20	29
	Telephone	6	9
	Video (audio extracted)	3	4

a Czechia, Chile, Denmark, India, Netherlands, Poland, Spain, Türkiye and the United Kingdom each contributed one study. ^b^Studies using more than one elicitation context or recording modality contribute to multiple rows, so these categories sum to more than 68. Sample sizes ranged from 5 to 14,898 participants (median 104).

#### 3.2.1 Geographic distribution

Studies were conducted across 20 countries, with four further studies recruiting participants across multiple countries. China was the most frequently represented country (13 studies, 19%), followed by the United States (11, 16%), Germany (8, 12%), and Australia (5, 7%). Studies from Brazil, Chile, and India were uncommon in the dataset. Although all included publications were in English, several large cohorts involved non-English-speaking populations.

#### 3.2.2 Study design and sample size

Cross-sectional designs, including case-control and observational machine-learning classification studies, predominated (47/68, 69%). Longitudinal or prospective designs were used in 15 studies (22%). One randomized controlled trial assessed voice features as a secondary biomarker endpoint, three studies used experimental sleep-deprivation protocols, and two were secondary analyses of previously collected speech corpora.

Sample sizes ranged from 5 to 14,898 participants. Fifteen studies (22%) included fewer than 50 participants, 37 (54%) included 50–199, 10 (15%) included 200–999, and 6 (9%) included at least 1,000 participants. The median sample size was 104.

#### 3.2.3 Study populations and speaker roles

Eighteen studies (26%) recruited patients only, 14 (21%) recruited healthy controls or community volunteers only, and 35 (51%) included both patient and healthy control groups. Only one study recruited healthcare workers as the primary population: 89 French healthcare professionals assessed for perceived stress during the COVID-19 pandemic (9). One study analyzed speech from both clinician and patient within the same dyadic clinical interaction (10). No study recruited physicians or nurses specifically to examine burnout-related voice changes, and no study evaluated ambient voice monitoring during routine patient care.

### 3.3 Speech collection design and recording context

Controlled elicitation was used in 54 of 68 studies (79%), compared with naturalistic elicitation in 16 (24%) and passive or ambient capture in 3 (4%). The three passive or ambient studies comprised passive daily phone-call analysis in bipolar disorder (11, 12) and wrist-worn detection of speech during daily life in late-life depression (13).

Dedicated microphones were used in 32 studies (47%), smartphones in 23 (34%), telephone recordings in 6 (9%), and extracted video audio in 3 (4%); 20 studies (29%) used other or unspecified recording systems.

Speech-collection design materially influenced observed biomarker associations in the limited number of studies that compared tasks directly. Immel et al. found that a structured picture-description task predicted loneliness whereas free narrative storytelling in the same participants did not (14). Moore et al. reported that reading content itself introduced pitch variation, complicating interpretation of pitch as a mood-related feature (15). These direct comparisons demonstrate that a biomarker cannot be interpreted independently of the task from which it was derived; however, too few studies systematically compared the same biomarkers across multiple elicitation conditions to determine which interpretability tier is most stable across tasks.

### 3.4 Mental health outcomes

Depression was the primary or co-primary outcome in 56 of 68 studies (82%). Anxiety was examined in 9 studies, bipolar disorder or mania in 8, fatigue in 4, post-traumatic stress disorder in 3, and stress in 2. A further 10 studies examined other psychiatric or psychological outcomes, including suicidal risk, loneliness, schizophrenia-spectrum disorders, insomnia, and composite psychiatric outcomes not reported separately. Studies examining more than one outcome contributed to multiple categories (Table 3; Figure 2).

**Table 3:** Distribution of mental health outcomes across included studies.

Mental health outcome	Studies (n)	% of 68
Depression	56	82
Other mental health outcomes^a^	10	15
Anxiety	9	13
Bipolar disorder or mania	8	12
Fatigue	4	6
Post-traumatic stress disorder	3	4
Stress	2	3
Burnout or emotional exhaustion	0	0

a Suicidal risk, loneliness, schizophrenia-spectrum disorders, insomnia and composite psychiatric outcomes not reported separately. Studies examining more than one outcome contribute to multiple rows.

**Figure 2:**
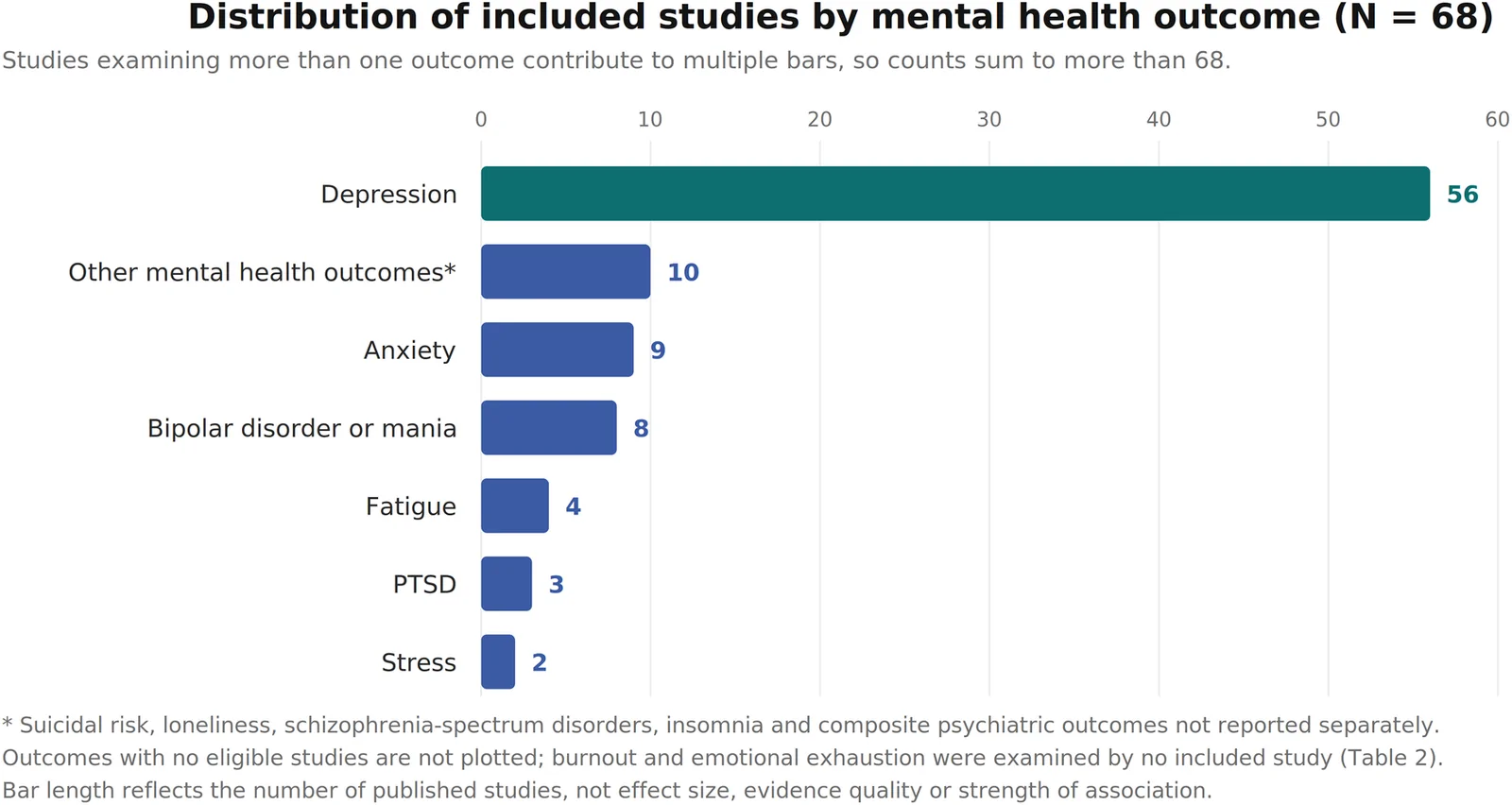
Distribution of included studies by mental health outcome. Studies examining more than one outcome contribute to multiple bars, so counts sum to more than 68. Outcomes with no eligible studies are not plotted (Table 3). Bar length reflects the volume of published research, not effect size, evidence quality or strength of association.

No included study examined burnout or emotional exhaustion as a primary or secondary outcome, and no study used the Maslach Burnout Inventory alongside voice analysis. The healthcare-worker study by König et al. assessed perceived stress using the Perceived Stress Scale and discussed relevance to burnout prevention, but did not measure burnout directly (9).

### 3.5 Outcome labels and reference standards

Outcome definition varied substantially across the evidence base. Mental health states were established using several different types of reference standard, including structured or clinician-established psychiatric diagnoses, clinician-rated symptom scales, self-report symptom measures, and experimentally or operationally defined states such as sleep deprivation or examination stress.

A DSM-IV or DSM-5 clinical diagnosis was used in 23 studies and an ICD-10 or ICD-11 diagnosis in 6; a structured or composite diagnostic interview (SCID, MINI, or CIDI) was used in 15. Among symptom scales, the Hamilton Depression Rating Scale was used in 19 studies, the Patient Health Questionnaire-9 or PHQ-8 in 18, the Beck Depression Inventory or BDI-II in 13, and the Montgomery-Åsberg Depression Rating Scale in 6. The Generalized Anxiety Disorder scale and the Young Mania Rating Scale were each used in 6 studies, the Hamilton Anxiety Rating Scale and the Quick Inventory of Depressive Symptomatology in 4 each, the PTSD Checklist or Clinician-Administered PTSD Scale in 3, the Stanford Sleepiness Scale in 2, and the Center for Epidemiologic Studies Depression Scale and the Perceived Stress Scale in 1 each. The Maslach Burnout Inventory was used in none (Table 4). Many studies used more than one type of measure.

**Table 4:** Outcome measurement instruments used across included studies.

Instrument	Type	Studies (n)
DSM-IV or DSM-5 clinical diagnosis	Diagnostic interview	23
Hamilton Depression Rating Scale (HAM-D/HDRS)	Clinician-rated	19
Patient Health Questionnaire (PHQ-9/PHQ-8)	Self-report	18
Structured or composite diagnostic interview (SCID, MINI, CIDI)	Diagnostic interview	15
Beck Depression Inventory (BDI/BDI-II)	Self-report	13
Montgomery-Åsberg Depression Rating Scale (MADRS)	Clinician-rated	6
ICD-10 or ICD-11 clinical diagnosis	Diagnostic interview	6
Generalized Anxiety Disorder scale (GAD-7)	Self-report	6
Young Mania Rating Scale (YMRS)	Clinician-rated	6
Hamilton Anxiety Rating Scale (HAM-A)	Clinician-rated	4
Quick Inventory of Depressive Symptomatology (QIDS)	Mixed	4
PTSD Checklist or Clinician-Administered PTSD Scale	Mixed	3
Stanford Sleepiness Scale	Self-report	2
Center for Epidemiologic Studies Depression Scale (CES-D)	Self-report	1
Perceived Stress Scale (PSS)	Self-report	1
Maslach Burnout Inventory (MBI)	Self-report	0

Studies using more than one instrument contribute to multiple rows.

These approaches were retained as separate methodological characteristics rather than treated as interchangeable evidence of biomarker validity. An association with a structured diagnostic interview addresses a different question from an association with self-reported symptom severity, while experimentally induced fatigue or stress represents a state manipulation rather than a psychiatric diagnosis. The heterogeneity of outcome definition was particularly relevant for depression, where studies variously used DSM- or ICD-based diagnoses, clinician-rated scales such as the HAM-D or MADRS, and self-report measures such as the PHQ-9 or BDI. Accordingly, agreement in the direction of a speech feature across studies should not be interpreted as replication of an identical clinical construct when different outcome definitions were used. Table 5 summarizes reference-standard type alongside sample size, speech-collection and validation characteristics; these categories are descriptive and are not intended as a quality ranking.

**Table 5:** Translational-methodological characteristics of the 68 included studies.

Domain	Category	n (of 68)	%
Sample size	*N* ≥ 100	35	51
	N 50–99	18	26
	*N* < 50	15	22
Reference standard	Diagnostic interview or clinician-rated scale	45	66
	Validated self-report scale only	20	29
	Other or not clearly reported	3	4
Validation strategy	Internal cross-validation or resampling only	60	88
	Internal held-out test partition	3	4
	Independent or cross-dataset validation	2	3
	No predictive model (descriptive or correlational)	3	4
Task standardization	Controlled protocol	49	72
	Naturalistic	11	16
	Mixed controlled and naturalistic	5	7
	Passive or ambient	3	4
Recording context	Laboratory or clinic recording	30	44
	Remote personal device	24	35
	Not clearly reported	11	16
	Ambient or wearable	3	4

Categories are descriptive and do not constitute a quality ranking; they were not used to weight, exclude or rank studies. Studies that did not report a separate held-out partition were classified as internal cross-validation or resampling.

### 3.6 Voice, speech, language, and communication biomarker domains

A wide range of biomarker domains were examined, and most studies reported more than one domain. Pitch was reported in 47 studies, vocal intensity or loudness in 38, MFCCs in 37, formant frequencies in 28, pause duration or frequency in 27, voice quality in 27, jitter in 24, verbal output in 23, shimmer in 23, harmonics-to-noise ratio in 23, prosody or rhythm in 21, speech rate in 19, word or pronoun use in 9, sentiment or emotional tone in 8, response latency in 4, and communication behaviour in 2. A further 56 studies reported other technical features, including deep-learning embeddings and i-vectors. The distribution of biomarker domains across mental health outcomes is shown in Figure 3 and summarized in Table 1.

**Figure 3:**
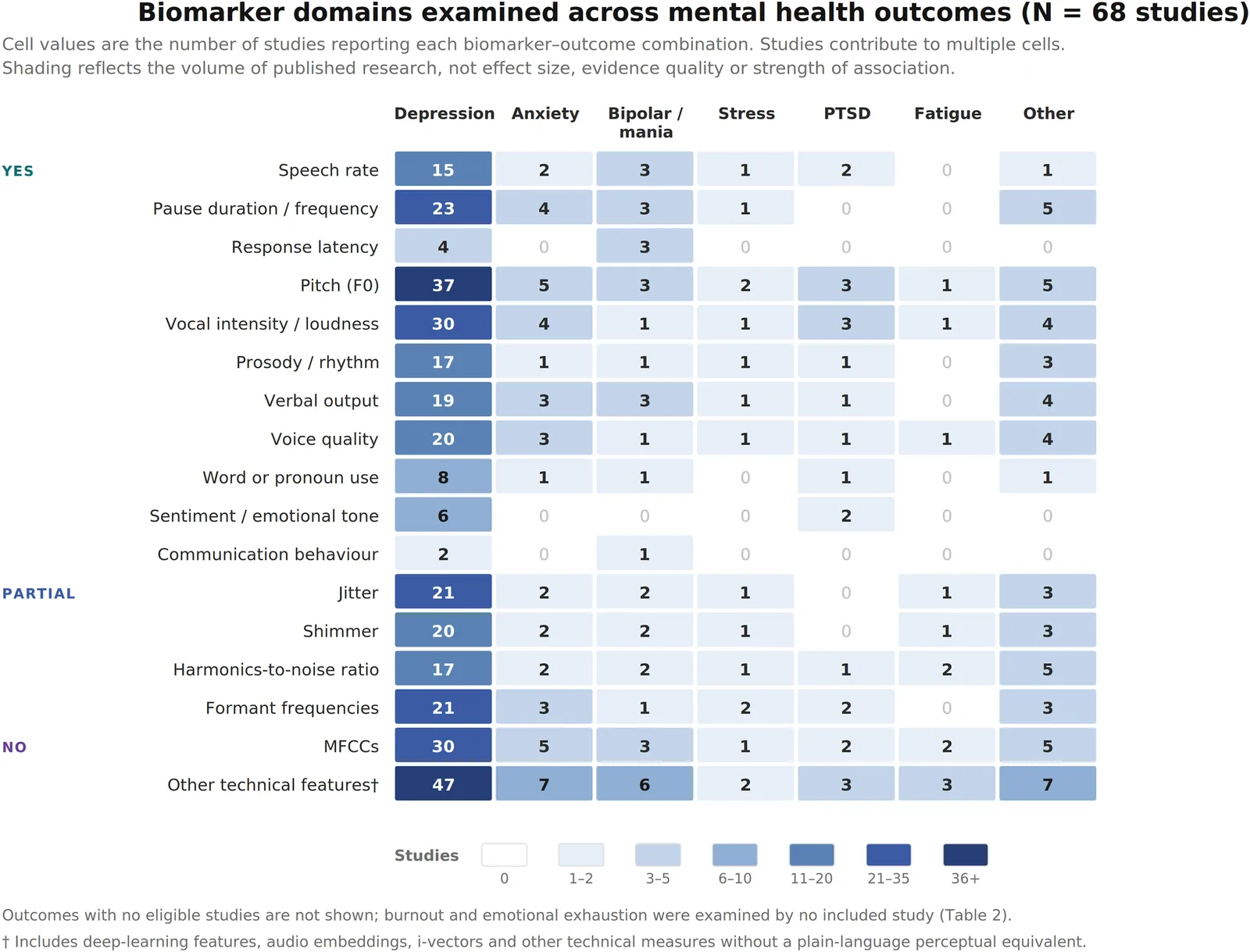
Heat map of biomarker domains examined across mental health outcomes. Cell values represent the number of studies reporting each biomarker–outcome combination, grouped by clinical interpretability tier. Studies examining multiple biomarker domains or outcomes could contribute to more than one cell. Outcomes with no eligible studies are not shown. Shading reflects the volume of literature rather than evidence quality, effect size, or strength of association. Depression contained the largest number of studies across almost every biomarker domain.

#### 3.6.1 Clinical interpretability

The three-tier interpretability framework is shown in Figure 4. At study level, 29 studies (43%) reported at least one directly interpretable marker as their principal feature class, 26 (38%) a partially interpretable marker, and 13 (19%) technical-only markers.

**Figure 4:**
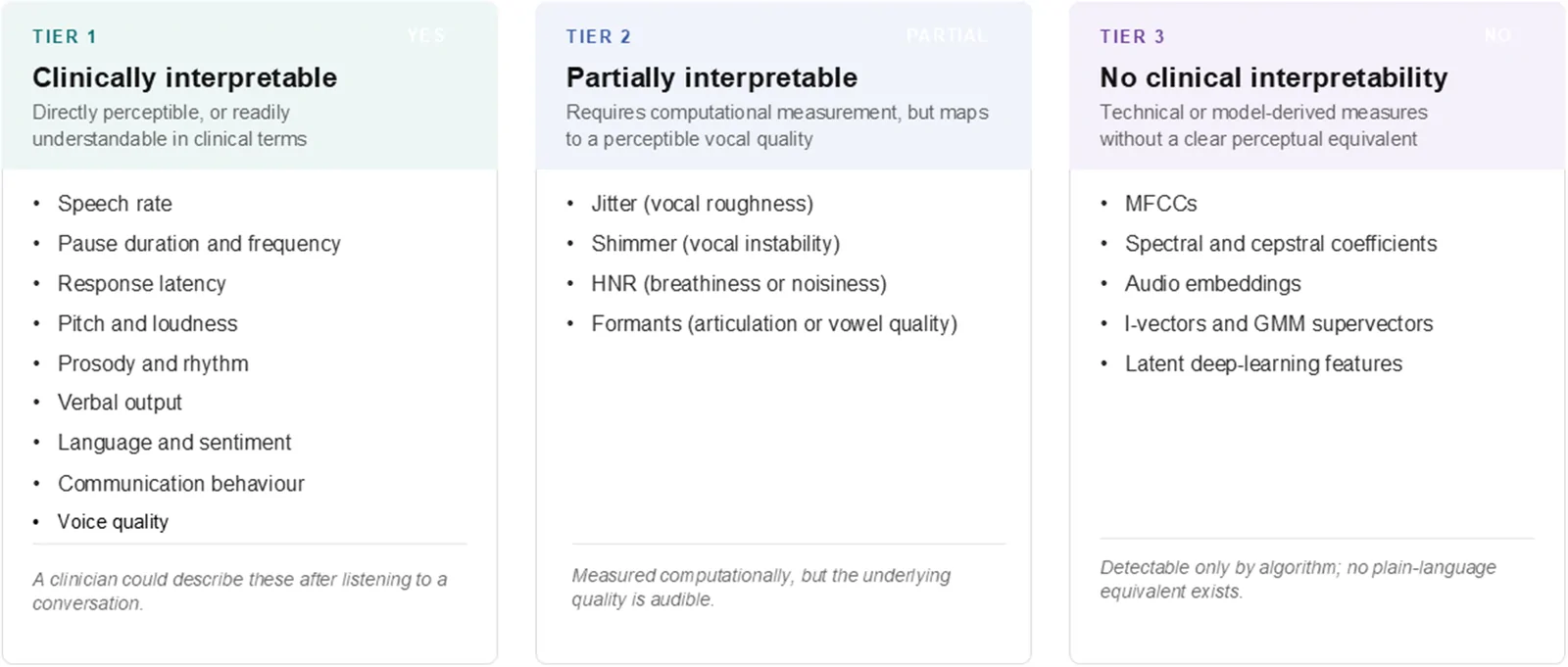
Three-tier framework classifying voice and speech biomarkers by clinical interpretability. Tier 1 includes directly interpretable markers such as speech rate, pauses, pitch, loudness, prosody, verbal output, language, sentiment, and communication behaviour. Tier 2 includes jitter, shimmer, harmonics-to-noise ratio, and formant frequencies. Tier 3 includes MFCCs, spectral or cepstral coefficients, audio embeddings, i-vectors, and latent deep-learning features. Interpretability is one dimension of translational readiness and does not imply validity, evidence quality, predictive performance, or deployability.

Within individual studies, higher predictive performance did not consistently align with greater clinical interpretability. Moore et al. reported stronger depression clustering with glottal-spectrum features than with pitch and speaking rate (15). Gao et al. found no individually significant differences across several interpretable fatigue-related features, whereas a multi-feature machine-learning model achieved 94% accuracy in their small experimental sample (16). In contrast, Cummins et al. reported replication of speaking-rate and vocal-intensity associations with depression severity across languages and countries (17). Direct evidence comparing the stability of different interpretability tiers across speech tasks was sparse. Accordingly, the review can describe where different feature types have been studied but cannot conclude that directly interpretable features are intrinsically more robust to changes in elicitation context than partially interpretable or technical-only features.

### 3.7 Findings by mental health outcome

#### 3.7.1 Depression

Across the 56 studies examining depression, clinically interpretable findings most often involved slower speech, longer or more frequent pauses, reduced vocal intensity, lower or less variable pitch, flatter prosody, and reduced verbal output. These directional patterns were reported across multiple countries and recording modalities, although not uniformly. Technical-only features, particularly MFCCs, often contributed to high-performing classifiers within individual datasets but did not show a consistent directional interpretation across coefficients.

Several studies illustrated the range of performance and feature types. Taguchi et al. reported an AUC of 0.88 for a discriminating MFCC coefficient in a Japanese sample (18). Afshan et al. reported 94.8% accuracy using voice-quality features combined with MFCC-derived i-vectors in a large Mandarin-speaking cohort (19), whereas Di et al. reported better performance for MFCC i-vectors than raw MFCCs in another large cohort (20). Vilenchik et al. reported that a voice-only model achieved a modest accuracy gain over baseline in an unselected clinical sample, with performance rising substantially only after the sample was restricted to participants at the extremes of a self-reported distress scale, and showed using speech embeddings that the dominant variance in their data reflected speaker sex and identity rather than depression (21). Linguistic features also contributed useful signal: Maran et al. reported stronger performance for a linguistic model than for acoustic features alone (22), and Wiseman et al. reported an AUC of 0.90 for a composite incorporating speech timing, language valence, emotional intensity, and agency (23).

Longitudinal evidence was less common. Lin et al. observed changes in multiple acoustic features over antidepressant treatment (24). Mundt et al. found that pause and timing measures tracked treatment response in a randomized trial (25). Wadle et al. and Hartnagel et al., analyzing repeated within-person assessments from the same small dataset, showed that momentary voice features could track within-person depression severity but generalized poorly to unseen participants (26, 27).

#### 3.7.2 Anxiety

Nine studies examined anxiety as a primary or co-primary outcome. Ciampelli et al. reported that adolescent acoustic-prosodic features predicted later social anxiety disorder, with different feature importance by sex (28). Teferra et al. identified statistically significant but small associations between anxiety severity and speaking duration, shimmer, and negative emotional language in a large online sample (29). Cohen et al. reported moderate classification performance from virtual-agent interview speech (30). Across the smaller anxiety literature, the direction of speech changes was less consistent than for depression.

#### 3.7.3 Bipolar disorder and mania

Eight studies included bipolar disorder or mania. Aldeneh et al. was distinctive in analyzing both patient and clinician speech during clinical telephone interviews; dialogue features predicted mood episodes, and clinician speaking behaviour also changed according to patient mood state (10). Kaczmarek-Majer et al. analyzed routine phone calls over a mean of 208 days and identified sex-dependent associations between acoustic features and manic state (12). Pan et al. found that voice-derived features could distinguish depression from schizophrenia but performed poorly in separating depression from euthymic bipolar disorder, illustrating a challenge for differential diagnostic applications (31).

#### 3.7.4 Fatigue and stress

Four studies examined fatigue. Greeley et al. reported strong correlations between selected speech features and operational fatigue in aviation settings (32). Gao et al. found that a multifeature model outperformed individual interpretable features following sleep deprivation (16), while Gaur et al. identified changes in harmonics-to-noise ratio during overnight fatigue (33). Riad et al. found lower discrimination for fatigue than for depression or anxiety using speech embeddings (34).

Two studies examined stress. König et al., the only healthcare-worker study in the review, used brief telephone speech samples to model Perceived Stress Scale scores and found task- and sex-dependent feature associations (9). Sigmund reported increased mean fundamental frequency and altered vowel spectra during examination stress in male students (35).

#### 3.7.5 Post-traumatic stress disorder and suicidal risk

Three studies examined post-traumatic stress disorder. Marmar et al. reported an acoustic model with an AUC of 0.954 in male veterans, incorporating features such as slower articulation, reduced tonal variation, and flatter energy patterns (36). Quatieri et al. reported improved screening performance when emotionally selected speech segments were analyzed rather than the full recording (37), and Menne et al. identified substantial sex differences in PTSD-related voice features (38). Three studies examined suicidal risk or suicidality as an outcome or co-outcome (30, 39, 40).

### 3.8 Validation and generalizability

Validation strategy represented a major limitation of the evidence base. Only 2 of 68 studies (3%) reported validation on an independent or cross-dataset sample: one applied a previously developed model to a new population and one tested timing features on an external dataset. A further 3 studies (4%) reported performance on an internal held-out test partition drawn from the same collection. Sixty studies (88%) reported internal cross-validation or resampling alone, most commonly k-fold, leave-one-out, or repeated random splits, and 3 (4%) reported no predictive model (Table 5). Because an internal held-out partition shares the recording conditions, population, and labelling procedure of the training data, it does not establish transportability; high classification performance within development datasets should therefore not be interpreted as evidence that a model will perform in a new population or recording environment.

Personalization also emerged as an important issue. Hartnagel et al. reported that within-patient models predicted depression severity better than models applied to unseen participants, where performance approached chance (26). Medication status was inconsistently reported or controlled in clinical samples, and psychotropic treatment could plausibly affect motor, arousal, or cognitive features relevant to speech. Sex distribution was frequently unbalanced, and several studies reported sex-specific or directionally different associations. Large cohorts were also concentrated in selected populations, limiting generalizability across languages, cultures, ages, and clinical contexts.

## 4 Discussion

### 4.1 Principal findings

The principal contribution of this scoping review is not the identification of a single optimal voice biomarker, but the mapping of voice-based mental health evidence through a translational-methodological lens. Viewed through the three dimensions defined above, the evidence base is considerably less mature than classification performance alone might suggest. Voice and speech markers have been studied extensively, particularly in depression (56 of 68 included studies), and several clinically recognizable patterns recur across studies. However, much of the evidence derives from controlled elicitation (79% of all studies), heterogeneous reference standards, cross-sectional samples, and internal validation. The central translational gap is therefore not whether speech contains information related to mental health, but whether specific signals retain their meaning, validity, and predictive performance when measured across people, tasks, languages, recording conditions, and real-world conversational settings.

### 4.2 Clinical interpretability as a translational dimension

Clinical interpretability is valuable for transparency but should not be conflated with validity or clinical utility. A feature such as slower speech may be readily understood by a clinician, but its usefulness as a digital biomarker depends on whether the association is anchored to a well-defined target construct, replicates across populations and tasks, and remains measurable in the intended setting. Conversely, a technical feature such as an MFCC or learned embedding may have little intuitive meaning but could still be clinically useful if its performance is robustly and externally validated. The three-tier framework should therefore be understood as an interpretability framework rather than an evidence-quality hierarchy.

Interpretability should also not be assumed to imply superior predictive performance. Several included studies showed that multifeature or technical representations outperformed individual observable features, while other studies demonstrated reproducible associations for simple timing and intensity measures. Future systems may therefore benefit from hybrid approaches in which technically rich models are paired with clinically meaningful summaries rather than forcing a choice between performance and explainability.

### 4.3 Data-collection design, label validity, and deployability

Data-collection design is part of the interpretation of a voice biomarker rather than merely a technical detail. Standardized reading, sustained phonation, prompted narratives, clinical interviews, and spontaneous conversation impose different linguistic, cognitive, motor, and interactional demands. A feature associated with depression during a fixed reading task cannot automatically be assumed to have the same meaning or performance during spontaneous dialogue. The predominance of controlled speech tasks is a central limitation for real-world digital phenotyping.

Outcome definition creates a parallel problem. Voice features were evaluated against structured diagnoses, clinician-rated severity measures, self-report questionnaires, and experimentally defined states. These reference standards answer related but different questions. Classification of DSM-defined major depressive disorder is not equivalent to estimating PHQ-9 symptom burden, and neither is equivalent to identifying momentary distress. Translational interpretation therefore requires attention to both the speech protocol and the construct against which the biomarker was evaluated.

Finally, technical extractability should not be equated with deployability. Speech rate, pauses, pitch, loudness, prosody, verbal output, linguistic features, and turn-taking can in principle be calculated from conversational audio, but this review does not establish that associations observed under controlled conditions remain valid in spontaneous multispeaker encounters. Real-world deployment introduces speaker overlap, variable microphone distance, environmental noise, topic effects, language variation, interpersonal adaptation, and other sources of variance that are minimized in many research datasets.

### 4.4 Implications for occupational and ambient applications

Occupational applications remain largely untested within the current evidence base. The included literature provides candidate speech and language features associated with mental health and psychological states, but evidence derived from psychiatric or general populations should not be treated as a proxy for occupational burnout or professional well-being. Overlapping symptoms among depression, stress, fatigue, and burnout do not establish that their speech signatures are equivalent.

Ambient clinical technologies nevertheless provide a potential research environment in which this question could be tested prospectively. Such studies would require validated occupational outcomes, repeated within-person measurement, reliable speaker separation, characterization of the real-world audio conditions described in Section 4.3, external validation, and explicit governance for secondary use of clinical audio. The current review therefore provides methodological hypotheses for future study rather than evidence supporting passive assessment of clinician burnout.

### 4.5 Strengths and limitations

This review has several strengths. It included 68 empirical studies across multiple mental health outcomes and populations, retained both interpretable and technical biomarkers rather than excluding one part of the field, and separately mapped elicitation context, recording modality, speaker role, and interpretability. Independent screening and data charting by two reviewers and use of JBI and PRISMA-ScR guidance strengthened methodological transparency.

Several limitations should also be considered. The search was limited to PubMed, PsycINFO, and Scopus and to English-language publications; technical studies indexed only in engineering-specific databases may therefore have been missed. One eligible study was identified through citation searching after the original search, indicating that search sensitivity was imperfect. The evidence base was dominated by depression, limiting comparison across other mental health states. No formal risk-of-bias appraisal was performed, which is consistent with the mapping purpose of a scoping review but means that study counts should not be interpreted as evidence strength. The clinical interpretability framework was developed for this review and has not been independently validated, and the translational-methodological characterization was descriptive rather than a validated instrument. Categorization of speech task, reference standard, and validation strategy necessarily simplifies heterogeneous study designs, and some studies contributed to more than one category. The review also lacked sufficient direct within-study comparisons to determine whether one interpretability tier is intrinsically more robust across elicitation contexts. Finally, biomarker definitions and outcome measures were heterogeneous, and many studies reported several correlated features.

### 4.6 Priorities for future research

Future work should move from cross-sectional classification toward longitudinal validation in real-world settings. Priorities include naturalistic conversational datasets with standardized reporting of recording conditions; repeated within-person measurements linked to validated clinical or psychological outcomes; external validation across institutions, languages, sex and gender groups, ages, and cultural contexts; explicit comparison of interpretable, technical, and hybrid models; evaluation of confounding from medication, sleep, physical illness, and environmental acoustics; and prospective governance frameworks for passive or ambient capture. For occupational applications, clinician-specific studies should test burnout and professional fulfillment directly rather than infer them from depression, anxiety, or stress.

## 5 Conclusion

Voice and speech contain measurable acoustic, linguistic, and interactional signals associated with mental health, particularly depression. Translation of these signals into clinically useful digital biomarkers requires more than predictive performance within individual datasets: interpretability, validity, and deployability are distinct requirements, and the current evidence base is dominated by controlled elicitation, heterogeneous outcome definitions, and internal validation.

Clinically interpretable features such as speech timing, pausing, pitch, vocal intensity, prosody, verbal output, and conversational behaviour may offer advantages for transparency, while technical representations may contribute predictive information that is less readily explainable. Neither feature class should be considered clinically useful without appropriate validation. Future studies should evaluate candidate biomarkers using clearly defined clinical or psychological reference standards, compare performance across speech tasks and recording contexts, prioritize longitudinal and external validation, and test models in diverse naturalistic populations. Existing research provides a foundation for translational investigation, but not yet evidence supporting passive or ambient mental health assessment in routine clinical practice.

## Data Availability

The datasets presented in this study can be found in online repositories. The names of the repository/repositories and accession number(s) can be found in the article/supplementary material.
