# Supplementary material for: Voice and speech as digital biomarkers of mental health: a scoping review of clinical interpretability, data collection and translational readiness
Source: Front Digit Health. 2026 Oct 5;8:1964389. doi: 10.3389/fdgth.2026.1964389 (PMC13677924; doi:10.3389/fdgth.2026.1964389)
Supplement: Supplementary file 1 [file Datasheet1.pdf]

# Data Extraction Sheet — 68 Included Studies

Voice and Speech as Digital Biomarkers of Mental Health: A Scoping Review of Clinical Interpretability, Data Collection and Translational Readiness. One record per included study.

## Study 1 | Abutara et al. (2026)

| | |
|---|---|
| **Study design** | Cross-sectional observational study |
| **Setting** | Pre-university preparatory courses in Brazil |
| **Population Type (adult, student, specific occupational groups)** | 62 Brazilian Portuguese-speaking adults exposed to academic stress |
| **Speaker Role** | Patient |
| **Elicitation Context** | Naturalistic |
| **Recording Modality** | Smartphone |
| **Sample size** | 62 |
| **Mental health outcome** | Depression, Anxiety |
| **Outcome Biomarker Domain** | MFCCs, Other |
| **Specific variables of marker studied** | MFCC1, MFCC11, spectral entropy, spectral rolloff, delta chroma features |
| **clinical interpretability** | No |
| **Perceptial Label (if yes or Partial)** | |
| **Outcome Measure/Tool?** | PHQ-9, GAD-7 |
| **Direction of Association** | Higher depression was linked to reduced cepstral and chroma dynamics; higher anxiety was linked to greater spectral entropy and tonal modulation |
| **Effect Size/Model Performance/Statistical Significance** | Depression: adjusted $R^2$=0.366, AUC=0.839, CV AUC=0.687. Anxiety: adjusted $R^2$=0.230, AUC=0.900, CV AUC=0.694 |
| **Main Author Conclusions** | Spontaneous speech features were associated with depression and anxiety, but findings were exploratory and not clinically validated |
| **Key Limitations** | Small sample, cross-sectional design, many predictors, uncontrolled recording conditions, no external validation |
| **Notes** | Open-ended prompted recordings were coded Naturalistic. Abstract and tables reported inconsistent $R^2$ values; depression model text and Table 4 also differed on spectral entropy versus spectral rolloff. |

## Study 2 | Afshan et al. (2018)

| | |
|---|---|
| **Study design** | Machine-learning classification study |
| **Setting** | CONVERGE study, China |

| | |
|---|---|
| **Population Type (adult, student, specific occupational groups)** | 1688 Mandarin-speaking women, including 735 with depression and 953 healthy controls |
| **Speaker Role** | Patient, Healthy Control |
| **Elicitation Context** | Controlled |
| **Recording Modality** | Other |
| **Sample size** | 1688 |
| **Mental health outcome** | Depression |
| **Outcome Biomarker Domain** | Pitch, Voice quality, Harmonics-to-noise ratio, Formant frequencies, MFCCs, Other |
| **Specific variables of marker studied** | F0, formants F1–F3, harmonic amplitude differences, formant amplitudes, CPP, MFCCs, ComParE16 features, i-vectors |
| **clinical interpretability** | Partial |
| **Perceptial Label (if yes or Partial)** | Pitch, vowel quality, breathiness, roughness, and overall voice quality |
| **Outcome Measure/Tool?** | Chinese Composite International Diagnostic Interview using DSM-IV criteria |
| **Direction of Association** | Individual marker directions were not reported; combining voice-quality and MFCC features improved depression classification |
| **Effect Size/Model Performance/Statistical Significance** | Best fused i-vector model: accuracy 94.8%, F1 0.951, precision 0.926, recall 0.977; accuracy remained 77% with 10-second recordings |
| **Main Author Conclusions** | Voice-quality features complemented MFCCs and improved automated depression detection |
| **Key Limitations** | Women only, Mandarin only, no external validation, single train-test split, recording conditions not reported |
| **Notes** | Clinical interpretability was rated Partial because voice-quality features have perceptual correlates, while MFCCs and i-vectors are technical. |

## Study 3 | Aldeneh et al. (2019)

| | |
|---|---|
| **Study design** | Longitudinal observational and machine-learning study |
| **Setting** | Weekly clinician–patient assessment calls from the PRIORI bipolar disorder study |
| **Population Type (adult, student, specific occupational groups)** | 47 adults with bipolar I or II disorder and 9 clinicians |
| **Speaker Role** | Mixed/Dyadic (2 person conversation) |
| **Elicitation Context** | Controlled |
| **Recording Modality** | Smartphone, Telephone |
| **Sample size** | 47 patients, 9 clinicians, 641 calls |
| **Mental health outcome** | Depression, Bipolar/Mania |
| **Outcome Biomarker Domain** | Pause duration/frequency, Prosody/rhythm, Response latency, Verbal output, Communication behaviour |

| | |
|---|---|
| **Specific variables of marker studied** | Floor-control ratio, turn lengths, within-turn pauses, response latency, consecutive turns, turn switches, speech rhythm |
| **clinical interpretability** | Yes |
| **Perceptial Label (if yes or Partial)** | More or less talking, longer pauses, slower responses, longer turns, and altered turn-taking |
| **Outcome Measure/Tool?** | Hamilton Depression Rating Scale, Young Mania Rating Scale |
| **Direction of Association** | Depression involved more patient floor control, more long pauses and greater response-time variability; mania involved more patient talk time and longer turns |
| **Effect Size/Model Performance/Statistical Significance** | Depression: floor control +2.66%, response-latency variability +63.8 ms; best fused AUROC 0.764. Mania: floor control +8.28%, turn length +313 ms; best AUROC 0.676 with rhythm alone |
| **Main Author Conclusions** | Dialogue patterns changed across mood episodes and improved depression detection when combined with rhythm features |
| **Key Limitations** | Small sample, few manic calls, clinician analysis mostly female clinicians, selected mood-state thresholds, no external validation |
| **Notes** | Dialogue features improved depression but not mania classification, likely because only 56 manic calls were available. |

## Study 4 | Alghowinem et al. (2012)

| | |
|---|---|
| **Study design** | Case-control classification study |
| **Setting** | Black Dog Institute clinical interviews, Sydney, Australia |
| **Population Type (adult, student, specific occupational groups)** | 30 adults with clinical depression and 30 age- and gender-matched healthy controls |
| **Speaker Role** | Patient, Healthy Control |
| **Elicitation Context** | Controlled |
| **Recording Modality** | Microphone |
| **Sample size** | 60 |
| **Mental health outcome** | Depression |
| **Outcome Biomarker Domain** | Speech rate, Pause duration/frequency, Pitch, Vocal intensity/loudness, Response latency, Verbal output, Shimmer, Harmonics-to-noise ratio, Formant frequencies, MFCCs, Communication behaviour |
| **Specific variables of marker studied** | MFCCs, energy, loudness, pitch, shimmer, HNR, response time, syllable duration, articulation rate, laughter and speech overlap |
| **clinical interpretability** | Yes |
| **Perceptial Label (if yes or Partial)** | Slower speech, longer response delays, less laughter and less conversational involvement |
| **Outcome Measure/Tool?** | DSM-IV diagnostic interview |
| **Direction of Association** | Depressed participants had longer first-response times and syllables, lower articulation rates, less laughter and less overlapping speech; acoustic performance varied by gender |
| **Effect Size/Model Performance/Statistical Significance** | Best mixed-gender acoustic WAR=70%; female WAR=77% for shimmer/log energy; male WAR=80% for root mean energy; first-response time $p=0.013$, syllable duration $p=0.036$, overlap $p<0.001$ |

| | |
|---|---|
| **Main Author Conclusions** | Spontaneous speech and interaction features distinguished depressed from healthy participants, particularly during positive-emotion questions |
| **Key Limitations** | Small sample, exploratory analyses, emotion elicitation not validated, no external validation |
| **Notes** | Depression recognition was generally stronger in women; positive-emotion speech was more discriminative than negative-emotion speech. |

## Study 5 | Alghowinem et al. (2013)

| | |
|---|---|
| **Study design** | Case-control machine-learning classification study |
| **Setting** | Black Dog Institute clinical interviews, Sydney, Australia |
| **Population Type (adult, student, specific occupational groups)** | 30 adults with severe depression and 30 gender-matched healthy controls |
| **Speaker Role** | Patient, Healthy Control |
| **Elicitation Context** | Controlled |
| **Recording Modality** | Microphone |
| **Sample size** | 60 |
| **Mental health outcome** | Depression |
| **Outcome Biomarker Domain** | Pitch, Vocal intensity/loudness, Voice quality, Jitter, Shimmer, Harmonics-to-noise ratio, Formant frequencies, MFCCs |
| **Specific variables of marker studied** | F0, voicing probability, MFCCs, log energy, RMS energy, intensity, loudness, formants, jitter, shimmer, HNR |
| **clinical interpretability** | Yes |
| **Perceptial Label (if yes or Partial)** | Quieter, lower-energy and less intense speech |
| **Outcome Measure/Tool?** | Hamilton Depression Rating Scale >15 |
| **Direction of Association** | Loudness, RMS energy and intensity were the strongest features; the hybrid GMM-SVM classifier performed best |
| **Effect Size/Model Performance/Statistical Significance** | Best single feature: loudness recall 85.0%; best overall model: GMM-SVM decision fusion recall 91.7% |
| **Main Author Conclusions** | Loudness and energy-related features were most useful for detecting depression, and hybrid classification outperformed the other models |
| **Key Limitations** | Small sample, fixed 92-second speech segments, no external validation, single-site English-speaking sample |
| **Notes** | Feature fusion performed worse than score or decision fusion; findings overlap with the authors' earlier Black Dog Institute study. |

## Study 6 | Alghowinem et al. (2013)

| | |
|---|---|
| **Study design** | Case-control classification study |
| **Setting** | Black Dog Institute clinical interviews, Sydney, Australia |
| **Population Type (adult, student, specific occupational groups)** | 30 adults with depression and 30 gender-matched healthy controls |

| | |
|---|---|
| **Speaker Role** | Patient, Healthy Control |
| **Elicitation Context** | Controlled |
| **Recording Modality** | Microphone |
| **Sample size** | 60 |
| **Mental health outcome** | Depression |
| **Outcome Biomarker Domain** | Pitch, Vocal intensity/loudness, Voice quality, Jitter, Shimmer, Harmonics-to-noise ratio, Other |
| **Specific variables of marker studied** | F0, intensity, loudness, voicing probability, voice quality, jitter, shimmer, HNR, log and RMS energy, Teager energy features |
| **clinical interpretability** | Yes |
| **Perceptial Label (if yes or Partial)** | Quieter, lower-energy, less variable and more monotone speech |
| **Outcome Measure/Tool?** | Clinical diagnosis of depression; exact diagnostic instrument not reported |
| **Direction of Association** | Depressed participants had lower intensity, loudness, energy and voice-quality values; most jitter, shimmer and HNR differences were not significant |
| **Effect Size/Model Performance/Statistical Significance** | Best accuracy was 80% using RMS Teager energy in mixed speech or log Teager energy in unvoiced speech; best linear features reached 75% |
| **Main Author Conclusions** | Mixed and unvoiced speech contained useful depression cues, and energy-related features were the strongest classifiers |
| **Key Limitations** | Small sample, only 20 short utterances per participant, no external validation, diagnostic details limited |
| **Notes** | This appears to use the same Black Dog Institute cohort as the authors' related 2012–2013 studies; overlap should be considered when summarizing study counts. |

## Study 7 | Cansel et al. (2023)

| | |
|---|---|
| **Study design** | Cross-sectional machine-learning classification study |
| **Setting** | Psychiatry outpatient clinic at Inonu University, Turkey |
| **Population Type (adult, student, specific occupational groups)** | 79 psychiatric patients and 25 healthy controls; schizophrenia spectrum, anxiety, depressive and bipolar disorders |
| **Speaker Role** | Patient, Healthy Control |
| **Elicitation Context** | Controlled |
| **Recording Modality** | Smartphone |
| **Sample size** | 104 |
| **Mental health outcome** | Depression, Anxiety, Bipolar/Mania, Other |
| **Outcome Biomarker Domain** | MFCCs, Other |
| **Specific variables of marker studied** | MFCCs, GTCCs, wavelet packet coefficients |
| **clinical interpretability** | No |

| | |
|---|---|
| **Perceptial Label (if yes or Partial)** | |
| **Outcome Measure/Tool?** | DSM-5 diagnosis, HAM-D, HAM-A, YMRS, SANS, SAPS |
| **Direction of Association** | Individual feature directions were not reported; cepstral features distinguished four psychiatric disorder groups and healthy controls |
| **Effect Size/Model Performance/Statistical Significance** | Best kNN model: accuracy 96.9%, sensitivity 96.9%, specificity 99.2%, F1 0.969; SVM accuracy 96.5% |
| **Main Author Conclusions** | Speech-based cepstral features showed high performance in classifying major psychiatric disorders and healthy controls |
| **Key Limitations** | Small single-region sample, data augmentation from the same recordings, no external validation, potential medication and demographic confounding, no subgroup analysis |
| **Notes** | Participants read two neutral texts. Depression was the weakest class for kNN, while bipolar disorder was the weakest for SVM. |

## Study 8 | Ciampelli et al. (2025)

| | |
|---|---|
| **Study design** | Longitudinal cohort and machine-learning study |
| **Setting** | TRAILS population cohort, Netherlands |
| **Population Type (adult, student, specific occupational groups)** | 581 adolescents aged 15–17 years at baseline, followed to age 18–20 |
| **Speaker Role** | Patient, Healthy Control |
| **Elicitation Context** | Controlled |
| **Recording Modality** | Microphone, Video |
| **Sample size** | 581 |
| **Mental health outcome** | Anxiety |
| **Outcome Biomarker Domain** | Speech rate, Pause duration/frequency, Pitch, Vocal intensity/loudness, Voice quality, Formant frequencies, MFCCs |
| **Specific variables of marker studied** | Pitch slope and range, loudness slope and peaks, F1, MFCC variability, voiced segments and pause-related features |
| **clinical interpretability** | Partial |
| **Perceptial Label (if yes or Partial)** | Less pitch variation, altered loudness, faster speech and changes in pausing or voice quality |
| **Outcome Measure/Tool?** | Youth Self Report, Adult Self Report, CIDI 3.0 DSM-IV anxiety diagnoses |
| **Direction of Association** | Higher anxiety was linked to reduced pitch dynamism in both sexes; males also showed narrower pitch range and lower F1, while future anxiety was associated with loudness instability in males and faster speech or altered voice quality in females |
| **Effect Size/Model Performance/Statistical Significance** | SAD prediction: male AUC 85.4%, accuracy 73.5%; female AUC 73.9%, accuracy 67.7%. Acoustic features added 5.4% and 10.9% explained variance in males and females |
| **Main Author Conclusions** | Acoustic markers during social stress predicted later anxiety, especially social anxiety disorder in male adolescents |
| **Key Limitations** | Cross-sectional findings did not survive multiple-testing correction, sex-stratified subtype samples were small, lifetime diagnoses may have preceded speech recording, possible unmeasured comorbidity |

| | |
|---|---|
| **Notes** | Social anxiety was predicted better than overall anxiety, likely because the speech task specifically induced social-evaluative stress. |

## Study 9 | Cohen et al. (2023)

| | |
|---|---|
| **Study design** | Case-control feasibility and machine-learning study |
| **Setting** | Remote virtual-agent interviews conducted in the United States |
| **Population Type (adult, student, specific occupational groups)** | 68 English-speaking adults recruited for depression, anxiety and suicide-risk research |
| **Speaker Role** | Patient |
| **Elicitation Context** | Controlled |
| **Recording Modality** | Microphone, Video |
| **Sample size** | 68 participants, 73 sessions |
| **Mental health outcome** | Depression, Anxiety, Other |
| **Outcome Biomarker Domain** | Pause duration/frequency, Pitch, Vocal intensity/loudness, Voice quality, Jitter, Shimmer, Harmonics-to-noise ratio, Verbal output |
| **Specific variables of marker studied** | Percent pause time, F0 variability, jitter, shimmer, HNR, speaking duration and articulation duration |
| **clinical interpretability** | Yes |
| **Perceptial Label (if yes or Partial)** | More pausing, flatter pitch and altered voice quality |
| **Outcome Measure/Tool?** | PHQ-9, GAD-7, Columbia-Suicide Severity Rating Scale |
| **Direction of Association** | Depression and suicide risk were associated with more pause time; depression also showed lower pitch variability, jitter and shimmer, while anxiety showed higher HNR |
| **Effect Size/Model Performance/Statistical Significance** | Best models: depression AUC 0.70, anxiety AUC 0.71, suicide risk AUC 0.76; depression pause time correlated with PHQ-9 scores, r=0.268 |
| **Main Author Conclusions** | Combining speech, facial and language features improved identification of depression, anxiety and suicide risk |
| **Key Limitations** | Small convenience sample, repeated sessions from some participants, screening rather than diagnostic labels, no external validation, several models near chance |
| **Notes** | Suicide risk was coded Other because it is not a dedicated dropdown option; the interview included structured open-ended questions about hope, fear, anger, secrets and emotional pain. |

## Study 10 | Crocamo et al. (2025)

| | |
|---|---|
| **Study design** | Pilot cross-sectional observational and machine-learning study |
| **Setting** | Nord Milano Mental Health Trust inpatient and outpatient services, Italy |
| **Population Type (adult, student, specific occupational groups)** | 32 adults with bipolar disorder |
| **Speaker Role** | Patient |

| | |
|---|---|
| **Elicitation Context** | Controlled |
| **Recording Modality** | Smartphone |
| **Sample size** | 32 |
| **Mental health outcome** | Depression, Bipolar/Mania |
| **Outcome Biomarker Domain** | Speech rate, Pause duration/frequency, Pitch, Response latency, Verbal output, Jitter, Shimmer, Harmonics-to-noise ratio, Word or pronoun use, Other |
| **Specific variables of marker studied** | Latency time, silence-to-phonation ratio, mean intraword time, word count, phonation duration, F0, jitter, shimmer, HNR, semantic distance |
| **clinical interpretability** | Yes |
| **Perceptial Label (if yes or Partial)** | Longer delays, more silence, fewer words, slower speech and less stable voice |
| **Outcome Measure/Tool?** | SCID-5 diagnosis, MADRS, YMRS |
| **Direction of Association** | Greater depressive symptoms were associated with longer intraword time, more silence, fewer words and greater vocal instability; manic symptoms were associated with shorter intraword time and lower jitter |
| **Effect Size/Model Performance/Statistical Significance** | MADRS correlations: intraword time $\rho=0.53$, silence-to-phonation ratio $\rho=0.42$, phonation percentage $\rho=-0.42$; depression model $R^2=0.25$ for NLP features and 0.11 for acoustic features |
| **Main Author Conclusions** | Speech and language features collected by smartphone were associated with bipolar mood severity, particularly depressive symptoms |
| **Key Limitations** | Very small sample, cross-sectional design, few participants with severe mania, possible overfitting, medication and comorbidity confounding, no external validation |
| **Notes** | NLP and conversational markers outperformed acoustic markers for depressive severity; manic prediction models were not reliable and results differed by sex. |

## Study 11 | Cummins et al. (2023)

| | |
|---|---|
| **Study design** | Longitudinal observational cohort study |
| **Setting** | RADAR-MDD study in the United Kingdom, Netherlands and Spain |
| **Population Type (adult, student, specific occupational groups)** | Adults with recurrent major depressive disorder speaking English, Dutch or Spanish |
| **Speaker Role** | Patient |
| **Elicitation Context** | Controlled |
| **Recording Modality** | Smartphone |
| **Sample size** | 461 |
| **Mental health outcome** | Depression |
| **Outcome Biomarker Domain** | Speech rate, Pause duration/frequency, Pitch, Vocal intensity/loudness, Voice quality, Jitter, Shimmer, Harmonics-to-noise ratio, Formant frequencies, Verbal output |
| **Specific variables of marker studied** | Speaking rate, articulation rate, intensity, pause duration, phonation ratio, pitch, jitter, shimmer, HNR and formants |
| **clinical interpretability** | Yes |

| | |
|---|---|
| **Perceptial Label (if yes or Partial)** | Slower, quieter speech with some longer pauses and reduced vocal output |
| **Outcome Measure/Tool?** | DSM-5 MDD diagnosis, IDS-SR, PHQ-8 |
| **Direction of Association** | Greater depressive severity was consistently associated with slower speaking and articulation rates and lower intensity across all three languages; pause and other acoustic findings were less consistent |
| **Effect Size/Model Performance/Statistical Significance** | Scripted speaking-rate β: UK –0.20, Netherlands –0.44, Spain –0.27; intensity β: UK –0.28, Netherlands –0.43, Spain –0.34, with 95% CIs excluding zero |
| **Main Author Conclusions** | Slower and quieter speech were robust multilingual markers of depressive severity in remotely collected longitudinal recordings |
| **Key Limitations** | Cohort-level rather than within-person findings, uncontrolled recording conditions, whole-recording averages, moderate symptom severity, possible reading-ability effects, 21% lacked longitudinal speech data |
| **Notes** | Speech was collected every two weeks for up to 18 months using scripted reading and prompted free-response tasks; the strongest cross-language findings came from the scripted task. |

## Study 12 | Cummins et al. (2015)

| | |
|---|---|
| **Study design** | Secondary analysis of two depression speech corpora |
| **Setting** | AVEC-2013 German recordings and Mundt-35 US clinical telephone recordings |
| **Population Type (adult, student, specific occupational groups)** | Participants with varying depressive symptom severity across two datasets |
| **Speaker Role** | Patient |
| **Elicitation Context** | Controlled |
| **Recording Modality** | Microphone, Telephone |
| **Sample size** | 70 speakers, 186 analyzed recordings |
| **Mental health outcome** | Depression |
| **Outcome Biomarker Domain** | MFCCs, Other |
| **Specific variables of marker studied** | Average weighted variance, acoustic movement, acoustic volume, probabilistic acoustic volume |
| **clinical interpretability** | Partial |
| **Perceptial Label (if yes or Partial)** | Less acoustically varied, smoother and more constrained speech |
| **Outcome Measure/Tool?** | Beck Depression Inventory, Quick Inventory of Depressive Symptomatology |
| **Direction of Association** | Higher depression severity was associated with reduced local acoustic variability, smoother acoustic trajectories and a smaller, more concentrated MFCC feature space |
| **Effect Size/Model Performance/Statistical Significance** | AWV correlations rs=–0.44 to –0.18, acoustic movement rs=–0.26 to –0.19, acoustic-volume measures rs=–0.32 to –0.20, feature-space concentration rs=–0.48 to –0.23, significant at $p<.05$ |
| **Main Author Conclusions** | Depression was consistently associated with reduced variability and movement within MFCC-based acoustic space across two datasets |

| | |
|---|---|
| **Key Limitations** | Secondary datasets, differing languages and recording channels, age and medication confounding, results varied by model configuration, limited direct clinical interpretability |
| **Notes** | The principal markers were derived from MFCC and GMM modeling rather than directly audible measurements; Partial reflects their perceptual correlate of reduced vocal variation. |

## Study 13 | Di et al. (2025)

| | |
|---|---|
| **Study design** | Multisite case-control, replication and genetic association study |
| **Setting** | CONVERGE psychiatric and general hospital sites across China |
| **Population Type (adult, student, specific occupational groups)** | Han Chinese women aged 30–60 with recurrent MDD and matched healthy controls |
| **Speaker Role** | Patient, Healthy Control |
| **Elicitation Context** | Controlled |
| **Recording Modality** | Other |
| **Sample size** | 8843 |
| **Mental health outcome** | Depression |
| **Outcome Biomarker Domain** | Pitch, Prosody/rhythm |
| **Specific variables of marker studied** | F0 and ΔF0 features, including pitch-change speed, pitch range, upper-range duration and pitch-change variability |
| **clinical interpretability** | Yes |
| **Perceptial Label (if yes or Partial)** | Lower-pitched speech with slower, less even pitch changes and less time in the upper vocal range |
| **Outcome Measure/Tool?** | DSM-IV MDD diagnosis, depressive symptom checklist |
| **Direction of Association** | MDD and current depressive symptoms were associated with slower pitch changes, lower pitch and altered pitch variability |
| **Effect Size/Model Performance/Statistical Significance** | 16 features replicated; combined $\|\beta\|=0.24–1.07$. Best XGBoost model: AUC 0.90, sensitivity 0.85, specificity 0.81 |
| **Main Author Conclusions** | Pitch features robustly distinguished recurrent MDD and reflected both current depressive symptoms and genetic susceptibility |
| **Key Limitations** | Women with recurrent MDD only, Chinese sample, pitch features only, recordings were not standardized, linguistic and emotional effects could not be fully separated |
| **Notes** | This uses the CONVERGE cohort also analyzed by Afshan et al.; overlap should be considered when counting independent study populations. |

## Study 14 | Di et al. (2021)

| | |
|---|---|
| **Study design** | Large multisite case-control machine-learning study |
| **Setting** | More than 20 hospitals and community sites across China |
| **Population Type (adult, student, specific occupational groups)** | Han Chinese women aged 30–60 with recurrent MDD and healthy controls |

| | |
|---|---|
| **Speaker Role** | Patient, Healthy Control |
| **Elicitation Context** | Controlled |
| **Recording Modality** | Other |
| **Sample size** | 1808 |
| **Mental health outcome** | Depression |
| **Outcome Biomarker Domain** | MFCCs, Other |
| **Specific variables of marker studied** | MFCC features, 200-dimensional MFCC i-vectors |
| **clinical interpretability** | No |
| **Perceptial Label (if yes or Partial)** | |
| **Outcome Measure/Tool?** | DSM-IV recurrent MDD diagnosis based on computerized clinical interviews |
| **Direction of Association** | Individual marker directions were not reported; i-vectors captured MFCC variability and improved MDD classification compared with MFCC features alone |
| **Effect Size/Model Performance/Statistical Significance** | All-utterance i-vector model: AUC 0.80, sensitivity 0.76, specificity 0.84; MFCC AUC 0.66. Performance stabilized at durations over 40 seconds |
| **Main Author Conclusions** | MFCC i-vectors improved depression classification and were robust when recordings exceeded 40 seconds |
| **Key Limitations** | Women only, utterance-level rather than person-level modeling, interview duration differed between groups, no independent external validation |
| **Notes** | The biomarkers were technical MFCC and i-vector representations, so Clinical Interpretability was coded No. This cohort overlaps with the CONVERGE studies by Afshan et al. and Di et al. |

## Study 15 | Dumpala et al. (2026)

| | |
|---|---|
| **Study design** | Cross-sectional observational and machine-learning validation study |
| **Setting** | Vocal Mind Project clinical research centre, Nova Scotia, Canada |
| **Population Type (adult, student, specific occupational groups)** | 239 English-speaking adults across no, mild, moderate and severe depression levels |
| **Speaker Role** | Patient, Healthy Control |
| **Elicitation Context** | Controlled |
| **Recording Modality** | Microphone |
| **Sample size** | 239 |
| **Mental health outcome** | Depression |
| **Outcome Biomarker Domain** | Pause duration/frequency, Vocal intensity/loudness, Prosody/rhythm, Voice quality, Formant frequencies, Other |
| **Specific variables of marker studied** | Pause duration, voiced rate, intensity, log energy, energy velocity, F3 variance, harmonic difference, spectral entropy |

| | |
|---|---|
| **clinical interpretability** | Yes |
| **Perceptial Label (if yes or Partial)** | Longer pauses, less voiced speech, and altered loudness and vocal energy |
| **Outcome Measure/Tool?** | MADRS, SCID-5 |
| **Direction of Association** | More severe depression was associated with longer pauses, lower voiced rate, altered intensity and energy, and greater F3 variance across emotional prompts |
| **Effect Size/Model Performance/Statistical Significance** | Significant feature correlations ranged from ρ=0.095–0.333; best 4-class balanced accuracy was 41.5% using selected features |
| **Main Author Conclusions** | Prosodic markers were the most informative and showed broadly consistent associations with depression severity across neutral, positive and negative speech |
| **Key Limitations** | English only, uneven severity and sex distribution, small held-out test set, weak individual feature effects, limited classification accuracy |
| **Notes** | The abstract reports 147 participants with MDD, while the Methods states 115 had SCID-5-confirmed MDD; this discrepancy should be checked. |

## Study 16 | Faurholt-Jepsen et al. (2016)

| | |
|---|---|
| **Study design** | Longitudinal observational machine-learning study |
| **Setting** | Copenhagen Clinic for Affective Disorders, Denmark |
| **Population Type (adult, student, specific occupational groups)** | 28 adults with bipolar disorder monitored for 12 weeks |
| **Speaker Role** | Patient |
| **Elicitation Context** | Passive/Ambient |
| **Recording Modality** | Smartphone, Telephone |
| **Sample size** | 28 |
| **Mental health outcome** | Depression, Bipolar/Mania |
| **Outcome Biomarker Domain** | Pitch, Other |
| **Specific variables of marker studied** | 6,552 openSMILE emolarge features, including pitch and statistical functionals |
| **clinical interpretability** | No |
| **Perceptial Label (if yes or Partial)** | |
| **Outcome Measure/Tool?** | SCAN, HAMD-17, YMRS |
| **Direction of Association** | Individual voice-feature directions were not reported; voice models classified manic or mixed states more accurately than depressive states |
| **Effect Size/Model Performance/Statistical Significance** | Voice-only user-independent models: depression AUC 0.78, accuracy 0.68; mania or mixed AUC 0.89, accuracy 0.74 |
| **Main Author Conclusions** | Voice features from everyday phone calls showed potential as objective markers of bipolar affective states |
| **Key Limitations** | Small sample, only 13 patients had sufficient voice data for any individual model, 12-week follow-up, Android-only system, many undocumented features, possible missing calls and medication effects |

| | |
|---|---|
| **Notes** | Clinical interpretability was rated No because the model used thousands of largely undocumented derived features and did not report the influential individual markers. |

## Study 17 | Figueroa et al. (2024)

| | |
|---|---|
| **Study design** | Cross-sectional analytical study |
| **Setting** | High-vulnerability secondary schools in Temuco, Chile |
| **Population Type (adult, student, specific occupational groups)** | Adolescents aged 14–19 with and without suicidal risk |
| **Speaker Role** | Patient, Healthy Control |
| **Elicitation Context** | Controlled |
| **Recording Modality** | Microphone |
| **Sample size** | 98 |
| **Mental health outcome** | Other |
| **Outcome Biomarker Domain** | Pitch, Prosody/rhythm, Voice quality, Jitter, Shimmer, Harmonics-to-noise ratio, Formant frequencies |
| **Specific variables of marker studied** | F0, F1–F3, jitter, shimmer, HNR, formant energy, vowel duration, F0 variation |
| **clinical interpretability** | Partial |
| **Perceptial Label (if yes or Partial)** | Higher and more variable pitch with altered vocal stability and resonance |
| **Outcome Measure/Tool?** | Okasha Suicidality Scale, Beck Depression Inventory-II |
| **Direction of Association** | Adolescents with suicidal risk generally had higher pitch, formant frequencies, HNR, and greater frequency variability, with sex- and vowel-specific differences |
| **Effect Size/Model Performance/Statistical Significance** | F0 differed across all vowels, $p=0.001$–$0.009$, $d=0.59$–$0.73$; significant HNR, formant and jitter differences had $p<0.05$ |
| **Main Author Conclusions** | Suicidal risk was associated with measurable pitch, voice-quality, and resonance differences during text reading |
| **Key Limitations** | Convenience sample, very small male at-risk group, multiple comparisons, cross-sectional design, no external validation, limited gender diversity |
| **Notes** | Outcome was coded Other because the primary outcome was suicidal risk rather than depression. The paper reports 98 participants, but sex and risk totals sum to 97. |

## Study 18 | Gao et al. (2022)

| | |
|---|---|
| **Study design** | Experimental sleep-deprivation and machine-learning study |
| **Setting** | Tianjin Institute of Environmental and Operational Medicine, China |
| **Population Type (adult, student, specific occupational groups)** | 15 healthy male students aged 23–25 undergoing 36 hours of sleep deprivation |
| **Speaker Role** | Healthy Control |
| **Elicitation Context** | Controlled |

| | |
|---|---|
| **Recording Modality** | Microphone |
| **Sample size** | 15 |
| **Mental health outcome** | Fatigue |
| **Outcome Biomarker Domain** | Pitch, Vocal intensity/loudness, Voice quality, Jitter, Shimmer, Harmonics-to-noise ratio, MFCCs, Other |
| **Specific variables of marker studied** | F0, energy, loudness, zero-crossing rate, HNR, jitter, shimmer, MFCC1–12 |
| **clinical interpretability** | Partial |
| **Perceptial Label (if yes or Partial)** | Changes in pitch, loudness, energy and vocal stability with fatigue |
| **Outcome Measure/Tool?** | Stanford Sleepiness Scale, P300, salivary cortisol, sleep-deprivation duration |
| **Direction of Association** | No individual vowel feature changed significantly; combined acoustic patterns distinguished fatigued from non-fatigued speech |
| **Effect Size/Model Performance/Statistical Significance** | Multi-vowel SVM accuracy 94%; single-vowel accuracy 75%–88%; long-speech CART accuracy 76%, recall 81%, F1 76% |
| **Main Author Conclusions** | Combined acoustic features showed potential for rapid, non-invasive detection of experimentally induced fatigue |
| **Key Limitations** | Very small male-only sample, narrow age range, induced acute fatigue, no external validation, possible overfitting, dialects and other populations not assessed |
| **Notes** | The abstract reports 81% accuracy for long speech, while the detailed results report 76% accuracy and 81% recall for the best CART model. |

## Study 19 | Gaur et al. (2024)

| | |
|---|---|
| **Study design** | Prospective observational sleep-deprivation and machine-learning study |
| **Setting** | Defence Institute of Physiology and Allied Sciences, India |
| **Population Type (adult, student, specific occupational groups)** | 32 healthy Indian men aged 20–40 undergoing one night of sleep deprivation |
| **Speaker Role** | Healthy Control |
| **Elicitation Context** | Controlled |
| **Recording Modality** | Microphone |
| **Sample size** | 32 |
| **Mental health outcome** | Fatigue |
| **Outcome Biomarker Domain** | Harmonics-to-noise ratio |
| **Specific variables of marker studied** | HNR from sustained vowel /a/ |
| **clinical interpretability** | Partial |
| **Perceptial Label (if yes or Partial)** | Noisier, breathier or less harmonic voice |

| | |
|---|---|
| **Outcome Measure/Tool?** | Visual reaction time, one-night sleep-deprivation protocol |
| **Direction of Association** | HNR decreased significantly at 3 AM compared with baseline, then increased by 7 AM, suggesting circadian variation in fatigue-related voice quality |
| **Effect Size/Model Performance/Statistical Significance** | Baseline HNR 26.90 dB, 3 AM 24.16 dB, 7 AM 28.11 dB, ANOVA p=0.0015; KNN test accuracy 57%, AUC 0.71 at 3 AM and 0.75 at 7 AM |
| **Main Author Conclusions** | HNR showed potential as a simple acoustic marker of fatigue after overnight sleep deprivation |
| **Key Limitations** | Small male-only sample, one-night protocol, sustained vowel only, modest classification accuracy, no external validation |
| **Notes** | HNR was significant only at 3 AM versus baseline, suggesting that circadian timing may partly explain the observed changes. |

## Study 20 | Greeley et al. (2007)

| | |
|---|---|
| **Study design** | Repeated-measures experimental study using three fatigue protocols |
| **Setting** | FAA, US Air Force field setting, and Creare workplace studies |
| **Population Type (adult, student, specific occupational groups)** | 31 healthy adults undergoing sleep deprivation, restricted sleep, or a normal workday |
| **Speaker Role** | Healthy Control |
| **Elicitation Context** | Controlled |
| **Recording Modality** | Microphone, Other |
| **Sample size** | 31 |
| **Mental health outcome** | Fatigue |
| **Outcome Biomarker Domain** | MFCCs, Other |
| **Specific variables of marker studied** | 36-component MFCC voice vector, Voice Correlation metric for individual speech sounds |
| **clinical interpretability** | No |
| **Perceptial Label (if yes or Partial)** | |
| **Outcome Measure/Tool?** | Sleep onset latency, SAFTE model, Stanford Sleepiness Scale, fatigue and mood questionnaires |
| **Direction of Association** | Voice similarity to baseline decreased as time awake increased, especially for consonants requiring greater airflow |
| **Effect Size/Model Performance/Statistical Significance** | Voice Correlation versus time awake: /p/ r=-0.89, /t/ r=-0.67; association between airflow and fatigue-tracking ability r=0.77, p=0.01 |
| **Main Author Conclusions** | MFCC-based changes in selected speech sounds tracked fatigue and predicted performance decline across different operational settings |
| **Key Limitations** | Small heterogeneous samples, group-average analyses, different protocols and recording devices, individual-specific features, no sensitivity or specificity estimates |
| **Notes** | High-airflow consonants, particularly /p/, were more sensitive to fatigue than lower-airflow sounds. |

## Study 21 | Gumus et al. (2023)

| | |
|---|---|
| **Study design** | Longitudinal remote repeated-measures study |
| **Setting** | Remote app-based study in Canada and the United States |
| **Population Type (adult, student, specific occupational groups)** | 16 English-speaking community adults aged 21–54, with or without psychiatric diagnoses |
| **Speaker Role** | Patient, Healthy Control |
| **Elicitation Context** | Naturalistic |
| **Recording Modality** | Smartphone |
| **Sample size** | 16 |
| **Mental health outcome** | Depression |
| **Outcome Biomarker Domain** | Vocal intensity/loudness, Jitter, Sentiment/emotional tone |
| **Specific variables of marker studied** | Intensity range, local jitter, sentiment valence, sentiment dominance |
| **clinical interpretability** | Yes |
| **Perceptial Label (if yes or Partial)** | Less variation in loudness, rougher or less stable voice, and less positive or confident language |
| **Outcome Measure/Tool?** | Modified daily PHQ-9, standard bi-weekly PHQ-9 |
| **Direction of Association** | Higher daily depression symptoms were associated with lower intensity range, increased jitter, and use of less positive and less dominant words |
| **Effect Size/Model Performance/Statistical Significance** | Intensity range rrm=-0.22, jitter rrm=0.24, valence rrm=-0.34, dominance rrm=-0.31, all p<0.001 |
| **Main Author Conclusions** | Daily speech assessments captured within-person depression fluctuations that were not detected using only three bi-weekly assessments |
| **Key Limitations** | Very small non-clinical sample, COVID-19 context, modified and standard PHQ-9 scales differed, only three bi-weekly observations, no independent validation |
| **Notes** | The daily task was an open-ended prompted journal recorded at home, so it was coded Naturalistic. The study was funded by Winterlight Labs and most authors were employees. |

## Study 22 | Hartnagel et al. (2024)

| | |
|---|---|
| **Study design** | Longitudinal intervention and machine-learning study |
| **Setting** | Central Institute of Mental Health inpatient unit, Mannheim, Germany |
| **Population Type (adult, student, specific occupational groups)** | 22 adults hospitalized with moderate or severe acute depression undergoing sleep-deprivation therapy |
| **Speaker Role** | Patient |
| **Elicitation Context** | Naturalistic |
| **Recording Modality** | Smartphone, Video |
| **Sample size** | 22 |

| | |
|---|---|
| **Mental health outcome** | Depression |
| **Outcome Biomarker Domain** | Speech rate, Pause duration/frequency, Pitch, Vocal intensity/loudness, Voice quality, Jitter, Shimmer, Harmonics-to-noise ratio, MFCCs, Other |
| **Specific variables of marker studied** | 89 eGeMAPS acoustic features plus speech rate |
| **clinical interpretability** | Partial |
| **Perceptial Label (if yes or Partial)** | Changes in speaking speed, pausing, pitch variation and overall voice quality |
| **Outcome Measure/Tool?** | Modified momentary ADS-K, baseline MADRS and ICD-10 diagnosis |
| **Direction of Association** | Individual feature directions were not reported; speech predicted depression severity only when the model had prior data from the same patients |
| **Effect Size/Model Performance/Statistical Significance** | Best odd-even XGBoost model: $R^2=0.339$, MAE=0.38, $p<0.001$; models tested on unseen patients performed no better than chance |
| **Main Author Conclusions** | Speech features showed moderate within-patient prediction of momentary depression severity but did not generalize to new patients |
| **Key Limitations** | Small sample, only 22 analyzable patients, short inpatient study, treatment-specific symptom changes, no external validation, performance depended on patient overlap |
| **Notes** | Initial sample was 30, but 8 patients were excluded. Findings support personalized rather than generalized models and use the same dataset as Wadle et al. |

## Study 23 | Hashim et al. (2017)

| | |
|---|---|
| **Study design** | Prospective observational prediction study |
| **Setting** | Vanderbilt University emergency department and primary care clinics, Tennessee, USA |
| **Population Type (adult, student, specific occupational groups)** | 116 adults with high-risk depression, nonsuicidal depression, or remitted depression |
| **Speaker Role** | Patient |
| **Elicitation Context** | Controlled |
| **Recording Modality** | Microphone |
| **Sample size** | 116 |
| **Mental health outcome** | Depression |
| **Outcome Biomarker Domain** | Pause duration/frequency, Vocal intensity/loudness, Prosody/rhythm, MFCCs, Other |
| **Specific variables of marker studied** | Power spectral density bands, MFCC1–13, voiced-unvoiced-silence transitions, voiced and silent interval distributions |
| **clinical interpretability** | Partial |
| **Perceptial Label (if yes or Partial)** | Changes in loudness, speech cadence, timing, and pausing |
| **Outcome Measure/Tool?** | Hamilton Depression Rating Scale, Beck Depression Inventory-II |
| **Direction of Association** | Individual feature directions were not reported; timing features predicted female HAMD and male BDI-II scores, while combined timing and spectral features predicted male HAMD scores |

| | |
|---|---|
| **Effect Size/Model Performance/Statistical Significance** | HAMD MAE: 1.43 points in men and 1.69 in women; 90.5% and 87.9% were predicted within 3 points. BDI-II MAE: 4.86 in men and 8.45 in women |
| **Main Author Conclusions** | Voice timing and spectral features predicted clinician-rated depression severity more accurately than self-reported BDI-II scores |
| **Key Limitations** | Single-center convenience sample, small sex-specific HAMD samples, many candidate predictors, feature-selection and overfitting risk, smoking and professional voice use not assessed |
| **Notes** | Timing and spectral predictors differed by sex. HAMD models used 23 participants and BDI-II models used 93, although the total recruited sample was 116. |

## Study 24 | Hiß et al. (2025)

| | |
|---|---|
| **Study design** | Transdiagnostic case-control machine-learning study |
| **Setting** | RWTH Aachen University psychiatric inpatient and outpatient services, Germany |
| **Population Type (adult, student, specific occupational groups)** | 150 German-speaking adults, including 62 with MDD, 47 with schizophrenia-spectrum disorder, and 41 healthy controls |
| **Speaker Role** | Patient, Healthy Control |
| **Elicitation Context** | Controlled |
| **Recording Modality** | Microphone |
| **Sample size** | 150 |
| **Mental health outcome** | Depression, Other |
| **Outcome Biomarker Domain** | Pause duration/frequency, Pitch, Vocal intensity/loudness, Voice quality, Jitter, Shimmer, Harmonics-to-noise ratio, Formant frequencies, MFCCs, Prosody/rhythm, Other |
| **Specific variables of marker studied** | Loudness, pause duration, pitch variability, voiced-segment rate, jitter, shimmer, formants, spectral slope, spectral flux, MFCCs |
| **clinical interpretability** | Partial |
| **Perceptial Label (if yes or Partial)** | Quieter, more monotone and fragmented speech with longer pauses and less stable voice |
| **Outcome Measure/Tool?** | SCID-5, ICD-10 diagnosis, GRID-HAMD-21 |
| **Direction of Association** | Both patient groups generally showed lower loudness, longer and more variable pauses, greater vocal instability, and reduced dynamic modulation; SSD showed more monotony and articulatory rigidity, while MDD showed greater pitch and resonance variability |
| **Effect Size/Model Performance/Statistical Significance** | Best three-class RF accuracy 56.7%, macro F1 57.2% for a happy-video task; HAMD interview accuracy 56.1%; reading accuracy 45.4%; MDD-specific classification reached 75.2% |
| **Main Author Conclusions** | Standardized acoustic features moderately differentiated MDD, SSD, and healthy controls, with emotionally engaging speech tasks outperforming reading |
| **Key Limitations** | Single-site sample, demographic and medication confounding, possible overfitting, no independent external validation, repeated recordings from some participants |
| **Notes** | The primary mental-health categories were MDD and schizophrenia-spectrum disorder, so schizophrenia was coded Other. Measurements were taken twice for some participants, producing 310 recordings, but the participant sample was 150. |

## Study 25 | Immel et al. (2026)

| | |
|---|---|
| **Study design** | Cross-sectional observational and machine-learning study |
| **Setting** | Single-session tablet-based assessment in Germany |
| **Population Type (adult, student, specific occupational groups)** | 96 healthy German-speaking adults aged 18–65 with varying loneliness levels |
| **Speaker Role** | Healthy Control |
| **Elicitation Context** | Controlled |
| **Recording Modality** | Other |
| **Sample size** | 96 |
| **Mental health outcome** | Other |
| **Outcome Biomarker Domain** | Pause duration/frequency, Pitch, Vocal intensity/loudness, Verbal output, Voice quality, Harmonics-to-noise ratio, Other |
| **Specific variables of marker studied** | Speech ratio, number of pauses, phonation time, amplitude kurtosis, sound-to-noise ratio, HNR, mean pitch |
| **clinical interpretability** | Yes |
| **Perceptial Label (if yes or Partial)** | Less speech, shorter phonation, poorer voice quality, higher pitch, and more irregular loudness |
| **Outcome Measure/Tool?** | Revised UCLA Loneliness Scale, BDI-II, Liebowitz Social Anxiety Scale, MINI |
| **Direction of Association** | Higher loneliness was linked to lower speech ratio and more irregular loudness in women, and fewer pauses, shorter phonation, poorer voice quality, and higher pitch in men |
| **Effect Size/Model Performance/Statistical Significance** | Picture-description model: women $R^2$=0.06, MAE=11.49, men $R^2$=0.16, MAE=10.25; storytelling models were not significant |
| **Main Author Conclusions** | Speech from a standardized picture-description task modestly predicted loneliness, with different patterns in women and men |
| **Key Limitations** | Healthy non-clinical sample, small sex-stratified groups, cross-sectional design, no external validation, trait loneliness chronicity unclear, individual effects were small |
| **Notes** | Loneliness was coded Other. Picture description and emotional storytelling were analyzed, but only picture-description speech significantly predicted loneliness. |

## Study 26 | Jiang et al. (2018)

| | |
|---|---|
| **Study design** | Case-control machine-learning classification study |
| **Setting** | Psychiatric clinical assessment and laboratory speech recording in China |
| **Population Type (adult, student, specific occupational groups)** | 170 native Mandarin-speaking adults, including 85 with depression and 85 matched healthy controls |
| **Speaker Role** | Patient, Healthy Control |
| **Elicitation Context** | Controlled |
| **Recording Modality** | Microphone |
| **Sample size** | 170 |
| **Mental health outcome** | Depression |

| | |
|---|---|
| **Outcome Biomarker Domain** | Pitch, Vocal intensity/loudness, Prosody/rhythm, Jitter, Shimmer, MFCCs, Other |
| **Specific variables of marker studied** | F0, loudness, pitch onset and duration, jitter, shimmer, MFCCs, spectral flux, centroid, entropy, roll-off, band energy, glottal features |
| **clinical interpretability** | Partial |
| **Perceptial Label (if yes or Partial)** | Changes in pitch, loudness, vocal stability, spectral qualities, and voice production |
| **Outcome Measure/Tool?** | DSM-IV psychiatric diagnosis, PHQ-9 |
| **Direction of Association** | Individual feature directions were not reported; prosodic and spectral features generally classified depression better than glottal features, and performance differed by sex |
| **Effect Size/Model Performance/Statistical Significance** | Best subject-level ensemble model: male accuracy 81.8%, sensitivity 78.1%, specificity 85.3%; female accuracy 75.0%, sensitivity 79.3%, specificity 70.6% |
| **Main Author Conclusions** | Combining multiple complementary speech-feature groups improved depression classification compared with individual and conventional ensemble classifiers |
| **Key Limitations** | Single-language sample, no external validation, very high-dimensional feature set, limited interpretability, possible overfitting despite subject-independent cross-validation |
| **Notes** | Participants completed interview, reading, and picture-description tasks. Models were sex-specific, and each participant contributed 29 recordings. |

## Study 27 | Kaczmarek-Majer et al. (2025)

| | |
|---|---|
| **Study design** | Prospective longitudinal observational and prediction study |
| **Setting** | Bipolar disorder treatment centres in Warsaw and Pozna■, Poland |
| **Population Type (adult, student, specific occupational groups)** | 51 adults with bipolar I or II disorder monitored for a mean of 208 days |
| **Speaker Role** | Patient |
| **Elicitation Context** | Passive/Ambient |
| **Recording Modality** | Smartphone, Telephone |
| **Sample size** | 51 |
| **Mental health outcome** | Depression, Bipolar/Mania |
| **Outcome Biomarker Domain** | Speech rate, Pitch, Vocal intensity/loudness, Verbal output, Voice quality, Jitter, Shimmer, Harmonics-to-noise ratio, Formant frequencies, MFCCs, Other |
| **Specific variables of marker studied** | Loudness, energy, F0, speaking rate and duration, call duration, spectral flux, spectral harmonicity, sharpness, jitter, shimmer, HNR, formants, MFCCs |
| **clinical interpretability** | Yes |
| **Perceptial Label (if yes or Partial)** | Changes in loudness, pitch, speaking amount, clarity, sharpness, and vocal roughness |
| **Outcome Measure/Tool?** | HDRS-17, YMRS, ICD-10 bipolar diagnosis |
| **Direction of Association** | In men, greater mania was associated with louder, higher-pitched, longer, clearer, sharper, and rougher speech, while greater depression was associated with quieter and less clear speech; findings in women were weaker or often opposite |

| | |
|---|---|
| **Effect Size/Model Performance/Statistical Significance** | Best phase-prediction accuracy 70.9%–71.4%; lower-cutoff sensitivity was 61%–62% for depression and 52%–56% for mania or mixed states |
| **Main Author Conclusions** | Daily phone-call acoustics were associated with bipolar mood severity and showed moderate ability to predict affective phase |
| **Key Limitations** | Small sample, medication effects not assessed, sex-specific findings reduced generalizability, call-level cross-validation may overestimate performance, background noise and different phones were uncontrolled |
| **Notes** | The app processed only the patient's first 5 minutes of daily phone calls and deleted recordings after extracting features. Strong sex differences indicate that bipolar speech markers may require sex-specific interpretation. |

## Study 28 | Kim et al. (2023)

| | |
|---|---|
| **Study design** | Case-control deep-learning classification study |
| **Setting** | Two university hospital sites in South Korea |
| **Population Type (adult, student, specific occupational groups)** | 318 Korean-speaking adults, including 153 patients with MDD and 165 healthy controls |
| **Speaker Role** | Patient, Healthy Control |
| **Elicitation Context** | Controlled |
| **Recording Modality** | Smartphone |
| **Sample size** | 318 |
| **Mental health outcome** | Depression |
| **Outcome Biomarker Domain** | MFCCs, Pitch, Vocal intensity/loudness, Prosody/rhythm, Voice quality, Other |
| **Specific variables of marker studied** | Log-Mel spectrograms, MFCC1–12, eGeMAPS and COMPARE acoustic features |
| **clinical interpretability** | Partial |
| **Perceptial Label (if yes or Partial)** | More monotonous speech with reduced acoustic variability |
| **Outcome Measure/Tool?** | DSM criteria, HAM-D, PHQ-9 |
| **Direction of Association** | Participants with MDD showed lower MFCC3 variability across vowel, digit and passage tasks; the deep CNN learned additional depression-related spectrogram patterns that were not individually interpretable |
| **Effect Size/Model Performance/Statistical Significance** | Best passage CNN: accuracy 78.14%, precision 76.83%, recall 77.90%, F1 77.27%, AUC 0.86 |
| **Main Author Conclusions** | Text-dependent Korean passage reading enabled moderately accurate smartphone-based depression classification and outperformed conventional acoustic-feature models |
| **Key Limitations** | Korean-only sample, controlled quiet-room recordings, one recording per participant, limited external validation, deep features were difficult to interpret, education and substance-use characteristics differed between groups |
| **Notes** | The neutral passage task performed better than vowel and digit tasks. Participant-independent splits and 10-fold cross-validation were used. |

## Study 29 | König et al. (2022)

| | |
|---|---|
| **Study design** | Cross-sectional observational and machine-learning study |
| **Setting** | University laboratory settings in Germany |
| **Population Type (adult, student, specific occupational groups)** | 118 healthy university students aged 20–30, including 25 above the cut-off for clinically relevant depressive symptoms |
| **Speaker Role** | Healthy Control |
| **Elicitation Context** | Controlled |
| **Recording Modality** | Other |
| **Sample size** | 118 |
| **Mental health outcome** | Depression |
| **Outcome Biomarker Domain** | Speech rate, Pause duration/frequency, Pitch, Vocal intensity/loudness, Verbal output, Jitter, Shimmer, MFCCs, Word or pronoun use, Other |
| **Specific variables of marker studied** | Word count, speech ratio, pause duration, F0, intensity variability, jitter, shimmer, harmonic difference, MFCCs, linguistic and semantic features |
| **clinical interpretability** | Yes |
| **Perceptial Label (if yes or Partial)** | More talking, altered pausing, pitch, loudness variability, and vocal stability |
| **Outcome Measure/Tool?** | Allgemeine Depressionsskala self-report questionnaire |
| **Direction of Association** | Participants above the depressive-symptom cut-off used more words in both positive and negative stories; other individual features did not differ after controlling for word count, but several acoustic and linguistic features contributed to prediction |
| **Effect Size/Model Performance/Statistical Significance** | Cut-off classification accuracy 93.2%, Nagelkerke $R^2$=0.784; depression-score prediction MAE=3.90 |
| **Main Author Conclusions** | Subtle depressive symptoms in a non-clinical sample were associated mainly with greater verbal output, while combined speech features predicted symptom status and severity |
| **Key Limitations** | Non-clinical student sample, mostly women, short speech tasks, no diagnostic interview, small above-cut-off group, high-dimensional modeling and no external validation |
| **Notes** | Participants described positive and negative life events. Depression was defined by questionnaire cut-off rather than clinical diagnosis. |

## Study 30 | König et al. (2021)

| | |
|---|---|
| **Study design** | Observational pilot and machine-learning regression study |
| **Setting** | Health care settings in Nice and the Provence-Alpes-Côte d'Azur region, France |
| **Population Type (adult, student, specific occupational groups)** | 89 French-speaking health professionals working during the COVID-19 pandemic |
| **Speaker Role** | Healthy Control |
| **Elicitation Context** | Controlled |

| | |
|---|---|
| **Recording Modality** | Telephone |
| **Sample size** | 89 |
| **Mental health outcome** | Stress |
| **Outcome Biomarker Domain** | Speech rate, Pause duration/frequency, Pitch, Vocal intensity/loudness, Prosody/rhythm, Voice quality, Jitter, Shimmer, Harmonics-to-noise ratio, Formant frequencies, MFCCs, Verbal output, Other |
| **Specific variables of marker studied** | F0 and pitch range, speech rate, articulation rate, speaking duration, speech ratio, MFCCs, energy, loudness, spectral features, jitter, shimmer, HNR |
| **clinical interpretability** | Partial |
| **Perceptial Label (if yes or Partial)** | Changes in pitch range, vocal energy, speech timing, and voice quality |
| **Outcome Measure/Tool?** | Perceived Stress Scale, Motivation Stress Affect questionnaire |
| **Direction of Association** | Stress was associated mainly with MFCC features in women and lower F0 or narrower pitch range in men; associations varied by task and sex |
| **Effect Size/Model Performance/Statistical Significance** | Best overall prediction used positive storytelling, MAE 5.31; male MAE 3.84 and female MAE 5.56 |
| **Main Author Conclusions** | Short telephone speech tasks showed modest ability to estimate perceived stress in health professionals during the pandemic |
| **Key Limitations** | Small voluntary sample, cross-sectional design, moderate correlations, self-reported stress, sex-stratified analyses, no external validation, possible selection and recording effects |
| **Notes** | Positive storytelling produced the best stress predictions. Most markers were task- and sex-dependent, and only 16% requested clinical follow-up. |

## Study 31 | Lee et al. (2021)

| | |
|---|---|
| **Study design** | Case-control machine-learning classification study |
| **Setting** | Korean Longitudinal Study on Cognitive Aging and Dementia, Seoul National University Bundang Hospital, South Korea |
| **Population Type (adult, student, specific occupational groups)** | 204 community-dwelling Korean adults aged 60 or older, including 61 with MDD and 143 healthy controls |
| **Speaker Role** | Patient, Healthy Control |
| **Elicitation Context** | Controlled |
| **Recording Modality** | Microphone |
| **Sample size** | 204 |
| **Mental health outcome** | Depression |
| **Outcome Biomarker Domain** | Pitch, Vocal intensity/loudness, Prosody/rhythm, Voice quality, Jitter, Shimmer, Harmonics-to-noise ratio, Formant frequencies, MFCCs, Other |
| **Specific variables of marker studied** | F0, loudness, energy, MFCCs, spectral features, formants, jitter, shimmer, HNR, correlation distances between emotional sentences |
| **clinical interpretability** | Partial |
| **Perceptial Label (if yes or Partial)** | Quieter speech and reduced vocal change between positive and negative emotional sentences |

| | |
|---|---|
| **Outcome Measure/Tool?** | MINI-K, DSM-IV-TR MDD diagnosis, Korean Geriatric Depression Scale |
| **Direction of Association** | Lower loudness and spectral-energy features distinguished depressed men, while pitch-related features distinguished depressed women; MDD participants showed blunted vocal reactivity to positive and negative sentences |
| **Effect Size/Model Performance/Statistical Significance** | Male AUC 0.911, sensitivity 95.0%, specificity 88.1%, accuracy 85.6%; female AUC 0.799, sensitivity 73.4%, specificity 86.2%, accuracy 77.3 |
| **Main Author Conclusions** | Sex-specific acoustic patterns and reduced emotional voice reactivity showed good performance for screening late-life MDD |
| **Key Limitations** | Small MDD sample, especially men, psychotropic medication use, no independent external validation, controlled reading task, homogeneous elderly Korean sample, unclear underlying mechanism |
| **Notes** | The model used changes between mood-inducing sentences rather than raw voice features. Depressed participants showed less differentiation between positive and negative speech conditions. |

## Study 32 | Lin et al. (2026)

| | |
|---|---|
| **Study design** | Multicohort cross-sectional and longitudinal observational study |
| **Setting** | Three psychiatric hospitals in China, with home and clinical recordings |
| **Population Type (adult, student, specific occupational groups)** | 1857 adults across discovery, independent validation, and 8-week longitudinal MDD cohorts |
| **Speaker Role** | Patient, Healthy Control |
| **Elicitation Context** | Controlled |
| **Recording Modality** | Smartphone, Other |
| **Sample size** | 1857 |
| **Mental health outcome** | Depression |
| **Outcome Biomarker Domain** | Pitch, Vocal intensity/loudness, Prosody/rhythm, Voice quality, Jitter, Formant frequencies, MFCCs, Shimmer, Other |
| **Specific variables of marker studied** | ComParE acoustic features covering MFCCs, spectral band energy, spectral shape and modulation, energy, F0, formants, jitter, shimmer, and harmonicity |
| **clinical interpretability** | Partial |
| **Perceptial Label (if yes or Partial)** | Lower vocal energy, flatter and less dynamic speech, reduced articulatory precision, and altered pitch and voice stability |
| **Outcome Measure/Tool?** | MINI, DSM-IV MDD diagnosis, HAMD-24, PHQ-9, SDS, HAMA |
| **Direction of Association** | Across cohorts, depression was associated mainly with MFCC and spectral-energy changes, reduced energy and formant measures, and less vocal dynamism; distinct features reflected depressed mood, somatic symptoms, retardation, and diurnal variation |
| **Effect Size/Model Performance/Statistical Significance** | 69 features showed replicated moderate effects and were reduced to 23 nonredundant markers; 38 features changed significantly during treatment, many approaching healthy levels within 2 weeks |
| **Main Author Conclusions** | A compact subset of acoustic features replicated across cohorts, reflected specific symptom dimensions, and tracked improvement during antidepressant treatment |
| **Key Limitations** | Smaller validation cohort, symptom-factor data available in only one cohort, 8-week follow-up, medication and comorbidity confounding, variable devices and speech content, reliability concerns for some ComParE features |

| | |
|---|---|
| **Notes** | The longitudinal cohort included 93 patients at baseline, falling to 59 by week 8. Spectral-shape and modulation features changed more rapidly with treatment than energy and voice-quality features. |

## Study 33 | Lin et al. (2022)

| | |
|---|---|
| **Study design** | Case-control deep-learning classification study |
| **Setting** | Peking University Sixth Hospital, Tangshan Fifth People’s Hospital, and remote settings in China |
| **Population Type (adult, student, specific occupational groups)** | 103 Mandarin-speaking adults aged 50–65, including 56 with MDD and 47 healthy controls |
| **Speaker Role** | Patient, Healthy Control |
| **Elicitation Context** | Controlled |
| **Recording Modality** | Smartphone |
| **Sample size** | 103 |
| **Mental health outcome** | Depression |
| **Outcome Biomarker Domain** | Other |
| **Specific variables of marker studied** | Mel-scale filterbanks, raw-speech contextual embeddings, log-Mel spectrograms |
| **clinical interpretability** | No |
| **Perceptial Label (if yes or Partial)** | |
| **Outcome Measure/Tool?** | MINI, DSM-5 MDD diagnosis, HAMD, HAMA, SDS |
| **Direction of Association** | Individual acoustic directions were not reported; the pretrained model learned latent speech patterns distinguishing MDD from controls, while remission samples generally resembled controls |
| **Effect Size/Model Performance/Statistical Significance** | AUC 0.868, sensitivity 82.14%, specificity 80.85%, positive predictive value 83.64%, negative predictive value 79.17 |
| **Main Author Conclusions** | A pretrained speech model showed good performance for detecting MDD from smartphone-recorded Mandarin reading samples in middle-aged and older adults |
| **Key Limitations** | Different recording environments between groups, sound-quality confounding, proprietary training dataset, fixed reading task, black-box features, limited age range, only four remission cases |
| **Notes** | The model used a separate clinical test sample, but poorer and noisier recordings were more common in patients and could have influenced classification. |

## Study 34 | Little et al. (2021)

| | |
|---|---|
| **Study design** | Case-control naturalistic wearable-monitoring study |
| **Setting** | Community-dwelling older adults in North East England |
| **Population Type (adult, student, specific occupational groups)** | 58 English-speaking adults aged over 60, including 29 with late-life depression and 29 age-matched healthy controls |

| | |
|---|---|
| **Speaker Role** | Patient, Healthy Control |
| **Elicitation Context** | Passive/Ambient |
| **Recording Modality** | Other |
| **Sample size** | 58 |
| **Mental health outcome** | Depression |
| **Outcome Biomarker Domain** | Verbal output, Other |
| **Specific variables of marker studied** | Proportion of daily speech detected, proportion of speech produced by the wearer |
| **clinical interpretability** | Yes |
| **Perceptial Label (if yes or Partial)** | Much less daily speech exposure and speech production |
| **Outcome Measure/Tool?** | MINI, DSM-IV MDD diagnosis, MADRS, GDS-15 |
| **Direction of Association** | Participants with late-life depression encountered substantially less speech and produced a smaller proportion of speech than controls; both measures were associated with attention and psychomotor speed |
| **Effect Size/Model Performance/Statistical Significance** | Total speech: 2% of the day in LLD versus 13% in controls; wearer speech: 3% versus 11%; both measures separated groups with 100% accuracy |
| **Main Author Conclusions** | Wrist-worn passive speech monitoring provided an objective marker of reduced real-world social and verbal activity in late-life depression |
| **Key Limitations** | Small cross-sectional sample, classifier trained only in healthy adults, speech recordings could not be manually validated in the study sample, possible effects of living situation and other social factors, no longitudinal validation |
| **Notes** | The wearable detected speech quantity rather than acoustic voice characteristics. Speech activity did not correlate with depression severity or self-reported social functioning. |

## Study 35 | Maran et al. (2026)

| | |
|---|---|
| **Study design** | Cross-sectional case-control and machine-learning study |
| **Setting** | Vall d’Hebron University Hospital, Barcelona, Spain |
| **Population Type (adult, student, specific occupational groups)** | 151 Spanish-speaking adults, including 80 with MDD or persistent depressive disorder and 71 healthy controls |
| **Speaker Role** | Patient, Healthy Control |
| **Elicitation Context** | Controlled |
| **Recording Modality** | Microphone |
| **Sample size** | 151 |
| **Mental health outcome** | Depression |
| **Outcome Biomarker Domain** | Vocal intensity/loudness, Prosody/rhythm, Jitter, Shimmer, Harmonics-to-noise ratio, Formant frequencies, MFCCs, Word or pronoun use, Sentiment/emotional tone, Verbal output, Pitch, Other |
| **Specific variables of marker studied** | RMS energy, F0, intensity, tempo, jitter, shimmer, formants, HNR, MFCCs, TEO features, vocabulary size, word length, nouns, verbs, pronouns, verb tense, repetitions, temporal clusters |

| | |
|---|---|
| **clinical interpretability** | Yes |
| **Perceptial Label (if yes or Partial)** | Lower vocal energy, shorter and simpler speech, smaller vocabulary, more verbs, fewer nouns and fewer past-tense verbs |
| **Outcome Measure/Tool?** | MINI, clinical MDD or PDD diagnosis, MADRS, PHQ-9, GAD-7 |
| **Direction of Association** | Depression was associated with lower RMS energy and multiple TEO and MFCC differences; linguistically, patients used shorter words and sentences, smaller vocabularies, more verbs, fewer nouns, and fewer past-tense verbs |
| **Effect Size/Model Performance/Statistical Significance** | Linguistic model AUC 0.86, accuracy 0.81; acoustic model AUC 0.79, accuracy 0.74; ensemble AUC 0.86, accuracy 0.84, specificity 0.93 |
| **Main Author Conclusions** | Linguistic features outperformed acoustic features, while combining both produced the highest accuracy and specificity for detecting depression |
| **Key Limitations** | Small predominantly female sample, major demographic and medical differences between groups, medication confounding, automated transcripts not manually checked, clinical recording setting, no external validation |
| **Notes** | The strongest linguistic predictors were more verbs, shorter words, smaller vocabulary, fewer nouns and past-tense verbs, and shorter temporal clusters. The ensemble improved specificity but not AUC over the linguistic-only model. |

## Study 36 | Marmar et al. (2019)

| | |
|---|---|
| **Study design** | Case-control machine-learning classification study |
| **Setting** | NYU Langone and affiliated veteran research services, United States |
| **Population Type (adult, student, specific occupational groups)** | 129 male Iraq and Afghanistan warzone-exposed veterans, including 52 with PTSD and 77 controls |
| **Speaker Role** | Patient, Healthy Control |
| **Elicitation Context** | Controlled |
| **Recording Modality** | Microphone |
| **Sample size** | 129 |
| **Mental health outcome** | PTSD |
| **Outcome Biomarker Domain** | Speech rate, Pitch, Vocal intensity/loudness, Prosody/rhythm, MFCCs, Formant frequencies, Other |
| **Specific variables of marker studied** | MFCCs, chroma, pitch, voicing, RMS energy, spectral, articulatory and temporal features |
| **clinical interpretability** | Yes |
| **Perceptial Label (if yes or Partial)** | Slower, more hesitant, monotonous, flatter, and less animated speech |
| **Outcome Measure/Tool?** | Clinician-Administered PTSD Scale, SCID-5, Beck Depression Inventory-II |
| **Direction of Association** | PTSD was associated with slower articulation or longer hesitations, reduced tonal variation, flatter energy patterns, and less activated speech |
| **Effect Size/Model Performance/Statistical Significance** | AUC 0.954, sensitivity 90.4%, specificity 88.3%, overall accuracy 89.1% |

| | |
|---|---|
| **Main Author Conclusions** | An 18-feature speech model accurately distinguished veterans with PTSD from trauma-exposed controls without current MDD |
| **Key Limitations** | Male veteran sample, no independent external validation, emotionally charged PTSD interview, very high-dimensional initial feature set, uncertain generalizability to civilians, women, and comorbid MDD |
| **Notes** | Current MDD was excluded. Depression symptoms, traumatic brain injury, and alcohol use disorder did not substantially improve or confound classification. |

## Study 37 | Mazur et al. (2025)

| | |
|---|---|
| **Study design** | Large cross-sectional machine-learning validation study |
| **Setting** | Remote online recruitment across the United States and Canada |
| **Population Type (adult, student, specific occupational groups)** | 14898 English-speaking adults aged 18 or older recruited through social media |
| **Speaker Role** | Healthy Control |
| **Elicitation Context** | Naturalistic |
| **Recording Modality** | Smartphone, Other |
| **Sample size** | 14898 |
| **Mental health outcome** | Depression |
| **Outcome Biomarker Domain** | Other |
| **Specific variables of marker studied** | Proprietary voice biomarkers extracted from at least 25 seconds of free-form speech |
| **clinical interpretability** | No |
| **Perceptial Label (if yes or Partial)** | |
| **Outcome Measure/Tool?** | Patient Health Questionnaire-9 |
| **Direction of Association** | Specific acoustic marker directions were not reported; the model classified speech as showing depression signals, no depression signals, or requiring further evaluation |
| **Effect Size/Model Performance/Statistical Significance** | Validation sensitivity 71.3%, specificity 73.5%, PPV 69.3%, NPV 75.3%; 20% received an indeterminate result |
| **Main Author Conclusions** | A proprietary voice model showed moderate ability to identify PHQ-9 scores of 10 or higher from brief remotely recorded speech |
| **Key Limitations** | Convenience social-media sample, PHQ-9 rather than clinical diagnosis, proprietary undisclosed features, 20% indeterminate outputs, substantial sex and age performance differences, no medical or psychiatric comorbidity data |
| **Notes** | The prompt was “How was your day?” and recordings lasted 25–75 seconds. Sensitivity was lower in men and adults aged 60 or older, and company employees developed and evaluated the tool. |

## Study 38 | Menne et al. (2024)

| | |
|---|---|
| **Study design** | Cross-sectional case-control and machine-learning study |
| **Setting** | RWTH Aachen University Hospital, Germany |

| | |
|---|---|
| **Population Type (adult, student, specific occupational groups)** | 96 German-speaking adults, including 44 patients with MDD and 52 healthy controls |
| **Speaker Role** | Patient, Healthy Control |
| **Elicitation Context** | Controlled |
| **Recording Modality** | Other |
| **Sample size** | 96 |
| **Mental health outcome** | Depression |
| **Outcome Biomarker Domain** | Speech rate, Pause duration/frequency, Pitch, Vocal intensity/loudness, Verbal output, Voice quality, Jitter, Shimmer, Harmonics-to-noise ratio, Formant frequencies, MFCCs, Word or pronoun use, Sentiment/emotional tone, Other |
| **Specific variables of marker studied** | Pitch slope and range, loudness, pauses, utterance duration, jitter, shimmer, HNR, formants, MFCCs, word count, lexical richness, sentiment |
| **clinical interpretability** | Yes |
| **Perceptial Label (if yes or Partial)** | Flatter and more monotonous pitch, louder and longer speech, fewer pauses relative to speech duration, greater vocal instability, and reduced lexical richness |
| **Outcome Measure/Tool?** | SCID-5, BDI-II, HAMD-21 |
| **Direction of Association** | Compared with controls, patients showed flatter pitch patterns, greater loudness, longer utterances, lower pause rates, more words, reduced lexical richness, and altered sentiment; higher symptom severity correlated with lower minimum pitch and greater jitter and shimmer |
| **Effect Size/Model Performance/Statistical Significance** | Speech model AUC 0.93, balanced accuracy 0.88, sensitivity 0.81, specificity 0.94; mild versus moderate MDD AUC 0.62 |
| **Main Author Conclusions** | Speech features strongly distinguished MDD from controls but performed poorly in separating mild from moderate depression |
| **Key Limitations** | Very small sample, self-reported PTSD classification, secondary dataset analysis, age differed by sex, natural biological sex differences may explain findings, smoking and other vocal confounders were not controlled, no external validation |
| **Notes** | The strongest model used 10 acoustic features. Findings differed by positive versus negative storytelling, and depressed participants unexpectedly spoke louder than controls. |

## Study 39 | Menne et al. (2025)

| | |
|---|---|
| **Study design** | Cross-sectional secondary analysis of an existing conversational dataset |
| **Setting** | USC Institute for Creative Technologies and a veterans' facility in Southern California |
| **Population Type (adult, student, specific occupational groups)** | 31 English-speaking adults with PTSD, including 18 men and 13 women |
| **Speaker Role** | Patient |
| **Elicitation Context** | Controlled |
| **Recording Modality** | Other |
| **Sample size** | 31 |
| **Mental health outcome** | PTSD |

| | |
|---|---|
| **Outcome Biomarker Domain** | Speech rate, Pitch, Vocal intensity/loudness, Harmonics-to-noise ratio, Formant frequencies, Verbal output, Word or pronoun use, Sentiment/emotional tone, Other |
| **Specific variables of marker studied** | F0, F2 variability, HNR, loudness variability, utterance duration, speech ratio, adposition rate, verb-phrase use |
| **clinical interpretability** | Partial |
| **Perceptial Label (if yes or Partial)** | Sex-specific differences in pitch, vocal noisiness, loudness variation, speaking amount, utterance length, and grammatical structure |
| **Outcome Measure/Tool?** | PCL-C, PHQ-8 |
| **Direction of Association** | Males had lower pitch and HNR and greater F2 variability than females; greater PTSD severity was associated with more loudness variation in men, while utterance duration, speech ratio, and grammatical features showed opposite associations by sex |
| **Effect Size/Model Performance/Statistical Significance** | F2 variability CLE=0.816, adjusted p=0.039; HNR CLE=0.081, adjusted p=0.001; F0 CLE=0.073, adjusted p=0.002; male loudness variability ρ=0.66, p<0.01 |
| **Main Author Conclusions** | Speech markers of PTSD severity differed substantially between men and women, supporting sex-specific biomarker development |
| **Key Limitations** | |
| **Notes** | Analyses focused on responses to the “memorable experience” prompt because this was the only question showing a significant sex difference in PTSD severity. |

## Study 40 | Moore et al. (2004)

| | |
|---|---|
| **Study design** | Case-control speech-feature clustering study |
| **Setting** | Medical College of Georgia, Augusta, United States |
| **Population Type (adult, student, specific occupational groups)** | 33 fluent American English-speaking adults, including 15 patients receiving treatment for clinical depression and 18 healthy controls |
| **Speaker Role** | Patient, Healthy Control |
| **Elicitation Context** | Controlled |
| **Recording Modality** | Microphone |
| **Sample size** | 33 |
| **Mental health outcome** | Depression |
| **Outcome Biomarker Domain** | Speech rate, Pitch, Vocal intensity/loudness, Shimmer, Formant frequencies, Voice quality, Other |
| **Specific variables of marker studied** | Pitch, speaking rate, energy variation, formant frequencies and bandwidths, glottal timing, glottal phase ratios, spectral tilt and bias |
| **clinical interpretability** | Partial |
| **Perceptial Label (if yes or Partial)** | Changes in pitch, speaking speed, vocal energy, resonance, and vocal-fold airflow patterns |
| **Outcome Measure/Tool?** | Clinical diagnosis of depression |
| **Direction of Association** | Glottal and vocal-tract features separated depressed participants from controls more consistently than perceptually intuitive pitch or speaking-rate measures |

| | |
|---|---|
| **Effect Size/Model Performance/Statistical Significance** | Male glottal ratio or spectrum accuracy 95.9%–98.7%, female glottal-feature accuracy 89.7%–98.9%; speaking rate showed the weakest accuracy |
| **Main Author Conclusions** | Technical glottal measures provided the most consistent separation of depression and control speech across sexes, even using brief speech segments |
| **Key Limitations** | Very small sample, treated patients, fixed reading task, sex-stratified exploratory analyses, nearly 2000 candidate statistics, observation-level rather than independent subject-level validation, no external validation |
| **Notes** | Participants read a short story for 3–5 minutes. Results suggested that 10–20 seconds of speech could classify observations accurately, but the authors stated that a larger corpus was needed for clinical application. |

## Study 41 | Mundt et al. (2012)

| | |
|---|---|
| **Study design** | Multisite randomized double-blind placebo-controlled longitudinal trial |
| **Setting** | Eleven clinical research sites across the United States |
| **Population Type (adult, student, specific occupational groups)** | 105 adults aged 18–65 with major depressive disorder completing a 4-week sertraline or placebo trial |
| **Speaker Role** | Patient |
| **Elicitation Context** | Controlled |
| **Recording Modality** | Telephone |
| **Sample size** | 105 |
| **Mental health outcome** | Depression |
| **Outcome Biomarker Domain** | Speech rate, Pause duration/frequency, Pitch, Verbal output, Formant frequencies, Other |
| **Specific variables of marker studied** | Recording time, vocalization time, number and duration of pauses, pause variability, percent pause time, speech-to-pause ratio, speaking rate, F0, F1 and F2 |
| **clinical interpretability** | Yes |
| **Perceptial Label (if yes or Partial)** | Slower speech with longer, more frequent and more variable pauses, improving toward faster and less paused speech with treatment |
| **Outcome Measure/Tool?** | DSM-IV MDD diagnosis, QIDS-C, QIDS-SR, HAM-D-17 |
| **Direction of Association** | Greater depression severity was associated with longer recordings, more pause time and variability, lower speech-to-pause ratios, and slower speaking; treatment responders showed larger reductions in pausing and increased speaking rate |
| **Effect Size/Model Performance/Statistical Significance** | Change-based response model sensitivity 70.6%, specificity 79.2%; baseline model sensitivity 62.7%, specificity 66.7%; prior model positive predictive value 89% |
| **Main Author Conclusions** | Telephone-derived speech timing measures replicated prior findings and tracked depression severity and treatment response across active treatment and placebo groups |
| **Key Limitations** | Only 105 of 165 randomized participants were analyzable, short 4-week trial, telephone audio quality, extensive exclusions reduced generalizability, exploratory biomarker objective, some frequency findings were not replicated, newly fitted models lacked external validation |
| **Notes** | Speech was collected through free speech, alphabet and counting tasks, passage reading, and sustained vowels. Timing markers were more consistent than pitch and formant findings. |

## Study 42 | Nik Nur Wahidah et al. (2015)

| | |
|---|---|
| **Study design** | Case-control classification and regression study with cross-dataset testing |
| **Setting** | Vanderbilt Emergency Department and Psychiatric Hospital, Tennessee, United States |
| **Population Type (adult, student, specific occupational groups)** | 66 psychiatric patients across a development sample of 47 high-risk suicidal or depressed patients and a separate test sample of 19 high-risk suicidal patients |
| **Speaker Role** | Patient |
| **Elicitation Context** | Controlled |
| **Recording Modality** | Microphone |
| **Sample size** | 66 |
| **Mental health outcome** | Depression, Other |
| **Outcome Biomarker Domain** | Speech rate, Pause duration/frequency, Verbal output, MFCCs, Other |
| **Specific variables of marker studied** | Voiced, unvoiced and silent interval lengths, transitions between speech and silence, vowel duration, PSD bands, MFCCs |
| **clinical interpretability** | Yes |
| **Perceptial Label (if yes or Partial)** | Slower speech, longer pauses, prolonged vowels, and fewer transitions between speech and silence |
| **Outcome Measure/Tool?** | Clinician-rated HAMD, BDI-II, MINI, Pierce Suicide Intent Scale |
| **Direction of Association** | High-risk suicidal speech generally contained longer silent and voiced intervals than depressed speech, indicating longer pauses, slower speech, and prolonged vowels; timing features also predicted depression severity |
| **Effect Size/Model Performance/Statistical Significance** | Female leave-one-out classification reached 79%; male timing features detected 86%–100% of high-risk cases in an external dataset; HAMD prediction MAE was approximately 1.3–2.2 points |
| **Main Author Conclusions** | Timing patterns distinguished near-term suicidal risk from depression and predicted HAMD scores, with greater robustness across recording devices than spectral features |
| **Key Limitations** | Very small sex-stratified samples, reading task only, HAMD available for only 23 patients, unclear diagnostic thresholds, feature-selection and overfitting risk, female findings did not generalize well across datasets, no independent prospective clinical validation |
| **Notes** | Near-term suicidal risk was coded Other. Male cross-dataset performance was strong, but the best female classifier identified only 58% of high-risk cases in the second dataset. |

## Study 43 | Ooba et al. (2025)

| | |
|---|---|
| **Study design** | Cross-sectional deep-learning classification study |
| **Setting** | Okayama University Hospital, Japan |
| **Population Type (adult, student, specific occupational groups)** | 172 postpartum women, including 23 with documented mental disorders and 149 without a psychiatric diagnosis |
| **Speaker Role** | Patient, Healthy Control |

| | |
|---|---|
| **Elicitation Context** | Naturalistic |
| **Recording Modality** | Microphone |
| **Sample size** | 172 |
| **Mental health outcome** | Depression, Anxiety, Bipolar/Mania, Other |
| **Outcome Biomarker Domain** | Other |
| **Specific variables of marker studied** | Mel-spectrogram representations of 5-second conversational audio segments |
| **clinical interpretability** | No |
| **Perceptial Label (if yes or Partial)** | |
| **Outcome Measure/Tool?** | Documented DSM-5 psychiatric diagnosis, Edinburgh Postnatal Depression Scale |
| **Direction of Association** | The model detected the presence of any documented mental disorder, but individual acoustic marker directions were not reported |
| **Effect Size/Model Performance/Statistical Significance** | Voice model sensitivity 1.00, specificity 0.65, accuracy 0.70, F1 0.63, ROC-AUC 0.82; EPDS sensitivity 0.50, specificity 0.97, ROC-AUC 0.74 |
| **Main Author Conclusions** | A deep-learning voice model showed high sensitivity for identifying postpartum women with diverse mental disorders, but produced many false positives |
| **Key Limitations** | Small single-center sample, only 23 affected participants and 6 affected test cases, heterogeneous diagnoses, diagnoses predated delivery, no assessment of undiagnosed controls, no cross-validation, data augmentation may introduce artificial patterns, black-box model |
| **Notes** | The positive class included MDD, bipolar disorder, adjustment disorder, anxiety, schizophrenia, intellectual disability, and other conditions. Ambient consultation-room audio was manually cleaned before analysis. |

## Study 44 | Pan et al. (2023)

| | |
|---|---|
| **Study design** | Cross-sectional multiclass machine-learning classification study |
| **Setting** | Clinical and research settings in China |
| **Population Type (adult, student, specific occupational groups)** | 272 Chinese adults, including 106 with depression, 32 with bipolar disorder, 20 with schizophrenia, and 114 healthy controls |
| **Speaker Role** | Patient, Healthy Control |
| **Elicitation Context** | Controlled |
| **Recording Modality** | Microphone |
| **Sample size** | 272 |
| **Mental health outcome** | Depression, Bipolar/Mania, Other |
| **Outcome Biomarker Domain** | MFCCs |
| **Specific variables of marker studied** | Thirteen MFCCs and their derivatives summarized as i-vector representations |
| **clinical interpretability** | No |

| | |
|---|---|
| **Perceptial Label (if yes or Partial)** | |
| **Outcome Measure/Tool?** | DSM-IV psychiatric diagnosis by psychiatrists |
| **Direction of Association** | Individual acoustic directions were not reported; MFCC-derived voice patterns distinguished most clinical groups, but depression and euthymic bipolar disorder were not separable |
| **Effect Size/Model Performance/Statistical Significance** | Depression versus healthy controls AUC 0.77, depression versus schizophrenia AUC 0.83, depression versus bipolar disorder AUC 0.50, bipolar disorder versus schizophrenia AUC 0.92 |
| **Main Author Conclusions** | MFCC-derived vocal patterns showed potential for psychiatric differential diagnosis but could not distinguish depression from euthymic bipolar disorder |
| **Key Limitations** | Small bipolar and schizophrenia groups, Chinese-only sample, controlled tasks, bipolar patients were euthymic and schizophrenia patients were in remission, no anxiety group, no external validation, technical features lacked clinical interpretability |
| **Notes** | Participants completed video-description, reading, question-answering, and picture-description tasks under positive, negative, and neutral emotional conditions. Schizophrenia was coded Other. |

## Study 45 | Quatieri et al. (2024)

| | |
|---|---|
| **Study design** | Cross-sectional machine-learning screening study using an existing longitudinal cohort |
| **Setting** | Walter Reed National Military Medical Center and affiliated US military research settings |
| **Population Type (adult, student, specific occupational groups)** | 141 adults with no TBI or mild TBI who completed reading or free-speech tasks and had varying PTSD symptom severity |
| **Speaker Role** | Patient, Healthy Control |
| **Elicitation Context** | Controlled |
| **Recording Modality** | Microphone |
| **Sample size** | 141 |
| **Mental health outcome** | PTSD |
| **Outcome Biomarker Domain** | Pitch, Vocal intensity/loudness, Voice quality, MFCCs, Sentiment/emotional tone, Other |
| **Specific variables of marker studied** | Speech energy, F0, cepstral peak prominence, MFCCs, arousal and valence estimates, respiratory, phonatory and articulatory coordination features |
| **clinical interpretability** | Partial |
| **Perceptial Label (if yes or Partial)** | Altered coordination of loudness, pitch, vocal stability, and articulation during calmer or more positive speech |
| **Outcome Measure/Tool?** | PCL-C |
| **Direction of Association** | Individual acoustic directions were not reported; speech regions with low arousal and positive valence provided the strongest PTSD discrimination, and combining respiratory, phonatory, and articulatory features performed best |
| **Effect Size/Model Performance/Statistical Significance** | Emotion-filtered model AUC 0.80 versus 0.68 without filtering and 0.70 with high-arousal negative-valence filtering; approximately 80% detection at a 25% false-positive rate |
| **Main Author Conclusions** | Selecting calmer and more positively valenced speech segments improved voice-based PTSD screening compared with analyzing the full recording |

| | |
|---|---|
| **Key Limitations** | Military and TBI-enriched sample, PTSD defined by self-reported PCL-C threshold, MDD was not excluded, limited female representation, technical fused features, no independent external validation, unclear specificity against related disorders |
| **Notes** | Participants completed picture-story retelling or free speech and Caterpillar passage reading. The model used a PCL-C cutoff above 35, yielding 33 positive cases. |

## Study 46 | Regondi et al. (2025)

| | |
|---|---|
| **Study design** | Exploratory cross-sectional deep-learning classification study |
| **Setting** | RAM Psiche psychotherapy clinic and research centres in Milan and Rome, Italy |
| **Population Type (adult, student, specific occupational groups)** | Five Italian psychotherapy patients, including two with MDD and three with GAD, contributing seven recorded sessions and over 1000 speech segments |
| **Speaker Role** | Patient |
| **Elicitation Context** | Naturalistic |
| **Recording Modality** | Smartphone, Microphone, Other |
| **Sample size** | 5 |
| **Mental health outcome** | Depression, Anxiety |
| **Outcome Biomarker Domain** | Speech rate, Pause duration/frequency, Pitch, Vocal intensity/loudness, Jitter, Shimmer, Formant frequencies, Word or pronoun use, Verbal output, Other |
| **Specific variables of marker studied** | F0 mean and variability, pitch slope and range, loudness mean and range, pause rate, jitter, shimmer, F1, F2, lexical richness, word rate, pronouns, nouns, adverbs, lexical density, disorder-related keywords, Mel spectrograms |
| **clinical interpretability** | Yes |
| **Perceptial Label (if yes or Partial)** | Lower and flatter pitch, quieter speech, more pauses, vocal tremor, and altered lexical richness and adverb use |
| **Outcome Measure/Tool?** | DSM-5 clinical diagnosis, psychologist-applied DASS-21 symptom labeling |
| **Direction of Association** | Depression was associated especially with lower pitch, reduced intensity, and increased pausing, while lexical richness and adverb use contributed to both depression and anxiety classification |
| **Effect Size/Model Performance/Statistical Significance** | VOM 1.1 F1=0.70, depression detection 71%, anxiety 48%, no pathology 74%; VOM 1.3 F1=0.78, depression 77%, anxiety 74%, no pathology 78% |
| **Main Author Conclusions** | A hybrid acoustic and lexical model distinguished depression, anxiety, and no-pathology speech segments and generalized moderately well to external neutral voices |
| **Key Limitations** | Only five patients, segment-level rather than participant-level evaluation, one psychologist provided labels, pseudo-labeling and augmentation may introduce bias, no independent clinical validation, possible speaker leakage, heterogeneous recording devices and no loudness calibration |
| **Notes** | The study analyzed symptom-positive moments within psychotherapy sessions rather than classifying each entire patient recording. External neutral voices improved recognition of the no-pathology class. |

## Study 47 | Riad et al. (2024)

| | |
|---|---|
| **Study design** | Cross-sectional algorithm-development and validation study with held-out testing |
| **Setting** | Remote home-based smartphone study in France |

| | |
|---|---|
| **Population Type (adult, student, specific occupational groups)** | 865 French adults from the general population without self-reported severe psychiatric, neurological, or speech disorders |
| **Speaker Role** | Healthy Control |
| **Elicitation Context** | Naturalistic |
| **Recording Modality** | Smartphone |
| **Sample size** | 865 |
| **Mental health outcome** | Depression, Anxiety, Fatigue, Other |
| **Outcome Biomarker Domain** | Other |
| **Specific variables of marker studied** | Pretrained Whisper speech embeddings extracted from spontaneous speech, with max pooling and linear machine-learning models |
| **clinical interpretability** | No |
| **Perceptial Label (if yes or Partial)** | |
| **Outcome Measure/Tool?** | PHQ-9, Beck Depression Inventory, GAD-7, Athens Insomnia Scale, Multidimensional Fatigue Inventory |
| **Direction of Association** | Individual speech-marker directions were not reported; latent speech embeddings predicted clinical thresholds and symptom severity across depression, anxiety, insomnia, and fatigue |
| **Effect Size/Model Performance/Statistical Significance** | Depression AUC 0.76 by PHQ-9 and 0.78 by BDI, anxiety AUC 0.77, insomnia AUC 0.73, total fatigue AUC 0.68; severity correlations r=0.31–0.49 |
| **Main Author Conclusions** | Brief smartphone-recorded speech showed moderate performance for detecting and estimating several co-occurring mental health symptoms in the general population |
| **Key Limitations** | French-only convenience sample, self-report outcomes without clinical diagnoses, cross-sectional design, fixed train-validation-test split, latent black-box features, poor fairness across some age and education groups, company involvement |
| **Notes** | Participants answered one prompt about their current feelings and recent sleep. The model could abstain when uncertain and was more consistent across sex than across age or education. |

## Study 48 | Tan et al. (2016)

| | |
|---|---|
| **Study design** | Cross-sectional correlation and regression prototype study |
| **Setting** | Secondary analysis of the AVEC depression database |
| **Population Type (adult, student, specific occupational groups)** | 47 adult women with depression who produced a sustained vowel recording |
| **Speaker Role** | Patient |
| **Elicitation Context** | Controlled |
| **Recording Modality** | Microphone |
| **Sample size** | 47 |
| **Mental health outcome** | Depression |

| | |
|---|---|
| **Outcome Biomarker Domain** | Jitter, Formant frequencies |
| **Specific variables of marker studied** | Jitter, F1, F2 and F3 measured during sustained /■/ phonation |
| **clinical interpretability** | Partial |
| **Perceptial Label (if yes or Partial)** | Greater vocal instability and altered vocal-tract resonance with increasing depression severity |
| **Outcome Measure/Tool?** | Beck Depression Inventory |
| **Direction of Association** | Higher BDI scores were associated with increased jitter, F1 and F2, while F3 showed little association |
| **Effect Size/Model Performance/Statistical Significance** | Jitter r=0.47, F1 r=0.37, F2 r=0.32, F3 r=-0.14; BDI prediction MAE=8.73 and RMSE=9.67 |
| **Main Author Conclusions** | Jitter and lower formant measures showed modest associations with depression severity, but the resulting smartphone prototype had limited predictive accuracy |
| **Key Limitations** | Very small female-only sample, no healthy controls, secondary database analysis, sustained vowel only, same-source development and test samples, three-predictor model, no independent or clinical validation |
| **Notes** | The prototype required users to extract jitter and formants externally with Praat and manually enter them into the app, limiting automation and practical use. |

## Study 49 | Shin et al. (2021)

| | |
|---|---|
| **Study design** | Cross-sectional observational and machine-learning classification study |
| **Setting** | Seoul National University Hospital, South Korea |
| **Population Type (adult, student, specific occupational groups)** | 93 adults, including 33 without a current depressive episode, 26 with a minor depressive episode, and 34 with a major depressive episode |
| **Speaker Role** | Patient, Healthy Control |
| **Elicitation Context** | Controlled |
| **Recording Modality** | Microphone |
| **Sample size** | 93 |
| **Mental health outcome** | Depression |
| **Outcome Biomarker Domain** | Pitch, Vocal intensity/loudness, Verbal output, Formant frequencies, Other |
| **Specific variables of marker studied** | Spectral centroid, spectral roll-off, F2 bandwidth, mean pitch, pitch variability, zero-crossing rate, voice portion |
| **clinical interpretability** | Partial |
| **Perceptial Label (if yes or Partial)** | Lower-pitched speech, greater pitch variation, and altered speaking amount |
| **Outcome Measure/Tool?** | MINI, Hamilton Depression Rating Scale, PHQ-9 |
| **Direction of Association** | Compared with the nondepressed group, both depressive groups generally had lower spectral and pitch measures, greater pitch variability, and a higher proportion of voiced speech, although changes were not consistently ordered by depression severity |

| | |
|---|---|
| **Effect Size/Model Performance/Statistical Significance** | Best three-group MLP model: mean accuracy 60.0%, AUC 0.659, sensitivity 65.6%, specificity 66.2%; minor-depression AUC 0.69–0.79 and major-depression AUC 0.58–0.67 |
| **Main Author Conclusions** | Voice features showed modest ability to distinguish no, minor, and major depressive episodes, with minor depression showing substantial vocal differences from controls |
| **Key Limitations** | Small single-site sample, many participants had bipolar disorder and used antipsychotics, anxiety may have influenced voice, cross-sectional design, artificial data augmentation, repeated train-test splits without external validation, unclear generalizability |
| **Notes** | The groups reflected current depressive episode severity rather than diagnosis. Most participants in the minor and major episode groups had bipolar disorder, and vocal changes were sometimes greater in minor than major depression. |

## Study 50 | Sigmund (2010)

| | |
|---|---|
| **Study design** | Within-subject observational speech-analysis study |
| **Setting** | Brno University of Technology oral examinations, Czech Republic |
| **Population Type (adult, student, specific occupational groups)** | 31 male undergraduate and postgraduate students recorded during examination stress and a later neutral condition |
| **Speaker Role** | Healthy Control |
| **Elicitation Context** | Naturalistic, Controlled |
| **Recording Modality** | Microphone |
| **Sample size** | 31 |
| **Mental health outcome** | Stress |
| **Outcome Biomarker Domain** | Pitch, Formant frequencies, Other |
| **Specific variables of marker studied** | Mean F0, F0 variability, short-time vowel frequency spectra and spectral-envelope modulation |
| **clinical interpretability** | Yes |
| **Perceptial Label (if yes or Partial)** | Higher-pitched and more variable speech with altered vowel resonance during examination stress |
| **Outcome Measure/Tool?** | Examination condition, with heart rate measured in a subset |
| **Direction of Association** | All examined speakers showed higher mean F0 and generally wider F0 distributions under stress; vowel spectra, especially /e/ and /i/, showed greater harmonic modulation around 2–3 kHz |
| **Effect Size/Model Performance/Statistical Significance** | Descriptive analysis only, no classifier; stressed mean F0 exceeded neutral mean F0 in all 10 speakers shown |
| **Main Author Conclusions** | Psychological examination stress consistently increased pitch and altered vowel spectra, supporting speech as a potential stress indicator |
| **Key Limitations** | Male-only student sample, small exploratory analysis, unclear number included in each spectral analysis, no validated psychological stress scale, limited statistical testing, no predictive model or external validation |
| **Notes** | The ExamStress database included spontaneous examination speech, pre-examination reading, and neutral reading recorded several days later. Heart rate supported the presence of examination stress. |

## Study 51 | Silva et al. (2024)

| | |
|---|---|
| **Study design** | Observational cross-sectional case-control study |
| **Setting** | Psychosocial care and complementary practice centres in Paraíba, Brazil |
| **Population Type (adult, student, specific occupational groups)** | 144 Brazilian adults, including 54 with clinically diagnosed depression and 90 controls without psychiatric disorders |
| **Speaker Role** | Patient, Healthy Control |
| **Elicitation Context** | Controlled |
| **Recording Modality** | Microphone |
| **Sample size** | 144 |
| **Mental health outcome** | Depression |
| **Outcome Biomarker Domain** | Pitch, Voice quality, Jitter, Shimmer, Other |
| **Specific variables of marker studied** | F0 mean and variability, jitter, shimmer, glottal-to-noise excitation ratio, cepstral peak prominence-smoothed, spectral tilt |
| **clinical interpretability** | Partial |
| **Perceptial Label (if yes or Partial)** | More unstable, rough, breathy, and less periodic voice quality |
| **Outcome Measure/Tool?** | ICD-10 clinical diagnosis, BDI-II, SRQ-20, Voice Symptom Scale |
| **Direction of Association** | Depressed participants had greater F0 variability, jitter, and shimmer and lower GNE, CPPS, and spectral tilt; mean F0 did not differ |
| **Effect Size/Model Performance/Statistical Significance** | Jitter and CPPS independently predicted BDI-II scores, with model $R^2=0.508$ and adjusted $R^2=0.498$ |
| **Main Author Conclusions** | Acoustic voice-quality measures from a sustained vowel distinguished depressed participants from controls and explained about half of the variation in depression scores |
| **Key Limitations** | Convenience sample, demographic differences between groups, most patients used psychotropic medication, cross-sectional design, sustained vowel only, no independent validation, regression used BDI-II rather than blinded diagnostic outcome |
| **Notes** | Participants sustained the vowel /e/ for analysis. Antidepressant use was associated with CPPS, and the study did not use machine-learning classification. |

## Study 52 | Stasak et al. (2022)

| | |
|---|---|
| **Study design** | Cross-sectional machine-learning classification study with age-stratified modeling |
| **Setting** | Remote smartphone recordings collected in participants' homes |
| **Population Type (adult, student, specific occupational groups)** | 152 adults aged 18–79, evenly divided across four age groups, including 44 above the PHQ-9 depression threshold |
| **Speaker Role** | Healthy Control |
| **Elicitation Context** | Naturalistic, Controlled |
| **Recording Modality** | Smartphone |

| | |
|---|---|
| **Sample size** | 152 |
| **Mental health outcome** | Depression |
| **Outcome Biomarker Domain** | Pitch, Verbal output, Voice quality, MFCCs, Prosody/rhythm, Other |
| **Specific variables of marker studied** | F0 and voiced-frame measures, glottal features, MFCCs and spectral features, articulatory landmark timing, roughness, breathiness, asthenia, strain, instability |
| **clinical interpretability** | Yes |
| **Perceptial Label (if yes or Partial)** | Lower pitch variation and poorer, rougher, breathier, weaker, strained, and less stable voice quality |
| **Outcome Measure/Tool?** | PHQ-9 |
| **Direction of Association** | Depressed participants generally showed poorer and more complex voice-quality abnormalities across age groups; depressed older adults had particularly low F0 variability, while feature patterns differed substantially by age |
| **Effect Size/Model Performance/Statistical Significance** | Age-dependent accuracy was 68% for glottal, 66% for prosodic, 73% for spectral, 74% for landmark, and 75% for voice-quality features, improving up to 10 percentage points over age-agnostic models |
| **Main Author Conclusions** | Accounting for age improved smartphone-based depression detection and increased identification of depressed speakers, especially among older adults |
| **Key Limitations** | Small age-stratified groups, PHQ-9 rather than clinical diagnosis, one brief constrained speech task, private dataset, manually rated voice quality, no independent external validation, company involvement |
| **Notes** | Participants repeated “pataka” for about 6 seconds. Each age group contained 38 speakers, and age-dependent models generally outperformed age-agnostic and gender-dependent models. |

## Study 53 | Stasak et al. (2019)

| | |
|---|---|
| **Study design** | Cross-sectional case-control and machine-learning classification study |
| **Setting** | Black Dog Institute clinical setting in Sydney, Australia |
| **Population Type (adult, student, specific occupational groups)** | 70 adults aged 21–75, including 35 with a current major depressive episode and 35 nondepressed controls |
| **Speaker Role** | Patient, Healthy Control |
| **Elicitation Context** | Controlled |
| **Recording Modality** | Microphone |
| **Sample size** | 70 |
| **Mental health outcome** | Depression |
| **Outcome Biomarker Domain** | Pause duration/frequency, Pitch, Vocal intensity/loudness, Prosody/rhythm, Voice quality, MFCCs, Verbal output, Other |
| **Specific variables of marker studied** | Hesitations, word and phrase repetitions, speech errors, self-corrections, voiced and unvoiced duration, F0, loudness, formants, MFCCs |
| **clinical interpretability** | Yes |

| | |
|---|---|
| **Perceptial Label (if yes or Partial)** | More hesitations, pauses, repetitions and pronunciation errors, longer speech duration, and less variable pitch and loudness |
| **Outcome Measure/Tool?** | MINI, QIDS-SR |
| **Direction of Association** | Depressed speakers had about 55% more hesitations and 71% more speech errors, longer pauses and more unvoiced speech, and reduced pitch and loudness variability |
| **Effect Size/Model Performance/Statistical Significance** | Baseline eGeMAPS accuracy 65%; speech-error accuracy 71%; neutral-sentence hesitation accuracy 86%; selected disfluency combinations reached 83% |
| **Main Author Conclusions** | Disfluency and timing features from affective sentence reading outperformed broad acoustic features, particularly when sentence valence and keyword position were considered |
| **Key Limitations** | Small single-site sample, only clearly nondepressed or moderate-to-severe depression groups, controlled reading task, manual annotation, repeated cross-validation without external validation, limited generalizability to spontaneous speech |
| **Notes** | The task used 20 affective sentences. Depressed participants made more errors but also attempted more self-corrections, and positive or neutral sentences generally performed better than negative sentences. |

## Study 54 | Taguchi et al. (2018)

| | |
|---|---|
| **Study design** | Cross-sectional case-control discrimination study |
| **Setting** | University and psychiatric clinical sites in Japan |
| **Population Type (adult, student, specific occupational groups)** | 72 Japanese adults, including 36 patients with major depressive disorder and 36 healthy controls |
| **Speaker Role** | Patient, Healthy Control |
| **Elicitation Context** | Controlled |
| **Recording Modality** | Smartphone, Other |
| **Sample size** | 72 |
| **Mental health outcome** | Depression |
| **Outcome Biomarker Domain** | MFCCs, Pitch, Vocal intensity/loudness, Harmonics-to-noise ratio, Other |
| **Specific variables of marker studied** | MFCC1–12, RMS energy, zero-crossing rate, HNR, fundamental frequency |
| **clinical interpretability** | Partial |
| **Perceptial Label (if yes or Partial)** | More muffled speech with reduced energy in the 2000–3000 Hz frequency range |
| **Outcome Measure/Tool?** | DSM-5 clinical diagnosis, QIDS-SR-J |
| **Direction of Association** | MFCC 2 was higher in patients with depression and reflected lower spectral energy around 2000–3000 Hz; other acoustic features did not differ and MFCC 2 was not associated with symptom severity |
| **Effect Size/Model Performance/Statistical Significance** | MFCC 2 sensitivity 77.8%, specificity 86.1%, accuracy 81.9%, AUC 0.88 |
| **Main Author Conclusions** | A single MFCC feature distinguished MDD from controls with good accuracy and may reflect the clinically perceived muffled quality of depressed speech |

| | |
|---|---|
| **Key Limitations** | Small sample, nearly all patients used psychotropic medication, brief digit-reading task, no external validation, possible recording noise, feature did not correlate with depression severity |
| **Notes** | Participants read a 10-digit sequence before and after a verbal-fluency task. MFCC 2 remained different between groups regardless of age or sex. |

## Study 55 | Teferra et al. (2022)

| | |
|---|---|
| **Study design** | Cross-sectional web-based validation study |
| **Setting** | Remote online study using participants' home computers |
| **Population Type (adult, student, specific occupational groups)** | 1744 English-speaking adults aged 18–65 from a nonclinical online sample, including 540 above the GAD-7 screening threshold |
| **Speaker Role** | Healthy Control |
| **Elicitation Context** | Controlled |
| **Recording Modality** | Other |
| **Sample size** | 1744 |
| **Mental health outcome** | Anxiety |
| **Outcome Biomarker Domain** | Pause duration/frequency, Pitch, Vocal intensity/loudness, Prosody/rhythm, Voice quality, MFCCs, Verbal output, Formant frequencies, Other |
| **Specific variables of marker studied** | Speaking duration, word count, articulation rate, F0, intensity, jitter, shimmer, F1–F3, MFCCs, LPCCs, linguistic word categories |
| **clinical interpretability** | Yes |
| **Perceptial Label (if yes or Partial)** | Less speech, shorter sentences, greater vocal instability, and more negative, anxious, angry, and sad language |
| **Outcome Measure/Tool?** | GAD-7 |
| **Direction of Association** | Higher anxiety scores were associated with shorter speaking duration, fewer words, greater shimmer, altered pitch variability and technical cepstral measures, and greater use of negative-emotion language; several linguistic associations differed by sex |
| **Effect Size/Model Performance/Statistical Significance** | Speaking duration and word count r=-0.12, negative-emotion words r=0.10, shimmer r=0.08; most acoustic correlations were small, with no prediction model reported |
| **Main Author Conclusions** | Speech quantity, vocal characteristics, and linguistic content showed small but significant associations with anxiety severity in a large remote sample |
| **Key Limitations** | Self-reported anxiety without clinical diagnosis, cross-sectional design, many uncorrected statistical tests, small effect sizes, variable home microphones and environments, online selection bias, substantial participant dropout |
| **Notes** | The modified Trier Social Stress Test asked participants to deliver a five-minute impromptu job-interview speech. Jitter, articulation rate, F2, F3, and several previously proposed features were not significantly associated with anxiety. |

## Study 56 | Tonn et al. (2022)

| | |
|---|---|
| **Study design** | Proof-of-concept longitudinal evaluation study with machine-learning validation |
| **Setting** | Neuropsychiatric Center of Hamburg, Germany |

| | |
|---|---|
| **Population Type (adult, student, specific occupational groups)** | 163 German-speaking psychiatric outpatients aged 15–82, contributing 292 assessment sessions across varying depressive states |
| **Speaker Role** | Patient |
| **Elicitation Context** | Naturalistic |
| **Recording Modality** | Smartphone, Other |
| **Sample size** | 163 |
| **Mental health outcome** | Depression |
| **Outcome Biomarker Domain** | Pitch, Vocal intensity/loudness, Pause duration/frequency, Prosody/rhythm, Other |
| **Specific variables of marker studied** | Proprietary set of pitch, amplitude, silence, duration, range, slope, frequency, and shape parameters |
| **clinical interpretability** | No |
| **Perceptial Label (if yes or Partial)** | |
| **Outcome Measure/Tool?** | PHQ-9, ICD-10 psychiatric diagnosis |
| **Direction of Association** | Specific marker directions were not reported; the proprietary vocal depression score increased across PHQ-9 severity categories and correlated moderately with PHQ-9 scores |
| **Effect Size/Model Performance/Statistical Significance** | Test-set accuracy 68.0%, sensitivity 59.7%, specificity 72.6%, precision 54.5%; overall correlation with PHQ-9 r=0.41 |
| **Main Author Conclusions** | The content-free voice model showed moderate agreement with PHQ-9 depression severity and potential for remote screening and monitoring |
| **Key Limitations** | Proprietary undisclosed features, PHQ-9 used as the primary reference, repeated sessions treated as independent observations, modest test performance, single outpatient center, predominantly female sample, no external validation, company funding and involvement |
| **Notes** | Participants provided at least two minutes of spontaneous speech in response to general questions. The model selected 13 weighted parameters from more than 200 acoustic measures. |

## Study 57 | Verde et al. (2021)

| | |
|---|---|
| **Study design** | Cross-sectional case-control machine-learning classification study |
| **Setting** | Mental health and psychological centres in Caserta and Aversa, Italy |
| **Population Type (adult, student, specific occupational groups)** | 149 Italian adults, including 87 with depression and 62 healthy controls |
| **Speaker Role** | Patient, Healthy Control |
| **Elicitation Context** | Naturalistic |
| **Recording Modality** | Microphone |
| **Sample size** | 149 |
| **Mental health outcome** | Depression |

| | |
|---|---|
| **Outcome Biomarker Domain** | Jitter, MFCCs, Other |
| **Specific variables of marker studied** | Jitter, 13 MFCCs, MFCC derivatives, spectral centroid |
| **clinical interpretability** | No |
| **Perceptial Label (if yes or Partial)** | |
| **Outcome Measure/Tool?** | Clinical psychiatric assessment, BDI-II |
| **Direction of Association** | Individual marker directions were not reported; a reduced set of acoustic features from spontaneous speech distinguished depressed participants from healthy controls |
| **Effect Size/Model Performance/Statistical Significance** | SVM accuracy 85.23%, sensitivity 86.21%, specificity 83.87%, precision 88.24%, F1=87.21%, AUC 0.85 |
| **Main Author Conclusions** | A lightweight SVM using a small acoustic feature set classified depression with good performance and required relatively low computational resources |
| **Key Limitations** | Private single-country dataset, modest sample size, only the first 10 seconds of each recording analyzed, no independent external validation, no feature-level interpretation, unclear demographic and medication details, possible speaker and recording confounding |
| **Notes** | Participants described their activities during the previous week. The best model used a polynomial-kernel SVM with 10-fold cross-validation. |

## Study 58 | Vilenchik et al. (2025)

| | |
|---|---|
| **Study design** | Cross-sectional machine-learning classification study |
| **Setting** | Outpatient chronic pain clinic adjacent to Soroka University Medical Center, Beer Sheva, Israel |
| **Population Type (adult, student, specific occupational groups)** | 143 adults attending a chronic pain clinic (123 women, 20 men), mean age 52.3 years, of whom 62.5% met CES-D criteria for depression |
| **Speaker Role** | Patient |
| **Elicitation Context** | Controlled |
| **Recording Modality** | Other |
| **Sample size** | 143 |
| **Mental health outcome** | Depression |
| **Outcome Biomarker Domain** | Pitch, Vocal intensity/loudness, Voice quality, Jitter, Shimmer, Harmonics-to-noise ratio, Formant frequencies, MFCCs, Other |
| **Specific variables of marker studied** | F0, loudness, equivalent sound level, formant amplitude and bandwidth, spectral slope, Hammarberg index, spectral flux, HNR, jitter, shimmer and MFCCs, drawn from the eGeMAPS 88-feature set and the OpenSMILE EmoLarge 6552-feature set; Wav2Vec embeddings used in a separate projection analysis |
| **clinical interpretability** | Partial |
| **Perceptial Label (if yes or Partial)** | Loudness, pitch and voice quality changes, although the highest-ranked model features were technical spectral and cepstral measures without perceptual equivalents |
| **Outcome Measure/Tool?** | CES-D short form at its validated clinical cut-off, administered in person by trained interviewers; PHQ-15 for somatic symptom severity; single-item SUDs distress rating used as a model input rather than as the label |

| | |
|---|---|
| **Direction of Association** | Individual marker directions were not reported; SHAP analysis indicated that greater formant amplitude variability and greater spectral slope variability increased the predicted probability of depression |
| **Effect Size/Model Performance/Statistical Significance** | Whole-sample voice-only accuracy 66.6% against a 64.2% baseline, a 2% lift within one fold standard deviation. Enriched low/high-SUDs female subgroup (n=52) accuracy 65.3% (SD 2.8) against a 50% baseline, a 15.3% lift, p=0.01. SUDs alone 86%; SUDs combined with acoustic features 92.4%, a 6.4% increment at p=0.1 |
| **Main Author Conclusions** | A high-prevalence, carefully labelled clinical dataset can support above-chance depression detection with a small sample, and combining acoustic features with a single distress item improves accuracy beyond either alone |
| **Key Limitations** | Whole-sample voice-only lift was approximately 2% and within one fold standard deviation; the headline 15.3% lift came from a subgroup enriched by selecting women at the extremes of a single distress item; sample was 86% female; 67.5% had severe somatic symptoms on PHQ-15 and pain confounding was addressed by theoretical argument rather than empirically; internal k-fold cross-validation only with no external validation; 10-second chunk augmentation produced correlated observations within participants; the 92% figure depends on a self-report item rather than on voice |
| **Notes** | Published 2025 but not retrieved in the original search; identified during peer review and added following a verification search - confirm retrieval status in Covidence and report the updated search date in the Methods. Wav2Vec PCA showed that the dominant variance in speech embeddings was speaker sex and speaker identity rather than depression, giving a direct mechanistic demonstration of why between-person models fail. The 92% figure is not a voice-only result: SUDs alone gave 86% and the acoustic increment did not reach conventional significance. |

## Study 59 | Wadle et al. (2024)

| | |
|---|---|
| **Study design** | Intensive longitudinal ambulatory assessment pilot study |
| **Setting** | Central Institute of Mental Health inpatient setting in Mannheim, Germany |
| **Population Type (adult, student, specific occupational groups)** | 22 adults aged 18–63 with acute depressive episodes undergoing sleep deprivation therapy |
| **Speaker Role** | Patient |
| **Elicitation Context** | Naturalistic, Controlled |
| **Recording Modality** | Smartphone |
| **Sample size** | 22 |
| **Mental health outcome** | Depression |
| **Outcome Biomarker Domain** | Speech rate, Pause duration/frequency, Pitch, Vocal intensity/loudness, MFCCs, Formant frequencies, Other |
| **Specific variables of marker studied** | Pitch variability, mean unvoiced-segment length, words per second, loudness, spectral flux, MFCCs, formant bandwidths |
| **clinical interpretability** | Yes |
| **Perceptial Label (if yes or Partial)** | Higher depression severity was associated with greater pitch variability, longer pauses, slower speech, and quieter voice |
| **Outcome Measure/Tool?** | ICD-10 clinical diagnosis, MADRS, momentary ADS-K, positive and negative affect, valence, energetic arousal, calmness |
| **Direction of Association** | Within patients, greater depressive symptoms were associated with higher pitch variability, longer pauses, and slower speech; improved affective states were generally associated with shorter pauses, faster speech, and louder voice |
| **Effect Size/Model Performance/Statistical Significance** | Pitch variability β=0.14, pauses β=0.10, speech rate β=-0.10 for depression severity; combined models retained pitch variability β=0.17 and pauses β=0.12 |

| | |
|---|---|
| **Main Author Conclusions** | Repeated smartphone speech samples captured within-person fluctuations in depression and affect, supporting their potential for individualized treatment monitoring |
| **Key Limitations** | Very small inpatient sample, 8 of 30 enrolled patients excluded, sleep deprivation intervention may limit generalizability, short prompted selfie recordings, some technical and missing-data exclusions, no external validation, modest explained variance |
| **Notes** | Patients recorded 10–20-second selfie videos for up to 26 days, producing 716 matched speech and affect assessments, averaging 32 assessments per patient. |

## Study 60 | Wang et al. (2025)

| | |
|---|---|
| **Study design** | Cross-sectional case-control observational interview study |
| **Setting** | Jimei University, China |
| **Population Type (adult, student, specific occupational groups)** | 70 Chinese college students, including 47 with subclinical depression and 23 controls |
| **Speaker Role** | Healthy Control |
| **Elicitation Context** | Controlled |
| **Recording Modality** | Other |
| **Sample size** | 70 |
| **Mental health outcome** | Depression |
| **Outcome Biomarker Domain** | Pause duration/frequency, Pitch, Prosody/rhythm, Verbal output, Sentiment/emotional tone, Other |
| **Specific variables of marker studied** | Pauses, hesitation, sighs, repetitions, stammering, speaking faster or slower, tone raising or lowering, helplessness, excitement and other vocal emotions |
| **clinical interpretability** | Yes |
| **Perceptial Label (if yes or Partial)** | More frequent and rapid prosodic changes, more sighing and repetition, and greater co-occurrence of pauses with hesitation and helplessness |
| **Outcome Measure/Tool?** | BDI-II-C, PHQ-9 |
| **Direction of Association** | The subclinical group showed more frequent but shorter prosodic changes, more sighing and repetition, longer repetition duration, and prominent pause-hesitation patterns; greater depression severity was associated with more frequent and shorter prosodic changes |
| **Effect Size/Model Performance/Statistical Significance** | Prosody annotation frequency d=0.60, p=.044; duration d=0.74, p=.026; BDI with prosody frequency r=0.26 and duration r=-0.37; no classification model reported |
| **Main Author Conclusions** | Manually annotated vocal emotion and prosody distinguished subclinical depression from controls and identified pause-hesitation, sighing, repetition and helplessness as potential indicators |
| **Key Limitations** | Small convenience sample of college students, subclinical symptoms based on screening scales rather than clinical diagnosis, gender not analyzed, subjective manual annotation, no acoustic signal measurements or external validation |
| **Notes** | Participants completed structured face-to-face interviews covering self, emotions, relationships and the future. Two coders annotated 1134 minutes of video with interrater kappa 0.81. |

## Study 61 | Wang et al. (2023)

| | |
|---|---|
| **Study design** | Cross-sectional prediction study with longitudinal treatment follow-up |

| | |
|---|---|
| **Setting** | Hainan Medical University, China |
| **Population Type (adult, student, specific occupational groups)** | 47 Chinese college students with depressive symptoms, including 18 who completed four weeks of internet-based cognitive-behavioral therapy |
| **Speaker Role** | Patient |
| **Elicitation Context** | Controlled |
| **Recording Modality** | Smartphone |
| **Sample size** | 47 |
| **Mental health outcome** | Depression |
| **Outcome Biomarker Domain** | Pitch, Vocal intensity/loudness, Voice quality, Formant frequencies, MFCCs, Other |
| **Specific variables of marker studied** | F0, creak, Peak-to-RMS, formants, MFCC deltas, mel-cepstral features, harmonic phase-distortion and glottal-source measures |
| **clinical interpretability** | Partial |
| **Perceptial Label (if yes or Partial)** | More severe depression was associated with altered loudness, greater creaky voice, and technical spectral and vocal-tract changes |
| **Outcome Measure/Tool?** | HAMD-17, PHQ-9 |
| **Direction of Association** | Thirty acoustic features correlated with depression severity; creak, mel-cepstral, MFCC and harmonic-phase measures were most predictive, while four features changed significantly after ICBT |
| **Effect Size/Model Performance/Statistical Significance** | Predicted HAMD correlation r=0.682, MAE=3.137; 63.8% had errors below 4 points; four acoustic features changed after ICBT |
| **Main Author Conclusions** | A neural-network model predicted depression severity from a brief standardized reading, and selected acoustic measures appeared sensitive to psychotherapy response |
| **Key Limitations** | Very small predominantly female student sample, depressive symptoms rather than confirmed MDD, no healthy control group, 18 completed follow-up, high-dimensional feature selection on the same dataset, leave-one-out validation only, no external validation |
| **Notes** | Participants read a standardized passage recorded on Android phones. After ICBT, HAMD scores improved from 8.79 to 0.52 and four Peak-to-RMS or MFCC-derived features decreased. |

## Study 62 | Wasserzug et al. (2023)

| | |
|---|---|
| **Study design** | Cross-sectional case-control study with longitudinal remission follow-up |
| **Setting** | Beer Yaakov-Ness Ziona Mental Health Center, Israel |
| **Population Type (adult, student, specific occupational groups)** | 144 Hebrew-speaking adults, including 40 patients with acute MDD and 104 nonclinical controls, with 14 patients reassessed in remission |
| **Speaker Role** | Patient, Healthy Control |
| **Elicitation Context** | Naturalistic |
| **Recording Modality** | Smartphone |
| **Sample size** | 144 |

| | |
|---|---|
| **Mental health outcome** | Depression |
| **Outcome Biomarker Domain** | Pitch, Vocal intensity/loudness, Pause duration/frequency, Prosody/rhythm, Other |
| **Specific variables of marker studied** | Proprietary pitch, amplitude, silence, duration, range, slope, frequency and shape parameters |
| **clinical interpretability** | No |
| **Perceptial Label (if yes or Partial)** | |
| **Outcome Measure/Tool?** | DSM-5 MDD diagnosis, HDRS-21, CGI |
| **Direction of Association** | Specific marker directions were not reported; vocal depression scores were higher in acute MDD than in remission or controls, and remained higher in remission than in controls |
| **Effect Size/Model Performance/Statistical Significance** | Test-recording accuracy 72.7%, sensitivity 55.1%, specificity 78.0%; participant-averaged accuracy 78.5%, sensitivity 60.0%, specificity 84.7%; vocal score and HDRS r=0.36 |
| **Main Author Conclusions** | A content-free smartphone voice model distinguished acute MDD from remission and nonclinical states, with improved performance when multiple recordings were averaged |
| **Key Limitations** | Small clinical sample, only 14 remission follow-ups, proprietary undisclosed features, recordings rather than participants used for model splitting, participant-averaged analysis mixed training and test data, routine treatment was not standardized, no independent external validation, company funding and involvement |
| **Notes** | Participants completed natural telephone conversations over two weeks, providing at least 45 seconds of speech per recording. The model used 2771 recordings and selected 15 parameters from more than 200 candidates. |

## Study 63 | Wei et al. (2025)

| | |
|---|---|
| **Study design** | Cross-sectional case-control machine-learning classification study |
| **Setting** | Xinxiang Medical University, Henan, China |
| **Population Type (adult, student, specific occupational groups)** | 206 Chinese university students, including 103 with depressive symptoms and 103 age-, sex-, and education-matched controls |
| **Speaker Role** | Healthy Control |
| **Elicitation Context** | Controlled |
| **Recording Modality** | Smartphone, Other |
| **Sample size** | 206 |
| **Mental health outcome** | Depression |
| **Outcome Biomarker Domain** | Pitch, Vocal intensity/loudness, Prosody/rhythm, Voice quality, Jitter, Shimmer, Formant frequencies, MFCCs, Other |
| **Specific variables of marker studied** | MFCCs, F0, energy, duration, voiced and unvoiced measures, jitter, shimmer, formant frequencies and bandwidths |
| **clinical interpretability** | Partial |
| **Perceptial Label (if yes or Partial)** | Reduced pitch variability, more monotonous speech, and greater instability in vocal intensity |

| | |
|---|---|
| **Outcome Measure/Tool?** | PHQ-9, GAD-7, Insomnia Severity Index |
| **Direction of Association** | Depression was associated with altered spectral, prosodic, and glottal features, including lower pitch variability and higher shimmer; MFCC features contributed most to classification |
| **Effect Size/Model Performance/Statistical Significance** | LDA AUC 0.771, accuracy 72.8%, precision 73.5%, recall 75.7%, F1 73.7% |
| **Main Author Conclusions** | A three-minute standardized reading and LDA model moderately distinguished students with depressive symptoms from matched controls |
| **Key Limitations** | Single-province convenience sample, PHQ-9 threshold rather than clinical diagnosis, no correction for multiple comparisons, controlled reading task, internal cross-validation only, no external validation, anxiety and insomnia differed between groups |
| **Notes** | Participants read the same neutral text for approximately three minutes. Thirty-two features differed between groups, and SHAP analysis identified MFCC measures as the most influential predictors. |

## Study 64 | Wiseman et al. (2025)

| | |
|---|---|
| **Study design** | Cross-sectional case-control observational study with cognitive assessment |
| **Setting** | Sunnybrook Health Sciences Centre and remote testing, Toronto, Canada |
| **Population Type (adult, student, specific occupational groups)** | 120 English-speaking adults, including 66 with treatment-resistant MDD and 54 nondepressed controls |
| **Speaker Role** | Patient, Healthy Control |
| **Elicitation Context** | Naturalistic |
| **Recording Modality** | Smartphone, Other |
| **Sample size** | 120 |
| **Mental health outcome** | Depression |
| **Outcome Biomarker Domain** | Speech rate, Pause duration/frequency, Pitch, Word or pronoun use, Sentiment/emotional tone, Other |
| **Specific variables of marker studied** | Mean pitch, pitch variability, speech rate, proportion of time pausing, language valence, emotional intensity, agency |
| **clinical interpretability** | Yes |
| **Perceptial Label (if yes or Partial)** | Lower-pitched and slower speech, more pausing, more negative and emotionally intense language, and less agentic language |
| **Outcome Measure/Tool?** | DSM-5 MDD diagnosis, HAMD-17, interleaved pro- and anti-saccade task |
| **Direction of Association** | MDD participants had lower pitch, slower speech, more pauses, more negative and intense language, and lower agency; speech features were not associated with HAMD-17 severity but several were associated with poorer executive function |
| **Effect Size/Model Performance/Statistical Significance** | Speech composite AUC 0.90; speech rate showed the largest individual group effect, $\eta^2=0.15$; composite effect $\eta^2=0.27$ |
| **Main Author Conclusions** | Speech features strongly distinguished treatment-resistant MDD from controls and appeared to capture executive difficulties not reflected by HAMD-17 severity scores |
| **Key Limitations** | Treatment-resistant moderate-to-severe MDD sample, cross-sectional design, controls lacked HAMD-17 scores, smaller cognitive-testing subset, predefined feature set, recording location varied, no longitudinal or external validation |

| | |
|---|---|
| **Notes** | Participants answered “Please tell me how you are feeling today.” Pitch variability did not differ between groups, while the combined linguistic and acoustic score provided the strongest discrimination. |

## Study 65 | Yamamoto et al. (2020)

| | |
|---|---|
| **Study design** | Multisite observational study with repeated longitudinal assessments |
| **Setting** | Keio University Hospital and seven collaborating clinical institutions, Japan |
| **Population Type (adult, student, specific occupational groups)** | 223 analyzed participants, including 84 with MDD, 68 with bipolar disorder, and 71 healthy controls |
| **Speaker Role** | Patient, Healthy Control |
| **Elicitation Context** | Naturalistic, Controlled |
| **Recording Modality** | Microphone |
| **Sample size** | 223 |
| **Mental health outcome** | Depression, Bipolar/Mania |
| **Outcome Biomarker Domain** | Speech rate, Pause duration/frequency, Response latency, Other |
| **Specific variables of marker studied** | Words per minute, within-speaker pause duration, response latency after interviewer speech |
| **clinical interpretability** | Yes |
| **Perceptial Label (if yes or Partial)** | Slower speech, longer pauses, and delayed responses |
| **Outcome Measure/Tool?** | DSM-5 diagnosis, HAMD-17, Young Mania Rating Scale |
| **Direction of Association** | MDD participants spoke more slowly, paused longer, and responded later than controls; all three measures correlated with depression severity and psychomotor retardation, while within-person speech-rate improvement tracked symptom improvement |
| **Effect Size/Model Performance/Statistical Significance** | Speech rate and HAMD r=-0.378, pause time r=0.298, response time r=0.458; change in speech rate and HAMD r=-0.317 |
| **Main Author Conclusions** | Timing measures from natural clinical interviews reflected depression severity, with speech rate also tracking longitudinal symptom change |
| **Key Limitations** | Healthy controls were older, medication effects and adverse effects could remain, environmental noise may affect recognition, individual speaking habits may confound results, repeated follow-up intervals varied, no predictive classifier or external validation |
| **Notes** | Participants completed repeated 10-minute unstructured interviews. The dataset included 972 usable interviews, and response time distinguished MDD from both healthy controls and bipolar disorder. |

## Study 66 | Yang et al. (2023)

| | |
|---|---|
| **Study design** | Cross-sectional case-control autobiographical memory study |
| **Setting** | University of Electronic Science and Technology of China and affiliated clinical hospital, Chengdu, China |

| | |
|---|---|
| **Population Type (adult, student, specific occupational groups)** | 80 Chinese adults, including 40 first-episode untreated patients with MDD and 40 age- and sex-matched healthy controls |
| **Speaker Role** | Patient, Healthy Control |
| **Elicitation Context** | Controlled |
| **Recording Modality** | Microphone |
| **Sample size** | 80 |
| **Mental health outcome** | Depression |
| **Outcome Biomarker Domain** | Speech rate, Pause duration/frequency, Verbal output, Word or pronoun use, Sentiment/emotional tone, Other |
| **Specific variables of marker studied** | Articulation rate, syllable count, pause duration, pause number, first-person pronouns, tense, emotion, family, work, health, death, causation and other LIWC word categories |
| **clinical interpretability** | Yes |
| **Perceptial Label (if yes or Partial)** | Slower speech, less verbal output, and greater use of negative-emotion, family, health and self-focused language |
| **Outcome Measure/Tool?** | DSM-5 MDD diagnosis, BDI-II |
| **Direction of Association** | MDD participants spoke more slowly and produced fewer syllables across all emotional contexts; word-use differences varied by emotion, and a combined speech-language model explained substantial variance in depression severity |
| **Effect Size/Model Performance/Statistical Significance** | Articulation-rate group effect $\eta^2p=0.467$, syllable-count effect $\eta^2p=0.157$; 10-predictor model adjusted $R^2=0.675$ and explained 71.6% of BDI-II variance |
| **Main Author Conclusions** | Emotion-specific autobiographical speech and language features strongly differentiated MDD from controls and improved estimation of depression severity |
| **Key Limitations** | Small single-site sample, dictionary-based text analysis omitted some words, stepwise regression and many comparisons increase overfitting risk, 30-second constrained memories, no independent validation, no cognitive testing |
| **Notes** | Participants recalled six personal memories under sad, angry, fearful, neutral and happy cues. Articulation rate in sad and happy conditions and emotion-specific word categories formed the final severity model. |

## Study 67 | Zhang et al. (2020)

| | |
|---|---|
| **Study design** | Online cross-sectional machine-learning prediction study |
| **Setting** | Mental Health America and NeuroLex Laboratories online platforms, United States |
| **Population Type (adult, student, specific occupational groups)** | 222 anonymous online participants who completed a depression screen and provided at least one usable voice recording |
| **Speaker Role** | Healthy Control |
| **Elicitation Context** | Naturalistic, Controlled |
| **Recording Modality** | Other |
| **Sample size** | 222 |
| **Mental health outcome** | Depression, Other |

| | |
|---|---|
| **Outcome Biomarker Domain** | Pause duration/frequency, Pitch, Vocal intensity/loudness, Verbal output, Formant frequencies, Harmonics-to-noise ratio, MFCCs, Word or pronoun use, Prosody/rhythm, Other |
| **Specific variables of marker studied** | F0, loudness, HNR, formants, MFCCs, spectral measures, pause length and variability, pause-to-speech ratio, speaking time, word-frequency and TF-IDF vectors |
| **clinical interpretability** | Partial |
| **Perceptial Label (if yes or Partial)** | Altered pausing, speaking amount, pitch and loudness patterns, with differences in word use |
| **Outcome Measure/Tool?** | PHQ-9 |
| **Direction of Association** | Voice and linguistic features predicted depression severity and suicidal ideation better than psychomotor disturbance; pause measures and technical acoustic features showed small associations with PHQ-9 outcomes |
| **Effect Size/Model Performance/Statistical Significance** | Suicidal ideation TF-IDF AUC 0.821, acoustic AUC 0.783, prosody AUC 0.751; depression severity MAE 4.66–4.78; psychomotor disturbance best AUC 0.613 |
| **Main Author Conclusions** | Brief remotely collected voice samples showed moderate utility for estimating depression severity and identifying self-reported suicidal ideation |
| **Key Limitations** | Anonymous self-selected online sample, PHQ-9 without clinical diagnosis, inconsistent recording devices and environments, substantial missing demographics, many adolescent or unknown-age participants, synthetic oversampling, internal cross-validation only, no external validation, company funding and conflict of interest |
| **Notes** | Participants repeated a fixed sentence and provided up to 30 seconds of free speech. Suicidal ideation was coded Other, and free speech generally performed better for estimating depression severity. |

## Study 68 | Zhao et al. (2022)

| | |
|---|---|
| **Study design** | Cross-sectional case-control machine-learning classification study |
| **Setting** | Beijing Huilongguan Hospital and surrounding community, Beijing, China |
| **Population Type (adult, student, specific occupational groups)** | 133 Chinese adults, including 71 patients with depression and 62 healthy controls |
| **Speaker Role** | Patient, Healthy Control |
| **Elicitation Context** | Controlled |
| **Recording Modality** | Microphone |
| **Sample size** | 133 |
| **Mental health outcome** | Depression, Anxiety |
| **Outcome Biomarker Domain** | Pitch, Harmonics-to-noise ratio, MFCCs, Other |
| **Specific variables of marker studied** | F0, HNR, zero-crossing rate, MFCC1–12 across positive, neutral, and negative reading tasks |
| **clinical interpretability** | Partial |
| **Perceptial Label (if yes or Partial)** | Altered pitch and voice quality across emotional reading tasks, with technical spectral differences linked to symptom severity |
| **Outcome Measure/Tool?** | DSM-IV depression diagnosis, HAMD-17, HAMA, PHQ-9 |

| | |
|---|---|
| **Direction of Association** | All acoustic features differed between groups; MFCC4 and MFCC7 correlated with PHQ-9 severity, ZCR correlated with anxiety, and MFCC9 correlated with HAMD anxiety and somatization |
| **Effect Size/Model Performance/Statistical Significance** | Logistic regression accuracy 89.66%, AUC 0.98, depression precision 0.89, recall 0.94; SVM accuracy 86.21%, AUC 0.95 |
| **Main Author Conclusions** | Acoustic features from emotion-specific reading tasks showed strong internal discrimination of depression and modest associations with depressive and anxiety symptoms |
| **Key Limitations** | Cross-sectional single-site design, Chinese-only sample, simple controlled reading paradigm, limited clinical characterization, possible medication effects not clearly addressed, feature selection and model development in a modest sample, internal validation only, no independent external validation |
| **Notes** | Participants read positive, neutral, and negative passages. MFCC7 from the negative task predicted PHQ-9 scores, while MFCC9 from the neutral task predicted HAMD anxiety and somatization. |

# Appendix — Legend and Decision Rules

| **Legend & Decision Rules — Voice Biomarker Scoping Review Extraction Form** | | | |
|---|---|---|---|
| This sheet defines every column in the Extraction Form and every dropdown option | | | |
| SECTION 1 — Column Definitions (Columns A to U) | | | |
| Column & name | What to record here | Plain-language explanation | Example entry |
| A — Study ID | A unique number assigned to each paper you include. | Reference number so you can track which row belongs to which paper. Numbers are pre-filled. | 1, 2, 3 … |
| B — Authors | The surname(s) of the paper's author(s). | Use the surname only. If there are more than two authors, write the first author's surname followed by 'et al.' | Smith; Smith & Jones; Smith et al. |
| C — Year | Year the paper was published. | Year of publication | 2023 |
| D — Study Design | The type of research design used in the paper. | This describes how the study was structured. Common types: cross-sectional (one point in time), longitudinal (followed over time), RCT (randomised controlled trial), case-control (compares people with and without a condition), observational (no intervention, just observation). | Cross-sectional; Longitudinal; RCT; Case-control |
| E — Setting | The real-world location or environment where data were collected. | Where did the study take place? Examples: a hospital clinic, a university psychology lab, a workplace, an online/app-based platform. | Outpatient clinic; University lab; Workplace; Online/app |
| F — Population Type | A description of who was in the study sample. | Describe the participants — their age group, clinical characteristics, or occupation. Be as specific as the paper allows. | Adults with major depression; Medical residents; General adult community sample; University students |
| G — Speaker Role ■ | Who is speaking in the voice/speech recordings being analysed. | This tells us whose voice is being studied. Is it a patient speaking about their symptoms? A doctor speaking during a consultation? A healthy volunteer for comparison? See Section 2 for full option definitions. | Dropdown — see Section 2 |
| H — Elicitation Context ■ | How the speech was obtained or recorded. | Was the speaker asked to do a specific task in a quiet room (controlled), having a real conversation (naturalistic), or was their speech captured passively without them knowing a specific recording task was happening (passive/ambient)? See Section 3. | Dropdown — see Section 3 |
| I — Recording Modality ■ | The device or technology used to capture the speech. | This is simply the equipment used: a lab microphone, a smartphone, a telephone call recording, a wearable device, a video camera, etc. See Section 4. | Dropdown — see Section 4 |

| Legend & Decision Rules — Voice Biomarker Scoping Review Extraction Form | | | |
|---|---|---|---|
| J — Sample Size | The total number of participants in the study. | Record the number of people whose data were analysed. If the paper reports different numbers for different groups, record the total. | 45; 312; 1,204 |
| K — Mental Health Outcome ■ | The mental health or psychological condition being measured in the study. | What psychological state or condition is the study trying to detect or predict using voice/speech markers? See Section 5 for definitions of each option. | Dropdown — see Section 5 |
| L — Biomarker Domain ■ | The specific type of voice, speech, or language feature being measured. | This is the actual marker extracted from the recording — for example, how fast someone speaks (speech rate), the pitch of their voice, or how negative their words are (sentiment). Choose the option that best matches the marker described in the paper. If a paper reports multiple markers, add one row per marker. See Section 6 for full definitions. | Dropdown — see Section 6 |
| M — Specific Marker(s) Studied | The exact name of the marker(s) as written in the paper. | Copy or closely paraphrase the marker name as the authors describe it. If multiple markers are reported, list them separated by semicolons. This captures detail beyond the dropdown category. | Mean F0 (Hz); Words per minute; MFCC coefficients 1–13 |
| N — Clinical Interpretability ■ | Whether a clinician could understand or perceive this marker without needing a computer readout. | Some markers can be heard or noticed by a trained listener (e.g. a flat, monotone voice). Others can only be measured by software (e.g. mathematical signal features). Select Yes, Partial, or No. See Section 7 for the decision rule. | Dropdown — Yes / Partial / No |
| O — Perceptual Label (if Yes or Partial) | If you selected Yes or Partial in Column N, describe what the marker sounds like in plain clinical language. | Write what a doctor or nurse would hear or notice in the patient's voice. Leave blank if you selected No in Column N. | Flat monotone voice; Slow hesitant speech; Rough or hoarse voice quality |
| P — Outcome Measure / Tool | The questionnaire, scale, or diagnostic method used to measure the mental health outcome. | How did the study measure depression, burnout, etc.? Record the full name and abbreviation of any validated tool used. If a clinical diagnosis was made (e.g. DSM-5), record that. | Patient Health Questionnaire-9 (PHQ-9); Maslach Burnout Inventory (MBI); DSM-5 clinical diagnosis |
| Q — Direction of Association | How the biomarker changed in relation to the mental health outcome. | Describe the direction of the finding: did the marker go up or down when the condition was present or more severe? Write this in plain language as reported in the paper. | Decreased speech rate with higher depression scores; Increased pause frequency in burnout group |
| R — Effect Size / Model Performance / Statistical Significance | The numerical result(s) reported to support the finding. | Record the specific number(s) reported. For correlations: r value. For comparisons: odds ratio (OR) or Cohen's d. For machine learning models: accuracy, AUC, sensitivity, specificity. Always include p-values or confidence intervals if reported. | $r = -0.45$, $p < 0.001$; AUC = 0.82; OR = 2.3 (95% CI 1.4–3.8) |
| S — Main Author Conclusions | A brief summary of what the authors concluded about their findings. | In your own words, summarise what the authors said their results mean. You do not need to copy verbatim — a 1–2 sentence summary is sufficient. | Authors concluded that reduced speech rate is a reliable acoustic marker of depressive symptom severity and may be useful for monitoring treatment response. |

| Legend & Decision Rules — Voice Biomarker Scoping Review Extraction Form | | | |
|---|---|---|---|
| T — Key Limitations | The main weaknesses of the study as reported by the authors. | Most papers include a limitations section. Record the most important ones — for example: small sample size, only one time point measured, lab conditions that may not reflect real life, no validation in a separate group. | Small sample (n=32); Controlled lab setting only; No external validation; Self-report outcome measure |
| U — Notes | Any additional comments, flags for the second reviewer, or relevance to the ambient scribe study. | Use this for anything that does not fit in the other columns — e.g. if the study recorded both the clinician and patient, if there is ambiguity in classification, or if the study is particularly relevant to detecting burnout using an ambient AI scribe. | Both clinician and patient voice captured — highly relevant to ambient scribe study design. |
| SECTION 2 — Dropdown Options: Speaker Role (Column G) | | | |
| Option | Who this refers to | When to use this option | Example |
| Patient | The person who has, or is at risk of, a mental health condition — the one being assessed or treated. | Use this when the voice being analysed belongs to the person whose mental health is being studied (e.g. a person with depression speaking in an interview). | A patient with major depressive disorder completing a structured interview |
| Clinician | A healthcare professional — doctor, nurse, therapist, resident — whose own speech is being analysed. | Use this when the study is looking at the clinician's voice, not the patient's — e.g. a study measuring whether a burned-out physician speaks differently. | Emergency physicians' speech patterns analysed for burnout markers |
| Healthy control | A person without a mental health diagnosis, included in the study for comparison purposes. | Use this when the speaker has no known mental health condition and is serving as a comparison group against those who do. | Age-matched community volunteers with no history of depression or anxiety |
| Mixed/dyadic (2 person conversation) | A two-person conversation where both speakers' voices are captured and/or analysed together. | Use this when the recording includes a back-and-forth conversation (e.g. a therapy session or clinical consultation) and the study analyses both speakers, or does not separate them. | A clinician-patient consultation recorded and analysed as a dyadic interaction |
| Other | Any speaker role not covered by the above categories. | Use this if the speaker type is unusual or unclearly defined — and add a note in Column U. | An automated voice assistant; a voice actor used as a stimulus |
| SECTION 3 — Dropdown Options: Elicitation Context (Column H) | | | |
| Option | What this means | When to use this option | Relevance to ambient scribe |
| Controlled | Speech was recorded under carefully structured conditions, usually in a quiet room with a specific task — like reading a passage aloud, saying a sustained vowel sound, or answering scripted interview questions. | Use this when the study gave participants a specific verbal task to complete and controlled the recording environment (e.g. a soundproof booth, a lab). | Low. Controlled lab conditions are very different from the noisy, unscripted environment of a real clinical encounter — so findings may not directly apply to what an ambient scribe would capture. |
| Naturalistic | Speech was recorded during a real or semi-real conversation without a scripted task — for example, a clinical interview, a therapy session, or a research conversation that unfolded naturally. | Use this when the recording involved genuine back-and-forth conversation in a realistic setting, even if a researcher was present and directing the session. | Moderate. More similar to real clinical conversations, but a researcher is still usually present and the setting may still be more structured than a typical clinic. |

| **Legend & Decision Rules — Voice Biomarker Scoping Review Extraction Form** | | | |
|---|---|---|---|
| Passive/Ambient | Speech was captured continuously or passively in an everyday setting, without the speaker being asked to complete a specific speech task — the recording just 'listens in' on real life. | Use this when data were collected using always-on recording (e.g. a room microphone, a passive smartphone app, or an AI scribe running during a clinic appointment). | High. This is the most directly relevant to our clinical study — it mirrors exactly how an ambient AI scribe would capture speech during a real clinical encounter. |
| SECTION 4 — Dropdown Options: Recording Modality (Column I) | | | |
| Option | What it is | Notes on audio quality / relevance | |
| Microphone | A dedicated close-range or studio-quality microphone placed near the speaker, typically used in lab or clinical interview settings. | Produces the highest audio quality and lowest background noise. Most common in controlled studies. May not reflect what an ambient scribe (which records from a distance) would capture. | |
| Smartphone | The built-in microphone of a smartphone, either held near the speaker or placed on a desk. | Common in passive sensing and ecological momentary assessment (EMA) studies. Variable audio quality depending on placement and environment. | |
| Telephone | Speech captured via a telephone call — either a landline or mobile phone call recording. | Telephone audio is compressed and bandwidth-limited, which affects the acoustic quality. Relevant to telemedicine and telehealth consultations. | |
| Wearable | A microphone embedded in a wearable device, such as a smartwatch, a lapel/clip-on microphone worn on clothing, or an earbud. | Often captures cleaner audio than a room microphone because it is closer to the speaker's mouth. Relevant to personal health monitoring. | |
| Video | Audio extracted from a video recording — the sound track of a video file. | Audio quality depends on whether a dedicated microphone was used alongside the camera, or just the camera's built-in mic. Video allows analysis of visual cues too. | |
| Other | Any recording device not listed above. | Add a note in Column U describing the device used. | |
| SECTION 5 — Dropdown Options: Mental Health Outcome (Column K) | | | |
| Option | Clinical definition | How it is typically measured | |
| Depression | A mood disorder characterised by persistent low mood, loss of interest or pleasure, fatigue, and feelings of worthlessness, lasting at least two weeks. | Validated questionnaires such as the Patient Health Questionnaire-9 (PHQ-9), Beck Depression Inventory (BDI), or Hamilton Depression Rating Scale (HAM-D); or clinical diagnosis using DSM-5 criteria. | |
| Anxiety | A mental health condition involving excessive worry, fear, or nervousness that is difficult to control and interferes with daily functioning. | Generalised Anxiety Disorder scale (GAD-7), State-Trait Anxiety Inventory (STAI), Hamilton Anxiety Rating Scale (HAM-A). | |

| **Legend & Decision Rules — Voice Biomarker Scoping Review Extraction Form** | | | |
|---|---|---|---|
| Stress | A state of psychological strain or tension arising from demanding circumstances — not a clinical diagnosis, but a dimensional construct that can range from mild to severe. | Perceived Stress Scale (PSS), visual analogue scales, or physiological measures (cortisol). May also be self-reported. | |
| Burnout | A work-related syndrome with three components: emotional exhaustion (feeling depleted), depersonalisation (becoming detached or cynical towards patients), and reduced personal accomplishment. Particularly common in healthcare workers. | Maslach Burnout Inventory (MBI) — the most widely used tool. Also: Oldenburg Burnout Inventory (OLBI), single-item burnout scales. | |
| Emotional Exhaustion | The feeling of being emotionally drained and depleted — often considered the core dimension of burnout. Can be measured separately from full burnout. | MBI Emotional Exhaustion subscale; Emotional Exhaustion Scale; items from burnout inventories. | |
| Fatigue | A subjective sense of tiredness, lack of energy, or exhaustion — which may be physical, mental, or both. Distinct from normal tiredness as it is persistent and not fully relieved by rest. | Fatigue Severity Scale (FSS), Multidimensional Fatigue Inventory (MFI), Visual Analogue Scale for Fatigue (VAS-F). | |
| Well-being | A positive state of mental health encompassing life satisfaction, positive emotions, a sense of meaning, and the absence of distress. Often used as the opposite pole to burnout or depression. | Warwick-Edinburgh Mental Well-being Scale (WEMWBS), WHO-5 Well-being Index, Positive and Negative Affect Schedule (PANAS). | |
| Distress | A broad term for psychological suffering or emotional pain — can refer to general psychological distress, or distress in a specific context (e.g. cancer-related distress, occupational distress). Not a single diagnosis. | Kessler Psychological Distress Scale (K10), General Health Questionnaire (GHQ), Distress Thermometer. | |
| Other | Any mental health or psychological outcome not listed above (e.g. PTSD, schizophrenia, suicidality, cognitive impairment). | Record the specific outcome in Column M (Specific Marker(s)) and note the measurement tool in Column P. | |
| SECTION 6 — Dropdown Options: Biomarker Domain (Column L) | | | |
| Option | What it measures / what it is | Plain-language description (what you would hear or see) | Clinical interpretability |
| Speech rate | How fast or slowly someone speaks — typically measured as words per minute (WPM) or syllables per second. | A clinician would notice this as speaking unusually slowly, as if struggling to find words, or speaking very rapidly in a pressured or agitated way. | Yes — directly perceivable by a trained listener. |
| Pause duration/frequency | How long the silent gaps between words or phrases are, and/or how often they occur. | Long, frequent silences mid-sentence or between answers. In depression, pauses tend to be longer and more frequent than in healthy speech. | Yes — a clinician would notice unusual silences or hesitations. |
| Pitch | The perceived highness or lowness of the voice — technically measured as fundamental frequency (F0) in Hertz (Hz). | A low, flat pitch sounds monotone and expressionless. Elevated pitch can sound tense or anxious. Depression is often associated with a lower mean pitch. | Yes — pitch is directly perceivable by ear. |

| Legend & Decision Rules — Voice Biomarker Scoping Review Extraction Form | | | |
|---|---|---|---|
| Vocal intensity/loudness | How loud or soft the voice is — measured as amplitude or decibels (dB). | A quiet, soft, or barely audible voice. People with depression or burnout may speak with less energy and reduced loudness. | Yes — a clinician can clearly hear a quiet or loud voice. |
| Prosody/rhythm | The overall melodic and rhythmic pattern of speech — the 'music' of how someone talks, including rises and falls in pitch, stress on syllables, and timing. | Flat, monotone speech that sounds robotic or expressionless — lacking the natural melody and variation of healthy speech. | Yes — altered prosody is noticeable to a listener as sounding 'flat' or 'robotic'. |
| Voice quality (hoarseness/breathiness) | The perceived texture or quality of the voice — whether it sounds smooth, rough, hoarse, breathy, or strained. | A rough or gravelly voice (hoarseness), or a whispery, airy voice (breathiness). These qualities reflect how well the vocal cords are vibrating. | Yes — voice quality is directly perceivable by ear without any measurement tools. |
| Response latency | The time delay between when a question or prompt is given and when the speaker begins to respond. | A long pause before the person starts talking — as if they are slow to process or formulate a response. Common in depression and cognitive fatigue. | Yes — a clinician notices if a patient takes an unusually long time to respond. |
| Verbal output | The total amount of speech produced — how many words, sentences, or how long the person spoke in total. | Reduced verbal output means the person gives very short, brief answers. People with depression often say very little compared to healthy controls. | Yes — a clinician would notice if a patient gives only very brief responses. |
| Jitter | A technical acoustic measure of how much the pitch (frequency) varies from one vocal cycle to the next — tiny irregularities in pitch cycles measured in milliseconds. | High jitter corresponds to a rough, irregular, or unsteady-sounding voice. Requires software to measure — a clinician cannot detect 'jitter' directly but may perceive the resulting roughness. | Partial — a human hears the roughness, but 'jitter' itself is a software measurement. |
| Shimmer | A technical acoustic measure of how much the loudness (amplitude) varies from one vocal cycle to the next. | High shimmer corresponds to a weak, unstable, or breathy-sounding voice. Like jitter, requires software — a clinician perceives the breathiness or instability, not the shimmer value itself. | Partial — a human hears the instability or breathiness, but shimmer is a software measurement. |
| Harmonics-to-noise ratio (HNR) | The ratio of the periodic (harmonic) sound produced by the vocal cords to the random (noise) component — a measure of how 'clean' or 'noisy' the voice is. | A low HNR means the voice sounds breathy, hoarse, or rough — as if air is leaking through the vocal cords. A high HNR means a clear, clean voice. | Partial — the resulting voice quality (breathy/clear) is perceivable, but HNR requires software to calculate. |
| Formant frequencies | The resonance peaks produced by the shape of the vocal tract (mouth, throat) — particularly F1 and F2, which determine vowel sounds and articulation quality. | Changes in formants affect vowel clarity and articulation. Altered formants may sound like mumbled, unclear, or poorly articulated speech — though the frequency values themselves require software. | Partial — altered articulation may be noticeable, but formant frequencies require software to measure. |
| Sentiment/emotional tone | The emotional valence of the words used — whether the language is predominantly positive, negative, or neutral. Derived from the content of what is said, using natural language processing (NLP). | A clinician might notice a patient using many negative words ('hopeless', 'worthless', 'tired') or very few positive words. Sentiment analysis quantifies this pattern from a transcript. | Yes — a clinician can perceive and recognise negative or positive emotional language. |
| Word use/pronoun use | Patterns in the types of words used — particularly personal pronouns. Research shows that people with depression use more first-person singular pronouns ('I', 'me', 'my') and fewer social pronouns ('we', 'us'). | A clinician might notice a patient speaking only about themselves, rarely referring to others, or using very self-focused language. | Yes — a clinician can notice self-focused or socially withdrawn language patterns. |

| **Legend & Decision Rules — Voice Biomarker Scoping Review Extraction Form** | | | |
|---|---|---|---|
| Linguistic complexity | How grammatically complex and varied the spoken language is — measured using metrics such as sentence length, vocabulary diversity (type-token ratio), and syntactic complexity. | Simplified or repetitive language — using the same words repeatedly, short simple sentences, or limited vocabulary. This can reflect cognitive fatigue or reduced mental effort. | Yes — a clinician can perceive very simple or reduced speech, though formal measurement requires software. |
| Communication behaviour | Observable patterns of interaction between two people — how they take turns speaking, whether one person interrupts the other, levels of expressed empathy, and conversational engagement. | A clinician might notice if a patient rarely makes eye contact, gives very short answers, rarely asks questions, or seems disengaged from the conversation. | Yes — communication behaviours are directly observable in a conversation. |
| Mel-frequency cepstral coefficients (MFCC) | A set of mathematical features derived from the audio signal that represent the shape of the short-term power spectrum of the voice — a standard tool in speech recognition and machine learning. | MFCCs have no plain-language perceptual meaning — there is nothing a clinician could hear and describe as 'high MFCC coefficient 3'. They are purely computational features used to train machine learning models. | No — MFCCs are technical/engineering features with no human perceptual correlate. |
| Deep learning/audio embeddings | Numerical feature vectors automatically learned by a deep learning model (e.g. a neural network) from raw audio data — a highly abstract mathematical representation of the sound. | Like MFCCs, embeddings have no plain-language meaning. They capture complex patterns in audio that a model has learned to associate with outcomes, but cannot be described or perceived by a clinician. | No — deep learning embeddings are technical features with no human perceptual correlate. |
| Other | Any voice, speech, or language marker not covered by the options above. | Record the specific marker name in Column M and describe it in Column U. | Assess interpretability separately and record in Column N. |
| SECTION 7 — Dropdown Options & Decision Rule: Clinical Interpretability (Column N) | | | |
| Rating | What it means | Reviewer decision question — ask yourself this | Examples |
| Yes | A trained clinician, psychologist, or health professional could hear or describe this marker in plain, everyday language — without needing a computer to give them a number. | Could a doctor or nurse notice and describe this feature just by listening to the patient speak, without any special equipment or software? If YES → select 'Yes'. | Speech rate (slow/fast speech), pause duration (long silences), pitch (flat/monotone), loudness (soft voice), verbal output (brief answers), sentiment (negative language), communication behaviour (disengaged) |
| Partial | The marker can only be measured using software, but the result corresponds to something a clinician would recognise if told what to listen for — it has a real-world perceptual equivalent. | Does this software measurement correspond to a voice quality a clinician would be able to hear and describe if it were pointed out to them? If YES → select 'Partial', and describe the perceived quality in Column O. | Jitter → 'rough or unsteady voice'; Shimmer → 'breathy or weak voice'; HNR → 'hoarse or noisy voice'; Formants → 'unclear articulation or mumbled speech' |
| No | This is a purely technical or mathematical feature of the audio signal — there is no perceptual or clinical description a clinician would use to describe what they hear. It only exists as a number produced by software. | Is this feature a mathematical transformation of the audio signal with no plain-language perceptual equivalent? Could a clinician not describe it by listening? If YES → select 'No', and leave Column O blank. | MFCCs, cepstral coefficients, spectral centroid, spectral flux, deep learning audio embeddings, latent neural network features |
