# Supplementary material for: Voice and speech as digital biomarkers of mental health: a scoping review of clinical interpretability, data collection and translational readiness
Source: Front Digit Health. 2026 Oct 5;8:1964389. doi: 10.3389/fdgth.2026.1964389 (PMC13677924; doi:10.3389/fdgth.2026.1964389)
Supplement: Supplementary file 2 [file Table1.pdf]

Supplementary Materials

Table S1: Complete database search strategies

Final search date: June 18, 2026. No publication-year restriction was applied. Searches were limited to English-language publications as specified below.

Database / platform	Date searched	Limits / fields	Complete search strategy	Records retrieved
**PubMed**	June 18, 2026	English; humans filter via NOT animals-only set	(<br>"voice biomarker*"[tiab] OR "speech biomarker*"[tiab] OR "vocal biomarker*"[tiab]<br>OR "voice marker*"[tiab] OR "speech marker*"[tiab] OR "vocal marker*"[tiab]<br>OR "voice feature*"[tiab] OR "speech feature*"[tiab] OR "acoustic feature*"[tiab]<br>OR "voice analysis"[tiab] OR "speech analysis"[tiab] OR "acoustic analysis"[tiab]<br>OR "speech signal*"[tiab] OR "voice signal*"[tiab]<br>OR "vocal acoustic*"[tiab] OR "speech acoustic*"[tiab]<br>OR "prosodic feature*"[tiab] OR "paralinguistic feature*"[tiab]<br>OR acoustic*[tiab] OR prosod*[tiab] OR paralinguistic*[tiab]<br>)<br>AND<br>(<br>"Mental Disorders"[Mesh] OR "Mental Health"[Mesh] OR "Depression"[Mesh] OR "Anxiety"[Mesh]<br>OR "Stress, Psychological"[Mesh] OR "Stress Disorders, Post-Traumatic"[Mesh]<br>OR "Burnout, Professional"[Mesh] OR "Fatigue"[Mesh]<br>OR depression[tiab] OR depressive[tiab] OR anxiety[tiab]<br>OR "psychological stress"[tiab] OR "psychological distress"[tiab] OR "emotional distress"[tiab]<br>OR PTSD[tiab] OR "post-traumatic stress"[tiab] OR "posttraumatic stress"[tiab]<br>OR burnout[tiab] OR "burn-out"[tiab] OR "emotional exhaustion"[tiab]<br>OR fatigue[tiab] OR "cognitive fatigue"[tiab]<br>OR "mental health"[tiab]<br>)<br>AND<br>(<br>"Biomarkers"[Mesh] OR "Artificial Intelligence"[Mesh] OR "Machine Learning"[Mesh] OR "Algorithms"[Mesh]<br>OR biomarker*[tiab] OR "digital biomarker*"[tiab]<br>OR predict*[tiab] OR detect*[tiab] OR classif*[tiab] OR monitor*[tiab] OR screen*[tiab]<br>OR "machine learning"[tiab] OR "artificial intelligence"[tiab] OR algorithm*[tiab]<br>)<br>AND english[la]<br>NOT<br>(<br>animals[mh] NOT humans[mh]<br>)	176
**PsycINFO via EBSCO**	June 18, 2026	Advanced Search; all three boxes searched in Abstract (AB); English-language inclusion criterion	Advanced Search; each search box set to AB Abstract.<br><br>Box 1 - AB Abstract<br>"voice biomarker*" OR "speech biomarker*" OR "vocal biomarker*" OR "voice marker*" OR "speech marker*" OR "vocal marker*" OR "voice feature*" OR "speech feature*" OR "acoustic feature*" OR "voice analysis" OR "speech analysis" OR "acoustic analysis" OR "speech signal*" OR "voice signal*" OR "vocal acoustic*" OR "speech acoustic*" OR "prosodic feature*" OR "paralinguistic feature*" OR acoustic* OR prosod* OR paralinguistic*<br><br>Box 2 - AB Abstract<br>depression OR depressive OR anxiety OR "psychological stress" OR "psychological distress" OR "emotional distress" OR PTSD OR "post-traumatic stress" OR "posttraumatic stress" OR burnout OR "burn-out" OR "emotional exhaustion" OR "mental health" OR fatigue OR "cognitive fatigue"<br><br>Box 3 - AB Abstract<br>biomarker* OR "digital biomarker*" OR predict* OR detect* OR classif* OR classify OR classification OR monitor* OR screen* OR "machine learning" OR "artificial intelligence" OR algorithm*<br><br>Combination<br>Box 1 AND Box 2 AND Box 3	414

**Scopus**	June 18, 2026	TITLE/ABS search; English-language limit; specified neurological/developmental exclusions	(<br>TITLE(<br>"voice biomarker*" OR "speech biomarker*" OR "vocal biomarker*" OR<br>"digital voice biomarker*" OR "digital speech biomarker*" OR<br>"voice analysis" OR "speech analysis" OR "acoustic analysis" OR<br>"voice feature*" OR "speech feature*" OR "acoustic feature*" OR<br>"prosodic feature*" OR "paralinguistic feature*" OR<br>"speech signal*" OR "voice signal*"<br>)<br>OR<br>ABS(<br>"voice biomarker*" OR "speech biomarker*" OR "vocal biomarker*" OR<br>"digital voice biomarker*" OR "digital speech biomarker*" OR<br>"voice analysis" OR "speech analysis" OR "acoustic analysis" OR<br>"voice feature*" OR "speech feature*" OR "acoustic feature*" OR<br>"prosodic feature*" OR "paralinguistic feature*" OR<br>"speech signal*" OR "voice signal*"<br>)<br>)<br>AND<br>(<br>TITLE(<br>depression OR depressive OR anxiety OR "psychological stress" OR<br>"psychological distress" OR "emotional distress" OR PTSD OR<br>"post-traumatic stress" OR "posttraumatic stress" OR burnout OR<br>"burn-out" OR "emotional exhaustion" OR "mental health" OR<br>fatigue OR "cognitive fatigue"<br>)<br>OR<br>ABS(<br>depression OR depressive OR anxiety OR "psychological stress" OR<br>"psychological distress" OR "emotional distress" OR PTSD OR<br>"post-traumatic stress" OR "posttraumatic stress" OR burnout OR<br>"burn-out" OR "emotional exhaustion" OR "mental health" OR<br>fatigue OR "cognitive fatigue"<br>)<br>)<br>AND<br>(<br>TITLE(<br>biomarker* OR "digital biomarker*" OR predict* OR detect* OR classify OR<br>classification OR screen* OR monitor* OR "machine learning"<br>)<br>OR<br>ABS(<br>biomarker* OR "digital biomarker*" OR predict* OR detect* OR classify OR<br>classification OR screen* OR monitor* OR "machine learning"<br>)<br>)<br>AND NOT<br>(<br>TITLE(<br>aphasia OR dysarthria OR "speech disorder*" OR "language disorder*" OR<br>dementia OR Alzheimer* OR Parkinson* OR autism OR stroke OR "cognitive impairment"<br>)<br>OR<br>ABS(<br>aphasia OR dysarthria OR "speech disorder*" OR "language disorder*" OR<br>dementia OR Alzheimer* OR Parkinson* OR autism OR stroke OR "cognitive impairment"<br>)<br>)<br>AND<br>(<br>LIMIT-TO(LANGUAGE, "English")<br>)	934
